# 1,2,5,6‐Tetrakis(guanidino)‐Naphthalenes: Electron Donors, Fluorescent Probes and Redox‐Active Ligands

**DOI:** 10.1002/chem.201905471

**Published:** 2020-04-21

**Authors:** Lukas Lohmeyer, Elisabeth Kaifer, Hubert Wadepohl, Hans‐Jörg Himmel

**Affiliations:** ^1^ Anorganisch-Chemisches Institut Ruprecht-Karls-Universität Heidelberg Im Neuenheimer Feld 270 69120 Heidelberg Germany

**Keywords:** aromatic substitution, electron donors, guanidines, oxidation, protonation

## Abstract

New redox‐active 1,2,5,6‐tetrakis(guanidino)‐naphthalene compounds, isolable and storable in the neutral and deep‐green dicationic redox states and oxidisable further in two one‐electron steps to the tetracations, are reported. Protonation switches on blue fluorescence, with the fluorescence intensity (quantum yield) increasing with the degree of protonation. Reactions with *N*‐halogenosuccinimides or *N*‐halogenophthalimides led to a series of new redox‐active halogeno‐ and succinimido‐/phthalimido‐substituted derivatives. These highly selective reactions are proposed to proceed via the tri‐ or tetracationic state as the intermediate. The derivatives are oxidised reversibly at slightly higher potentials than that of the unsubstituted compounds to dications and further to tri‐ and tetracations. The integration of redox‐active ligands in the transition‐metal complexes shifts the redox potentials to higher values and also allows reversible oxidation in two potentially separated one‐electron steps.

## Graphical abstract

**Redox‐active guanidines**: New redox‐active guanidines, stable in the neutral as well as dicationic redox states and reversibly oxidisable up to the tetracations, were synthesised. Protonation switches on fluorescence; reactions with *N*‐halogenosuccinimides (NXS) and ‐phthalimides selectively gives fully substituted derivatives. Coordination of palladium acetate yields binuclear complexes in which the bridging guanidine ligands are oxidisable in two reversible one‐electron steps.

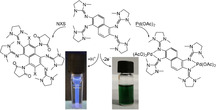

## Introduction

There is a huge demand for redox‐active compounds that exhibit two (or more) stable redox states.^1^ The redox states generally differ in their colour,^2^ and the possibility to interconvert them electrochemically allows their use in electrochromic switching^3^ and memory devices.^4^ Also, applications in organic redox flow batteries depend on the stability of (at least) two redox states.^5^, ^6^, ^7^

**Figure 1:**
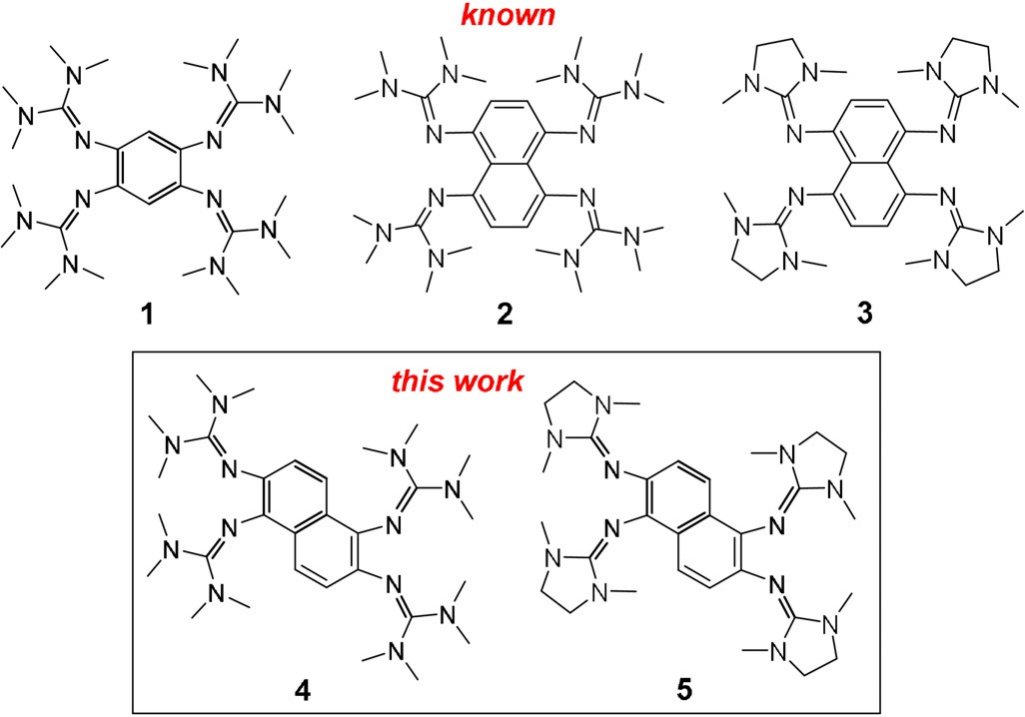
Lewis structures of the known redox‐active guanidines 1,2,4,5‐tetrakis(tetramethylguanidino)‐benzene (**1**), 1,4,5,8‐tetrakis(tetramethylguanidino)‐naphthalene (**2**) and 1,4,5,8‐tetrakis(*N*,*N′*‐dimethylethyleneguanidino)‐naphthalene (**3**), as well as the new guanidines 1,2,5,6‐tetrakis(tetramethylguanidino)‐naphthalene (**4**) and 1,2,5,6‐tetrakis(*N*,*N′*‐dimethylethyleneguanidino)‐naphthalene (**5**) that are isomers of compounds **2** and **3**.

In the last decade, we developed guanidino‐functionalised aromatics (GFAs) as a new class of redox‐active organic molecules,^8^, ^9^ starting with 1,2,4,5‐tetrakis(tetramethylguanidino)‐benzene (**1**, Figure 1).^10^ Compound **1** is a relatively strong organic electron donor with a redox potential *E*_1/2_ (in CH_3_CN) versus ferrocenium/ferrocene (Fc^+^/Fc) of −0.73 V and also a strong Brønsted base with an estimated p*K*_a_ value of 25.3 for (**1**+H)^+^ in CH_3_CN. A number of stable, storable salts of the oxidised compound, for example, **1**(BF_4_)_2_, **1**(PF_6_)_2_, **1**(I_3_)_2_, **1**{N(CN)_2_}_2_ 11 and others, are known. GFA compounds with redox potentials (*E*_1/2_) up to approximately −1 V have been since synthesised.^8^, ^12^ In their neutral state, they were applied in (photochemical) redox reactions^13^ and in their oxidised state in proton‐coupled electron transfer (PCET) reactions.^14^, ^15^, ^16^ Furthermore, they could be employed as redox catalysts for the oxidation of organic substrates with dioxygen as a terminal oxidant.^14^ A variety of mono‐ and bimetallic late‐transition‐metal complexes with redox‐active GFA ligands have been reported, and it is possible to switch by temperature, solvent polarity or by chemical reactions between redox‐isomeric Cu^I^ complexes with oxidised GFA ligands and Cu^II^ complexes with neutral GFA ligands.^17^

The two 1,4,5,8‐tetrakis(guanidino)‐naphthalene derivatives **2** and **3** (see Figure 1)^18^ are slightly weaker electron donors compared to **1** (e.g., *E*_1/2_=−0.65 V vs. Fc^+^/Fc for **2** in CH_2_Cl_2_)^9^ but are double‐proton sponges with very large p*K*_a_ values (estimated to be 27.4 for (**2**+H)^+^ in CH_3_CN). For (**2**+2H)^2+^, the positions of the protons in (asymmetric) N−H⋅⋅⋅N bridges were confirmed by NMR spectroscopic studies in solution and structural characterisation in the solid state.^19^ However, so far only a few stable, storable compounds of the dication **2** ^2+^ are known, for example, the salt **2**(I_3_)_2_ and the complex [**2**(CuBr_2_)_2_] (a dinuclear Cu^I^ complex with the dication **2** ^2+^ as bridging ligand),^20^ hampering the use of the oxidised compound up to date.

In this work, the synthesis and chemistry of the redox‐active guanidines 1,2,5,6‐tetrakis(tetramethylguanidino)‐naphthalene (**4**) and 1,2,5,6‐tetrakis(*N*,*N′*‐dimethylethyleneguanidino)‐naphthalene (**5**) are reported (Figure 1). The properties and chemical reactivity of these new GFA compounds will be shown to differ significantly from those of their isomers **2** and **3**.

## Results and Discussion

### Synthesis and characterisation

**Scheme 1:**
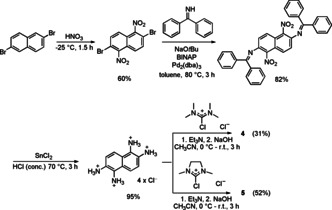
Synthesis of the new redox‐active guanidines **4** and **5** (BINAP=2,2′‐bis(diphenylphosphino)‐1,1′‐binaphthyl; dba=dibenzylideneacetone).

The new tetrakisguanidines **4** and **5** were synthesised in a four‐step procedure according to Scheme 1. The synthesis of the direct precursor 1,2,5,6‐tetraamino‐naphthalene, starting with 2,6‐dibromo‐naphthalene, deviated from the previously reported synthesis^21^ and followed the synthesis reported by Stille et al.^22^ The commercially available 2,6‐dibromo‐naphthalene was nitrated analogous to that reported by Shepherd et al.^23^ by using concentrated HNO_3_ to obtain the required isomer in at least 60 % yield. The substitution of the bromine atoms through amino groups was achieved by using benzophenone imine as an ammonia surrogate in a Buchwald–Hartwig‐type amination, instead of gaseous ammonia in a high‐pressure, high‐temperature reaction, because of better yields and an easier work‐up. Conveniently, the cleavage of the benzophenone imine and the reduction of the nitro groups could be carried out simultaneously by using SnCl_2_ in concentrated hydrochloric acid, giving 1,2,5,6‐tetraaminonaphthalene tetrahydrochloride in 95 % yield. In the last step, this compound was reacted with an “activated urea” compound (from reaction between oxalyl chloride and a urea^24^), either 2‐chloro‐1,1′,3,3′‐tetramethylformamidinium chloride to give **4** (31 % isolated yield) or 2‐chloro‐1,3‐dimethylimidazolinium chloride to give **5** (52 % isolated yield).

**Figure 2:**
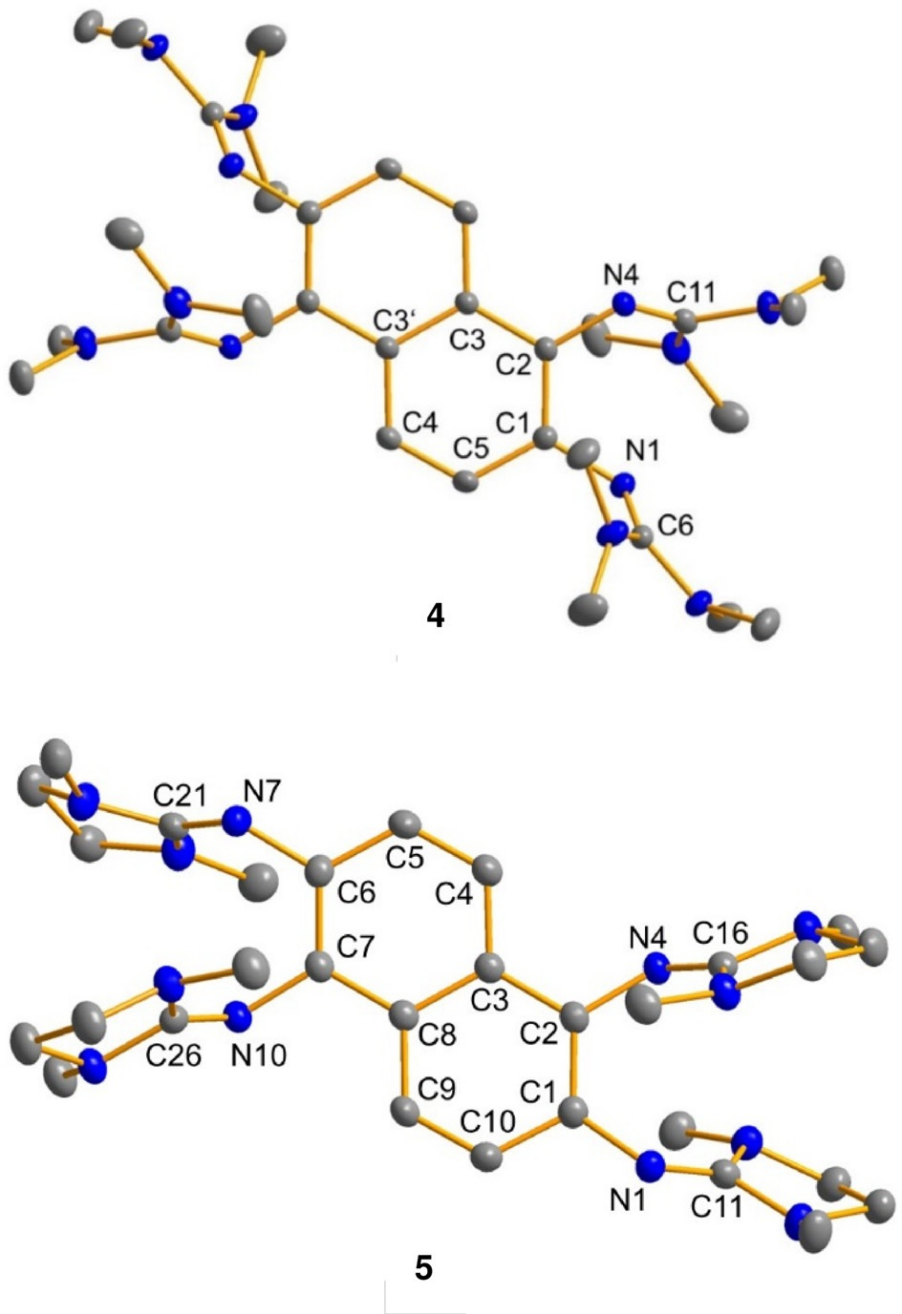
Illustration of the solid‐state structures of compounds **4** and **5**. Displacement ellipsoids are drawn at the 50 % probability level. Hydrogen atoms are omitted. Selected bond parameters can be found in Table 1 (see below).

Crystals of **4** were grown by diffusing diethyl ether into a saturated acetonitrile solution. Crystals of **5** were obtained from a saturated acetonitrile solution. Figure 2 illustrates the solid‐state structures of **4** and **5**. The CN_3_ planes of the guanidino groups are highly twisted (almost perpendicular) with respect to the aromatic‐ring plane, and the C1‐N1‐C6/C11 and C2‐N4‐C11/C16 bond angles at the nitrogen atoms attached to the aromatic core (120.27(11)° and 119.69(11)° for **4** and 127.20(16)° and 122.95(15)° for **5**) display significant deviation from a linear geometry. This “bent‐twisted” structure is in line with the structures of other GFA compounds.^8^, ^25^ The alternative “bent‐planar” structure, in which the guanidino CN_3_ plane is in the aromatic plane, is disfavoured by steric strain. As a consequence of the “bent‐twisted” conformation, there is no steric strain in molecules with two guanidino groups *ortho* to each other (in contrast to two *ortho*‐positioned dimethylamino groups).8a

**Figure 3:**
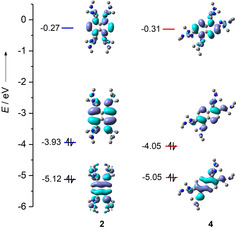
Illustrations of the isodensity surfaces for some frontier orbitals of **2** and **4**.

Quantum chemical calculations using the B3LYP functional in combination with the def2‐TZVP basis set were carried out to compare the relative energy of isomers **2** and **4**. The energy difference was calculated to be 32 kJ mol^−1^ in favour of **4**. Figure 3 compares the isodensity surfaces of the frontier orbitals (HOMO‐1, HOMO and LUMO) of **2** and **4**. For both molecules, the HOMO is localised primarily on the aromatic core. The relatively high HOMO energies (−3.93 eV for **2** and −4.05 eV for **4**) are in line with the observed electron donor character (see below). In the case of the HOMO‐1 orbitals, the isodensity plots exhibit for both molecules one nodal plane but show a different electron‐density distribution. For **2**, the nodal plane intersects the three parallel C−C bond axes of the naphthalene core. In the case of **4**, the nodal plane intersects only the central C−C bond and includes two diagonal C atoms of the naphthalene core. These differences also affect the structures of the dicationic molecules (see discussion below).

### Redox properties

**Figure 4:**
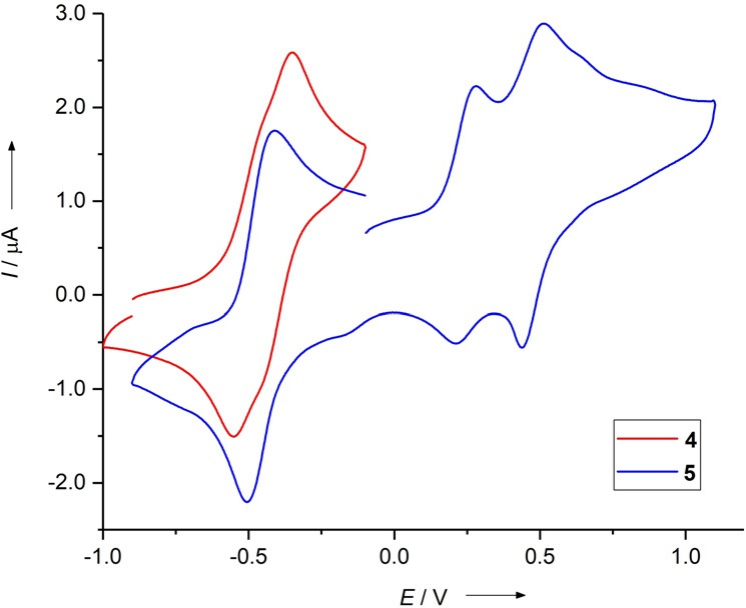
CV curves for **4** and **5** in CH_2_Cl_2_ (Ag/AgCl reference electrode, 0.1 m N(*n*Bu)_4_(PF_6_) as supporting electrolyte, scan rate=100 mV s^−1^). All curves are measured in the direction of oxidation. Potentials are given versus the Fc^+^/Fc couple.

Cyclic voltammetry (CV) measurements of compound **5** (Figure 4) found one quasi‐reversible two‐electron wave at *E*_1/2_=−0.47 V (*E*_ox_=−0.43 V) versus ferrocenium/ferrocene (Fc^+^/Fc) for the redox couple **5**/**5** ^2+^ in CH_2_Cl_2_. For compound **4** the two‐electron oxidation and reduction waves seem to be slightly split, arguing for two one‐electron steps at potentials of *E*_1/2_=−0.51 V (**4**/**4** ^.+^) and *E*_1/2_=−0.40 V (**4** ^.+^/**4** ^2+^). Hence, the new compounds are slightly weaker electron donors than compounds **2** or **3** (*E*_1/2_=−0.65 V vs. Fc^+^/Fc for **2**/**2** ^2+^ and *E*_1/2_=−0.71 V vs. Fc^+^/Fc for **3**/**3** ^2+^).^18^, ^19^ We have previously found a simple correlation between the redox potentials and the HOMO energies of differently substituted GFA compounds, according to which more negative redox potentials directly correlate with higher HOMO energies.^26^ Here again, compound **2** has the more negative redox potential and the higher HOMO energy compared with that of **4** (see Figure 3). By measuring a CV curve for **4** and **5** to higher potentials (see Supporting Information), two more (reversible) one‐electron oxidation steps are visible. These are assigned to the redox couples **4** ^2+^/**4** ^.3+^ (*E*_1/2_=0.27 V, *E*_ox_=0.32 V) and **5** ^2+^/**5** ^.3+^ (*E*_1/2_=0.25 V, *E*_ox_=0.28 V), as well as the redox couples **4** ^.3+^/**4** ^4+^ (*E*_1/2_=0.40 V, *E*_ox_=0.45 V) and **5** ^.3+^/**5** ^4+^ (*E*_1/2_=0.48 V, *E*_ox_=0.51 V). In addition, broad shoulders towards higher potentials of the last oxidation step generating the tetracation are visible. As shown in the next section, the tetracation is barely soluble in standard organic solvents and is extremely reactive. Therefore, these additional features might arise from segregation effects at the electrode and/or decomposition of the highly reactive tri‐ or tetracationic compound. This would also explain the relatively low reduction currents.

The combination of electrochemical oxidation with UV/Vis spectroscopy (spectro‐electrochemistry) was used to obtain information about the oxidised species. These measurements show that the two‐fold oxidised compounds exhibit broad absorptions in the Vis/NIR region, with a maximum of absorption at 778 nm for compound **5** ^2+^ in CH_2_Cl_2_ solution (see Supporting Information). Electronic excitation energies in the visible region signal the presence of an extended π‐conjugated system but removal of aromaticity. For comparison, upon two‐electron oxidation, compound **1** shows strong absorptions near 430 nm. The lower electronic excitation energy of **5** ^2+^ compared with that of **1** ^2+^ could be rationalised by the larger π‐conjugated system in **5** ^2+^.

### Isolation of the dicationic redox state

**Figure 5:**
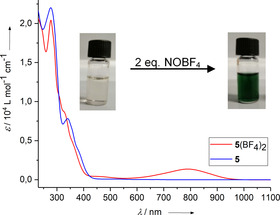
UV/Vis spectra for CH_2_Cl_2_ solutions of **5** and **5**(BF_4_)_2_. Photos of solutions of **5** (left) and **5**(BF_4_)_2_ (right) in CH_2_Cl_2_ are shown in the insets.

Motivated by the reversibility of the redox processes in the CV experiments, we next tried to oxidise compound **5** chemically by reaction with two equivalents of NO(BF_4_). Indeed, neutral **5** was oxidised cleanly to the dication **5** ^2+^, and the product salt **5**(BF_4_)_2_ was isolated in 84 % yield. In Figure 5, the UV/Vis spectra of neutral **5** (reduced form) and **5**(BF_4_)_2_, dissolved in CH_2_Cl_2_, are compared. Oxidation leads to the appearance of a broad absorption in the visible region (*λ*_max_=778 nm) and a small absorption around *λ*_max_=458 nm, in line with the results from spectro‐electrochemical measurements. In combination with strong absorptions centred below 400 nm that extend into the visible region, a deep‐green colour results.

**Figure 6:**
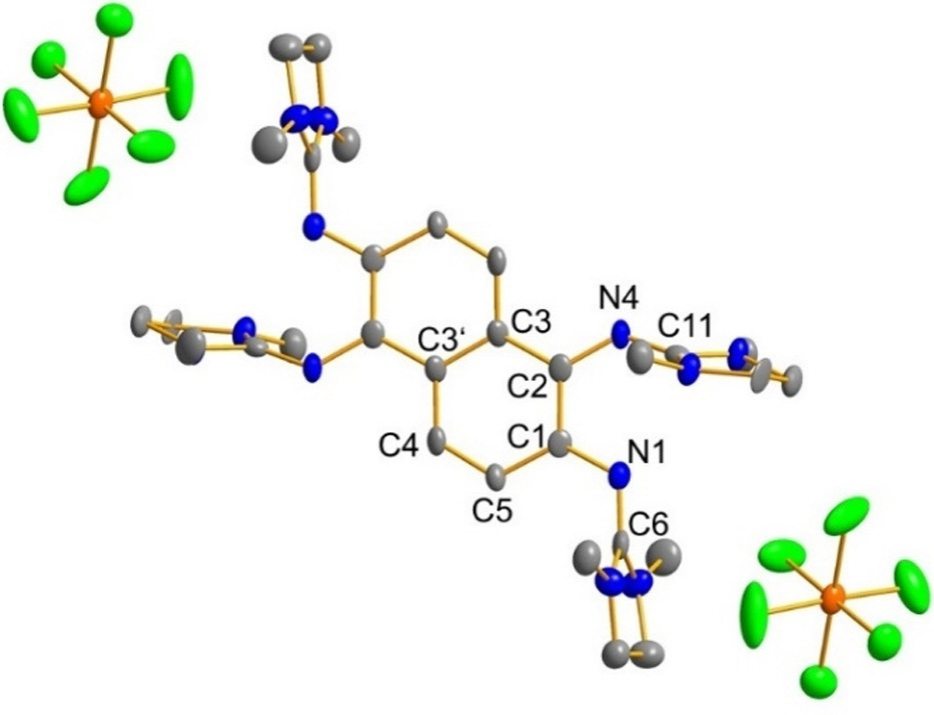
Illustration of the structure of one dication **5** ^2+^ and two SbF_6_^−^ counterions of **5**(SbF_6_)_2_ in the solid state. Displacement ellipsoids are drawn at the 50 % probability level. Hydrogen atoms are omitted. Selected bond parameters can be found in Table 1.

**Scheme 2:**
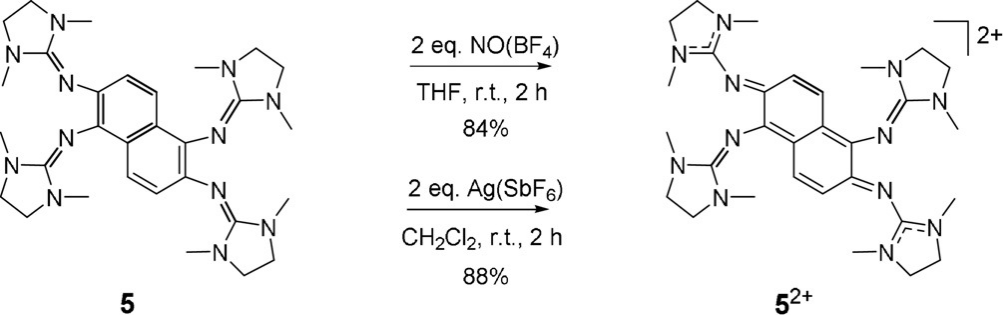
Oxidation of **5** with two equivalents of NO(BF_4_) or Ag(SbF_6_) to form **5** ^2+^.

It was not possible to grow crystals of **5**(BF_4_)_2_ suitable for a structural analysis by single‐crystal X‐ray diffraction (XRD) measurements. Therefore, we repeated the oxidation of **5** with Ag(SbF_6_) and were able to isolate suitable crystals of the product **5**(SbF_6_)_2_ in 88 % yield (Figure 6). In Table 1, some bond lengths of neutral **5** and the dication **5** ^2+^ in solid **5**(SbF_6_)_2_ are compared. The variations in the C−C bond lengths within the central ring system indeed signal loss of aromaticity. Some of the C−C bond lengths are significantly elongated upon oxidation. For example, the C1−C2 bond length increases from 1.396(2) Å before oxidation to 1.475(2) Å after oxidation. The C1−N1 bond length decreases to 1.326(2) Å, and the C2−N4 bond length decreases to 1.355(2) Å. The structure is in line with the Lewis representation drawn in Scheme 2. The “bent‐twisted” conformation of the guanidino groups, which was already found for the neutral molecule, is preserved in the dicationic form; the CN_3_ planes of each guanidino group are highly twisted to the central ring plane, C1‐N1‐C6 and C2‐N4‐C11 bond angles of 119.8(8) and 126.7(9)°, respectively. In this conformation, the N1/N4 atoms could establish a π‐bond with the C1/C2 atoms and at the same time establish some π‐interactions with the C6/C11 atoms.^25^

**Table 1:** Comparison between selected bond lengths [Å] in crystalline neutral **4** and **5**, the salt **5**(SbF_6_)_2_ of the two‐fold oxidised guanidine and the complex [**5**{Pd(OAc)_2_}_2_].

Parameter	**4**	**5**	**5**(SbF_6_)_2_	[**5**{Pd(OAc)_2_}_2_]
C1−C2	1.391(2)	1.396(2)	1.475(2)	1.371(8)
C1−C5/C10	1.419(2)	1.421(2)	1.432(2)	1.400(8)
C2−C3	1.433(2)	1.435(2)	1.410(2)	1.438(7)
C3−C3′/C8	1.429(2)	1.428(2)	1.413(2)	1.440(2)
C3′−C4	1.418(2)	1.423(2)	1.410(2)	1.407(7)
C4−C5	1.374(2)	1.371(3)	1.369(2)	1.367(7)
C1−N1	1.419(2)	1.411(2)	1.326(2)	1.410(7)
C2−N4	1.408(2)	1.418(2)	1.355(2)	1.424(7)

Preparative oxidation of **5** with four equivalents of Ag(SbF_6_) in dichloromethane led to a purple precipitate insoluble in dichloromethane and 1,2‐difluorobenzene. Attempts to dissolve the possible tetracation in acetonitrile, diethyl ether, tetrahydrofuran or dimethylformamide resulted in a green solution indicating reduction to the dication, as confirmed through NMR spectroscopy. Given that all our attempts to dissolve **5**(SbF_6_)_4_ without conversion failed, it was not possible to isolate a pure compound.

### Aromatic substitution

**Scheme 3:**
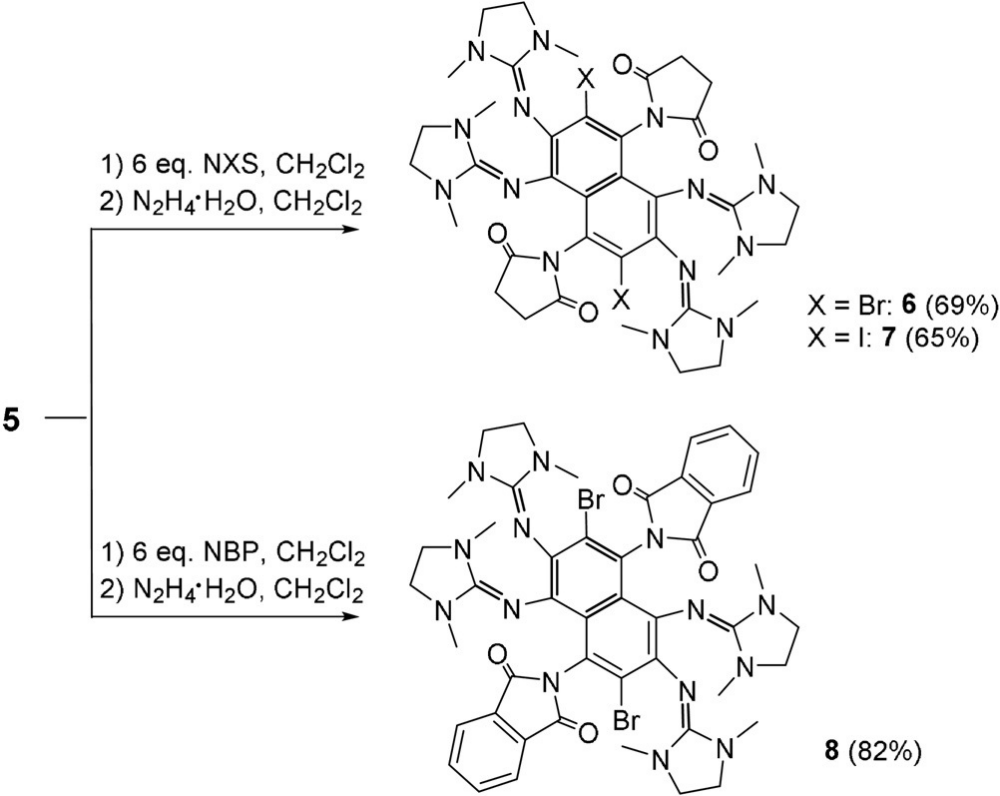
Reaction of **5** with *N*‐halogenosuccinimides (NXS) and *N*‐bromophthalimide (NBP) and subsequent reduction with hydrazine, leading to the new compounds **6**–**8**.

Interestingly, reaction of **5** with *N*‐halogenosuccinimides (NXS) or *N*‐bromophthalimide (NBP) did not only lead to halogeno‐substituted compounds but also to selective substitution by succinimido/phthalimido groups. After reduction with hydrazine, the neutral 1,2,5,6‐tetrakis(dimethylethyleneguanidino)‐3,7‐dihalogeno‐4,8‐disuccinimido‐naphthalenes (**6** and **7**) and 1,2,5,6‐tetrakis(dimethylethyleneguanidino)‐3,7‐dibromo‐4,8‐diphthalimido‐naphthalene (**8**) were obtained in good yields (see Scheme 3), in which all aromatic hydrogen atoms are substituted. Only one of several possible isomers is formed in all reactions. By contrast, reaction of **1** with NXS compounds and subsequent reduction with hydrazine resulted in dihalogenated derivatives of **1**.^9^ The difference can be rationalised by the steric shielding effect of the guanidino groups *ortho* to a C−H proton, allowing substitution only by sterically less‐demanding halogen atoms.

**Figure 7:**
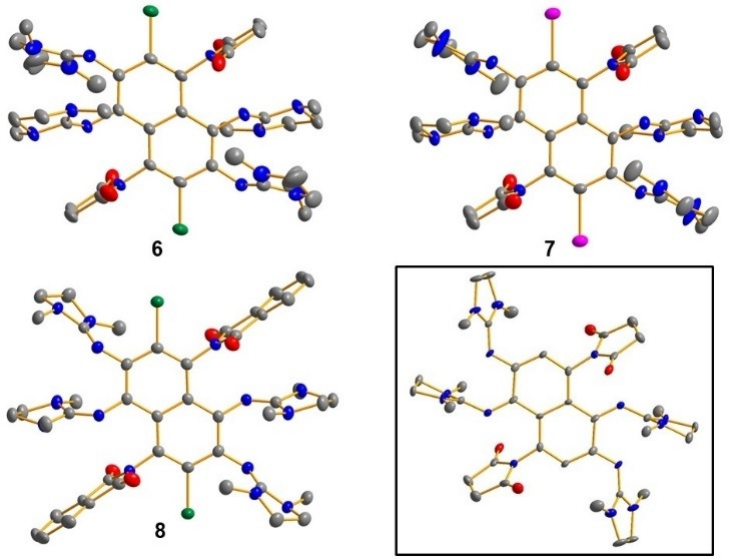
Illustration of the structures of the compounds **6**–**8** and the crystallised product of the reaction of **5** with four equivalents of *N*‐iodosuccinimide (NIS) (i.e., the reaction intermediate) in the solid state. Displacement ellipsoids are drawn at the 50 % probability level for **7**, **8** and the crystallised reaction intermediate and are drawn at the 30 % probability level for **6**. Hydrogen atoms are omitted. Colour code: N=blue, C=grey, O=red, Br=green, I=pink.

Compounds **6**–**8** were crystallised and structurally characterised (Figure 7). Owing to the orientation of the substituents (i.e., all ring planes of the substituents are almost perpendicular to the central naphthalene ring plane), there is no steric constraint in these compounds.

In a preliminary effort to rationalise the pathway of these intriguing reactions, we carried out experiments with different amounts of *N*‐iodosuccinimide. By applying one equivalent, we could only observe an oxidation of **5** to the dication but no substitution of the aromatic hydrogen atoms. On the other hand, the doubly succinimido‐substituted oxidised product (see Figure 7) crystallised (together with two iodide counterions) from a reaction mixture of **5** with four equivalents of *N*‐iodosuccinimide. Hence, the positions 4 and 8 are substituted first, not with a halogen but with succinimide. Using more than six equivalents had no further impact on the reaction. To identify the reactive species, we tried to react **5**(SbF_6_)_2_ with two equivalents of potassium phtalimide but observed no conversion. This result means that phthalimide substitution is possible only after substrate oxidation, given that it does not occur from the neutral form of the substrate. According to these results, we propose a multiple‐step pathway, starting with the oxidation of **5** to either **5** ^.3+^ or **5** ^4+^ by two equivalents of the used reagent, followed by substitution at the positions 4 and 8 by the produced succinimide/phthalimide anion and deprotonation. The resulting disubstituted compound is again oxidised by two more equivalents of the reagent, substituted by the corresponding halides at positions 3 and 7, and deprotonated. Final reduction with hydrazine gives the neutral end‐product. The preference for halide over succinimide/phthalimide substitution in the last substitution steps is presumably due to steric effects.

**Figure 8:**
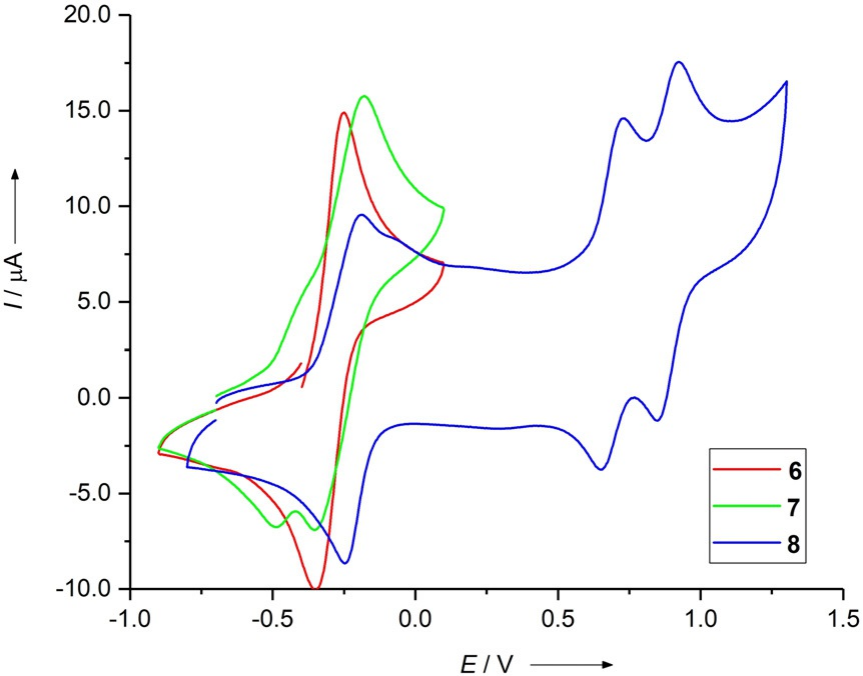
CV curves for **6**–**8** in CH_2_Cl_2_ (Ag/AgCl reference electrode, 0.1 m N(*n*Bu)_4_(PF_6_) as supporting electrolyte, scan rate=100 mV s^−1^). All curves are measured in the direction of oxidation. Potentials are given versus the Fc^+^/Fc couple.

CV measurements in CH_2_Cl_2_ solutions were also carried out for compounds **6**–**8** (Figure 8). As expected, the substitution with electron‐withdrawing groups shifts the redox potential to slightly higher values (*E*_1/2_=−0.30 V (*E*_ox_=−0.25), −0.25 V (*E*_ox_=−0.18) and *E*_ox_=−0.18 V vs. Fc^+^/Fc in CH_2_Cl_2_ for **6**, **8** and **7**, respectively). Similar to the free ligands **4** and **5**, for all compounds two more (reversible) one‐electron oxidation steps were visible at higher potentials (see Supporting Information). These are assigned to the redox couples ^2+^/^.3+^ and ^.3+^/^4+^. For all three compounds, the redox couples ^2+^/^.3+^ as well as the redox couples ^.3+^/^4+^ are (as expected) shifted to higher potentials in comparison to that of **5** (see Table 2).

**Table 2:** *E*_1/2_ and *E*_ox_ values [V vs. Fc^+^/Fc] for compounds **6**–**8** in CH_2_Cl_2_ solution.

Redox couple	**6** *E*_1/2_ (*E*_ox_)	**7** ^[a]^ *E*_1/2_ (*E*_ox_)	**8** *E*_1/2_ (*E*_ox_)
^0^/^2+^	−0.30 (−0.25)	(−0.18)	−0.25 (−0.18)
^2+^/^.3+^	0.67 (0.69)	(0.70)	0.69 (0.73)
^.3+^/^4+^	0.79 (0.85)	(0.84)	0.88 (0.93)

[a] For compound **7**, the reduction wave splits into two components (see Figure 8); thus, no *E*_1/2_ value is given.

### Protonation‐induced fluorescence

The new GFAs **4** and **5** could easily be doubly protonated to give (**4**+2 H)(PF_6_)_2_ and (**5**+2 H)(PF_6_)_2_ by reaction with two equivalents of NH_4_PF_6_. With an excess of HCl**⋅**OEt_2_, they are tetra‐protonated to the salts (**4**+4 H)Cl_4_ and (**5**+4 H)Cl_4_. The bands in the electronic absorption spectra experience small hypsochromic shifts upon protonation. Hypsochromic shifts upon protonation have been reported previously for guanidines.^27^ Recently, such shifts and the influence of intermolecular hydrogen bonding to anions were analysed.^28^

**Figure 9:**
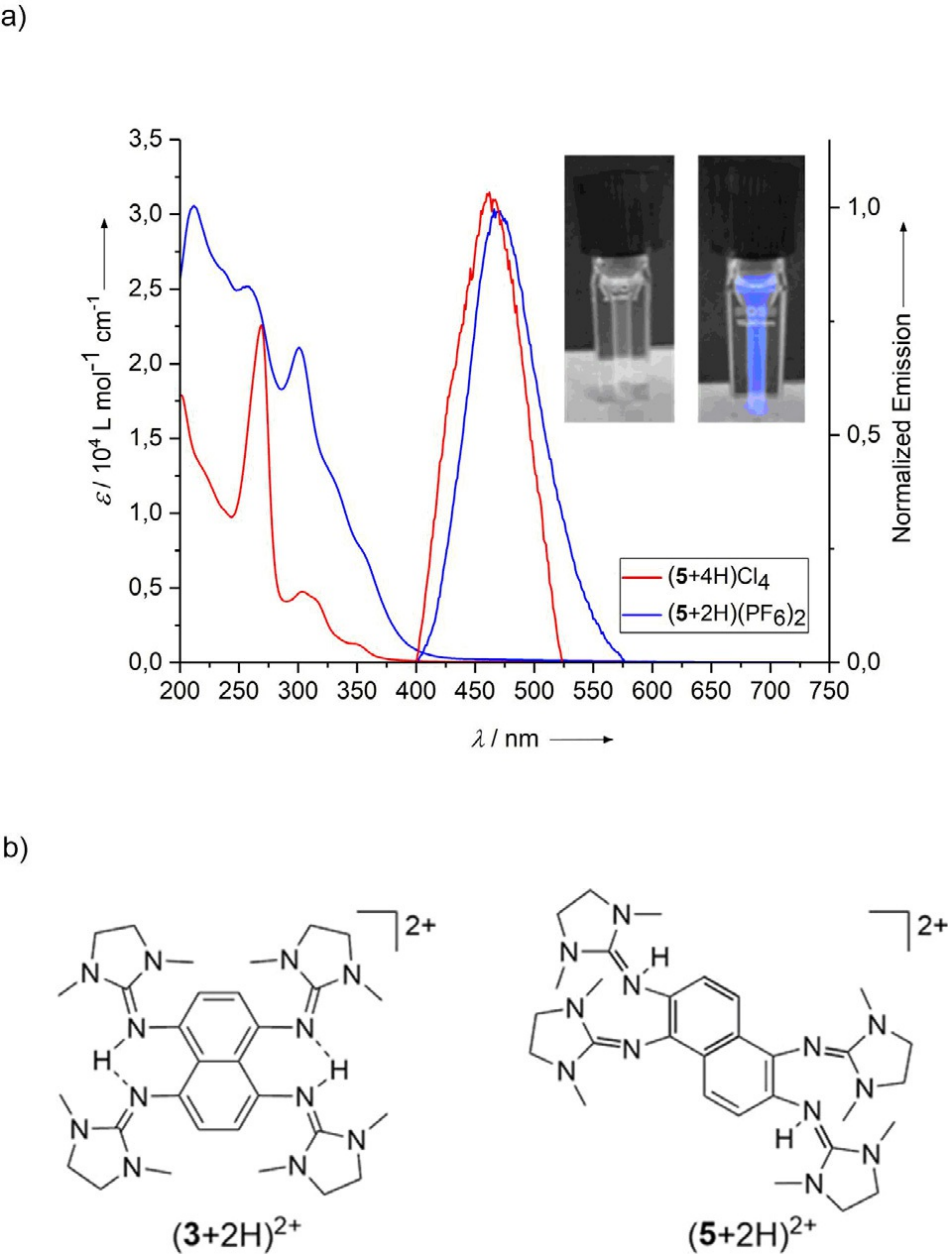
a) UV/Vis and fluorescence spectra for compounds (**5**+2 H)(PF_6_)_2_ and (**5**+4 H)Cl_4_ in CH_3_CN solutions, measured with excitation wavelengths of *λ*_ex_=375 nm for (**5**+4 H)Cl_4_ and *λ*_ex_=385 nm for (**5**+2 H)(PF_6_)_2_. The inset shows photos of the fluorescence of solutions of (**5**+2 H)(PF_6_)_2_ (left) and (**5**+4 H)Cl_4_ (right) with excitation wavelength of 254 nm. b) Comparison between the Lewis structures of (**3**+2H)^2+^ and (**5**+2H)^2+^.

In clear difference to all other GFA compounds, blue fluorescence is switched on by protonation. Although the neutral compounds are fluorescence silent, the protonated compounds show fluorescence signals, with maxima of emission at *λ*=471 nm for (**5**+2 H)(PF_6_)_2_ and *λ*=463 nm for (**5**+4 H)Cl_4_ (Figure 9). The quantum yields for protonated **5** were determined to be approximately 2 % for (**5**+2 H)(PF_6_)_2_ (*λ*_ex_=300 nm) and 15 % for (**5**+4 H)Cl_4_ (*λ*_ex_=280 nm). The drastic increase in the fluorescence intensity with the degree of protonation becomes evident from the photos in Figure 9 a. The increasing energetic separation between electronic ground and excited states might be responsible for a suppression/attenuation of thermal relaxation pathways, explaining the onset of fluorescence upon protonation. Changes in the fluorescence intensity with the degree of protonation (amplification as well as attenuation of fluorescence) have already been observed for guanidines that are fluorescent in their unprotonated form,^29^, ^30^ but such a drastic switching effect from fluorescent silent to fluorescent upon protonation has not been reported previously. In recent years, excited‐state intermolecular proton transfer (ESPT) has been evidenced for some protonated guanidines.^31^, ^32^ However, protonated **5** does not display ESPT, given that changes in the degree of protonation do not lead to shifts in the energy of the fluorescence signal.

In the ^1^H NMR spectrum of (**5**+2 H)(PF_6_)_2_ in CD_3_CN, two of the guanidino methyl groups experience a strong shift relative to **5**, indicating a localised protonation (terminal N−H protons) and the absence of N−H⋅⋅⋅N hydrogen bonds. By contrast, the ^1^H NMR spectrum of (**2**+2 H)(PF_6_) shows a singlet for all methyl protons of the four guanidino groups, indicating delocalised bonding of the protons in N−H⋅⋅⋅N hydrogen bonds, and (**3**+2 H)^2+^ certainly also exhibits this bonding (see Lewis structures in Figure 9 b).^18^ A picture in the Supporting Information (p. 20) illustrates a solid‐state structure that we measured for (**2**+2H)^2+^, also indicating protonation of two guanidino groups with terminal N−H bonds in the solid state. In contrast to the two‐fold protonated forms of **2** and **3**, N−H⋅⋅⋅N hydrogen bonds are absent in the protonated compounds **4** and **5**. Hence, they are not proton sponges.

### Redox‐active coordination compounds

**Scheme 4:**
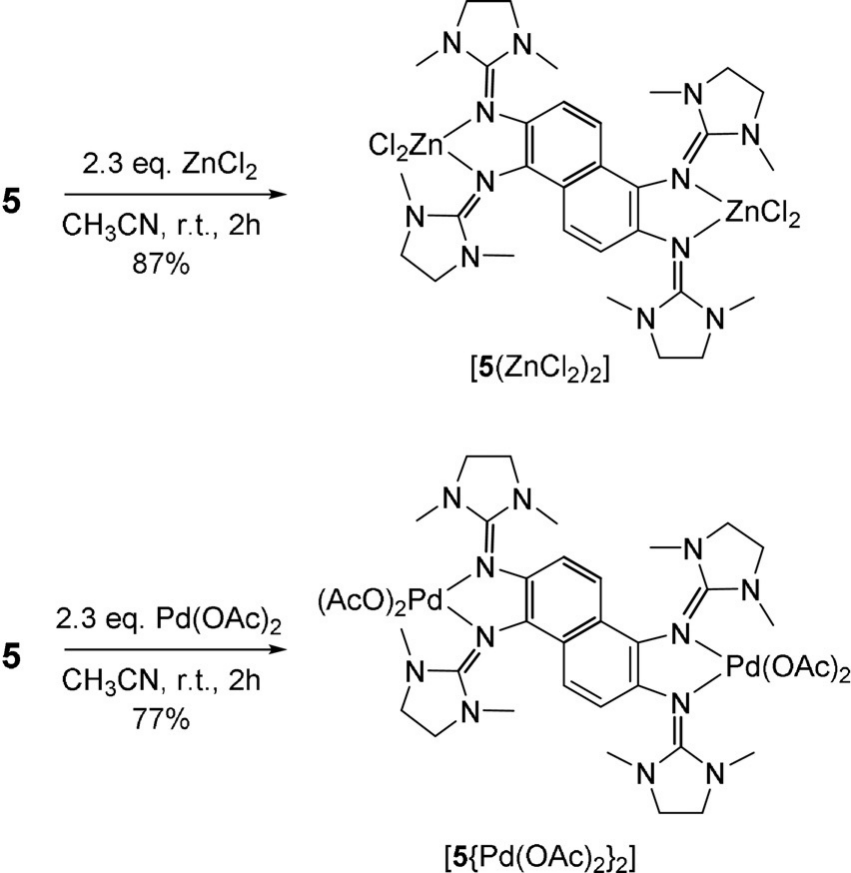
Synthesis of dinuclear metal complexes of redox‐active **5**.

**Figure 10:**
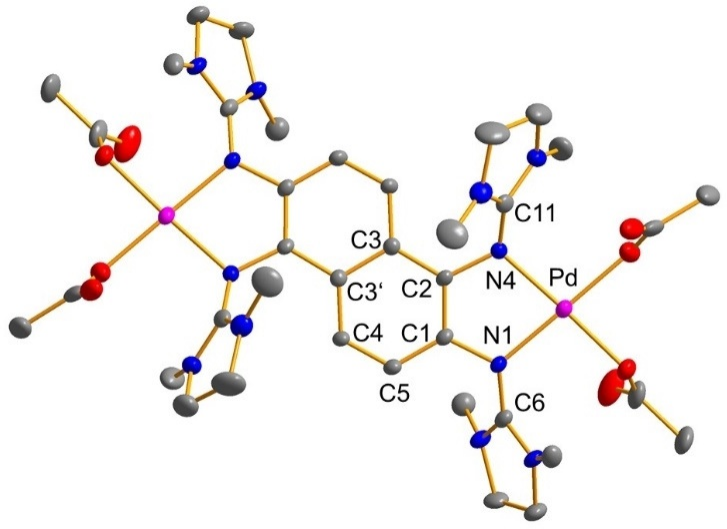
Illustration of the solid‐state structure of [**5**{Pd(OAc)_2_}_2_]. Displacement ellipsoids are drawn at the 30 % probability level. Hydrogen atoms are omitted. Selected bond lengths [Å] and angles [°]: Pd−N1 1.998(5), Pd−N4 2.033(4), N1−C1 1.410(7), N1−C6 1.360(8), N4−C2 1.424(7), N4−C11 1.337(8), C1−C2 1.371(8), C1−C5 1.400(8), C2−C3 1.438(7), C3−C3′ 1.440(10), C3′−C4 1.407(7), C4−C5 1.367(7), N1‐Pd‐N4 82.14(18).

Next, compound **5** was reacted with two equivalents of ZnCl_2_ or Pd(OAc)_2_, leading to the dinuclear complexes [**5**(ZnCl_2_)_2_] and [**5**{Pd(OAc)_2_}_2_] (see Scheme 4). Crystals of [**5**{Pd(OAc)_2_}_2_] were obtained through diffusion of diethyl ether in a saturated dichloromethane solution (see Figure 10). A characteristic feature of binuclear late‐transition‐metal complexes of ligands **2** and **3** is the massive displacement of the metal atom from the aromatic plane of the ligand.^18^, ^19^, ^20^ For example, in the complex [**2**(CoCl_2_)_2_], the cobalt atoms are 1.034 Å above and below this plane.^18^ Similar large displacements were also found for complexes of 1,8‐bis(tetramethylguanidino)naphthalene.^33^ By contrast, the two Pd atoms in [**5**{Pd(OAc)_2_}_2_] remain in the naphthalene ring plane. The differences in the coordination geometries can be rationalised by the different orientations of the lone‐pair imino N orbitals in **2**/**3** and **4**/**5**.

**Figure 11:**
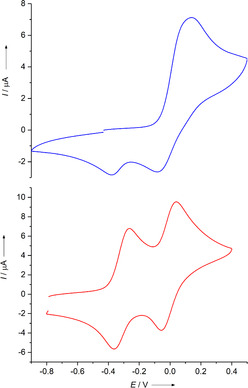
CV curves for [**5**(ZnCl_2_)_2_] (blue) and [**5**{Pd(OAc)_2_}_2_] (red) in CH_2_Cl_2_ solutions (Ag/AgCl reference electrode, 0.1 m N(*n*Bu)_4_(PF_6_) as supporting electrolyte, scan rate 100 mV s^−1^). All curves are measured in the direction of oxidation. Potentials are given versus the Fc^+^/Fc redox couple.

CV measurements show that oxidation of [**5**(ZnCl_2_)_2_] is an irreversible process (Figure 11). Complexation shifts the oxidation potential to higher values (*E*_ox_=0.09 V vs. Fc^+^/Fc for [**5**(ZnCl_2_)_2_] compared to *E*_ox_=−0.43 V for free **5**). As in free **5**, two electrons are removed at equal potential. Interestingly, the reduction seems to occur in two potentially separated waves. Overall, the redox processes are not reversible, indicating some kind of transformation of the oxidised complex (e.g., partial cleavage of the coordinative bond).

Next, the redox properties of [**5**{Pd(OAc)_2_}_2_] were studied in CV measurements (Figure 11). Again, complexation shifts the redox potential to higher values, but now two potentially separated and reversible one‐electron redox events are obtained. These are located at *E*_1/2_=−0.31 V (*E*_ox_=−0.26 V) for the redox couple [**5**{Pd(OAc)_2_}_2_]^.+^/[**5**{Pd(OAc)_2_}_2_] and *E*_1/2_=−0.01 V (*E*_ox_=0.04 V) for the redox couple [**5**{Pd(OAc)_2_}_2_]^2+^/[**5**{Pd(OAc)_2_}_2_]^.+^. By using the formulas in Equations (1), (2):

(1) $$\Delta G{}^{0}=F\times \Delta E{}_{1/2}$$

(2) $$K_{disp}=\exp\left(-\frac{F}{RT}\Delta E_{1/2}\right)$$

(with *E* ^0^≈*E*_1/2_), the Gibbs free energy change (Δ*G* ^0^) for disproportionation of the monocation [**5**{Pd(OAc)_2_}_2_]^.+^ can be estimated to be 29 kJ mol^−1^ and the equilibrium constant *K*_disp_ to be 8.49×10^−6^ (*F*=Faraday constant). Hence, by complexation three stable redox states of **5** (neutral, radical monocationic and dicationic) are assessible. We showed previously that complexation of **1** with metal acetate leads in some cases to two one‐electron waves, allowing the isolation of dinuclear complexes bearing a radical monocationic or dicationic GFA ligand.^34^ Radical monocationic dinuclear copper(II) acetate, nickel(II) acetate or palladium(II) acetate complexes with bridging redox‐active guanidine ligands were shown to exhibit strong ligand–ligand or metal–ligand charge‐transfer bands, and the special role of the acetate co‐ligands in the stabilisation of the radical monocation was evaluated.34a, 34b

Preliminary spectro‐electrochemical measurements for [**5**{Pd(OAc)_2_}_2_] show a clear UV/Vis band around 780 nm upon removal of two electrons, confirming the presence of a dicationic ligand unit. When the oxidation–reduction cycles were repeated several times, the absorption maintained its intensity showing that the ligand‐centred redox process is fully reversible.

## Conclusion

In this work, two new redox‐active organic molecules, 1,2,5,6‐tetrakis(tetramethylguanidino)‐naphthalene (**4**) and 1,2,5,6‐tetrakis(*N*,*N*′‐dimethylethyleneguanidino)‐naphthalene (**5**), were synthesised and their chemistry studied. Two‐electron oxidation of the colourless compounds in CH_2_Cl_2_ solutions at redox potentials *E*_1/2_ of −0.46 V versus ferrocenium/ferrocene (Fc^+^/Fc) for **4**/**4** ^2+^ and −0.47 V for **5**/**5** ^2+^ leads to persistent dark‐green dications, which display a broad electronic transition around 800 nm. Salts of the dication (**5**(BF_4_)_2_ and **5**(SbF_6_)_2_) were synthesised and shown to be stable, storable compounds. Cyclovoltammetric measurements show that further reversible oxidation in two one‐electron steps to the tetracation is possible. The salt **5**(SbF_6_)_4_ appears to be a stable species but an extremely reactive oxidant, which cannot be dissolved in standard organic solvents without being reduced.

The neutral compounds are fluorescence silent. On the other hand, strong blue fluorescence is switched on upon protonation. The quantum yield increases with the degree of protonation, reaching 15 % upon tetraprotonation. This behaviour could be used for fluorescence sensing. We are currently using the protonation‐induced fluorescence to probe the proton‐coupled electron transfer (PCET) reactivity, for example, **5** ^2+^+2 H^+^+2 e^−^→(**5**+2 H)^2+^ (see work on PCET reactivity of other GFA compounds^16^, ^28^).

Aromatic substitution reactions with *N*‐halogenosuccinimides or *N*‐bromophthalimide led in chemo‐selective reactions to the introduction of two halogeno and two succinimido/phthalimido groups, providing convenient access to new fully substituted derivatives with varying redox potentials. A stepwise oxidation–substitution pathway for these highly selective reactions is proposed on the basis of experiments with varying equivalents of the *N*‐iodosuccinimide.

Finally, compound **5** was applied as a redox‐active ligand in binuclear late‐transition‐metal complexes. Palladium acetate coordination does not only shift the redox potentials to higher values, but it also causes the two‐electron redox process for the uncoordinated compound to split into two potentially well‐separated one‐electron steps. Spectro‐electrochemical studies show that the redox processes are ligand‐centred. Hence, for the compound embedded in a metal complex, three stable redox states (neutral, radical monocationic and dicationic) are assessible. The results of this work build the basis for applications of compounds **4** and **5** in coordination chemistry and organic synthesis.

## Experimental Section

### Materials and methods

All reactions were carried out under a dry argon atmosphere using standard Schlenk techniques or in a dinitrogen‐filled glove box (Mbraun LABmaster dp, MB‐20.G). The applied solvents were dried with an MBraun Solvent Purification System and degassed prior to use. 2,6‐Dibromonaphthalene, sodium‐*tert*‐butyloxide, tris(dibenzylideneacetone)palladium(0) [Pd_2_(dba)_3_], tin(II) chloride, 2,2′‐bis(diphenylphosphino)‐1,1′‐binaphthyl (BINAP) and benzophenone imine were purchased from abcr, Sigma–Aldrich or Alfa Aesar and used without further purification. The reagents 2,6‐dibromo‐1,5‐dinitronaphthalene,^35^ 2‐chloro‐1,1′,3,3′‐tetramethylformamidinium chloride^19^ and 2‐chloro‐1,3‐dimethylimidazolinium chloride^19^ were synthesised according to the literature. Infrared spectra were recorded as KBr discs with a BIORAD Excalibur FTS 3000 spectrometer or as solids with an AGILENT Cary 630 FTIR spectrometer. NMR spectra were measured with BRUKER DPX 200, BRUKER Avance II 400 or BRUKER Avance III 600 instruments at a temperature of 298 K if not stated otherwise. Owing to the high sensitivity and low solubility of some of the compounds synthesised, a few ^1^H NMR spectra and most of the ^13^C NMR spectra measured are very dilute. As a result, some of the aromatic ^13^C signals could not be detected and therefore could not be assigned. Elemental analyses were performed at the Microanalytical Laboratory of the University of Heidelberg by using the vario EL and vario MICRO cube devices from Elementar Analysensysteme GmbH. Owing to the high sensitivity of the synthesised compounds to oxygen and water, some elemental analyses deviate by more than 0.4 %. ESI mass spectrometry was performed with a JEOL JMS‐700 magnetic sector and BRUKER ApexQe hybrid 9.4 T FT‐ICR apparatus at the MS Laboratory of the University of Heidelberg. UV/Vis spectra were measured with a VARIAN Cary 5000 UV‐VIS‐NIR spectrometer. CV measurements were carried out with an EG&G Princeton 273 apparatus with an Ag/AgCl reference electrode. All voltammograms were recorded at room temperature. CH_2_Cl_2_ was used as solvent for the individual compounds (*c*=10^−3^ m), and N(*n*Bu)_4_(PF_6_) (electrochemical grade (≥99.0 %), Fluka) was employed as supporting electrolyte (*c*=0.1 m). Spectro‐electrochemical studies were recorded with an AvaSpec 2048×14 CCD spectrometer and a Metrohm Autolab PGSTAT 204 potentiostat/galvanostat in an RHD Instruments TSC spectro sealable cell (working electrode: Pt gauze, counter electrode: Pt disc, Ag as pseudo reference electrode). Fluorescence measurements were recorded with a Varian Cary Eclipse instrument. Quantum yields were estimated with a PTI Quantum Master 40 equipped with an Ulbricht sphere.

### Synthesis

***N***,***N′*** **‐(1,5‐Dinitronaphthalene‐2,6‐diyl)bis(1,1‐diphenylmethanimine)**: Sodium‐*tert*‐butyloxide (961 mg, 10.00 mmol), BINAP (150 mg, 0.24 mmol) and Pd_2_(dba)_3_ (109 mg, 0.12 mmol) were dissolved in degassed toluene (40 mL). The deep‐red solution was stirred at room temperature for 10 min before 2,6‐dibromo‐1,5‐dinitronaphthalene (1.50 g, 4.01 mmol) was added in portions. After stirring for another 5 min, benzophenone imine (1.48 mL, 8.82 mmol) was added dropwise, leading to precipitation of a brown solid. The reaction mixture was stirred for 3 h at 80 °C. Then the solution was allowed to cool down to room temperature, and the solvent was removed under vacuum. The obtained dark‐brown solid was washed with absolute methanol (3×15 mL) and dried under vacuum to yield 1.52 g (2.64 mmol, 82 %) product as a brown powder. C, H, N analysis (%) for C_36_H_24_N_4_O_4_: calcd C 74.99, H 4.20, N 9.72; found C 74.24, H 4.52, N 9.23. ^1^H NMR (600 MHz, CD_2_Cl_2_): *δ*=7.61 (d, ^2^ *J*=8 Hz, 2 H, H_naph_), 7.33–7.56 (m, 20 H, H_benz_), 6.74 ppm (d, ^2^ *J*=12 Hz, 2 H, H_naph_).

**1,2,5,6‐Tetraamino‐naphthalene tetrahydrochloride**: *N*,*N′*‐(1,5‐Dinitronaphthalene‐2,6‐diyl)bis(1,1‐diphenylmethanimine) (1.00 g, 1.74 mmol) and SnCl_2_ (6.60 g, 34.81 mmol) were dissolved in concentrated HCl (30 mL). The red solution was stirred at 70 °C for 3 h. After cooling down the solution to 5 °C, the precipitated red‐brown product was filtered off and washed with degassed EtOH until the washing solution turned colourless. The product was obtained as a white‐grey solid in 95 % yield (530 mg, 1.60 mmol). ^1^H NMR (400 MHz, [D_6_]DMSO): *δ*=7.51 (d, ^2^ *J*=8 Hz, 2 H, H_naph_), 7.24 ppm (d, ^2^ *J*=8 Hz, 2 H, H_naph_).

**1,2,5,6‐Tetrakis(tetramethylguanidino)‐naphthalene (4)**: 1,2,5,6‐Tetraamino‐naphthalene tetrahydrochloride (150 mg, 0.45 mmol) was suspended in degassed acetonitrile (15 mL) at 0 °C. A solution of 2‐chloro‐1,1′,3,3′‐tetramethylformamidinium chloride (338 mg, 1.99 mmol) in acetonitrile (5 mL) was added dropwise to the resulting grey suspension. The reaction mixture was stirred for 10 min at room temperature. Upon addition of triethylamine (1.00 mL, 7.22 mmol), the reaction mixture changed colour to yellow. The solution was stirred for 1 h at 0 °C and an additional 2 h at room temperature. After removing the solvent from the brown solution under vacuum, the residue was redissolved in 25 % NaOH solution and extracted with dichloromethane (3×20 mL). The combined organic phases were dried over potassium carbonate, and the solvent was removed under vacuum. The crude product was recrystallised from acetonitrile to yield 80 mg (31 %, 0.14 mmol) of a colourless solid. Crystals suitable for structural characterisation by single‐crystal X‐ray diffraction were grown by diffusion of diethyl ether into a saturated acetonitrile solution. C, H, N analysis (%) for C_30_H_52_N_12_: calcd C 62.04, H 9.02, N 28.94; found C 61.60, H 8.90, N 28.53. ^1^H NMR (200 MHz, CDCl_3_): *δ*=7.21 (d, ^2^ *J*=5 Hz, 2 H, H_naph_), 6.70 (d, ^2^ *J*=8 Hz, 2 H, H_naph_), 2.64 ppm (d, ^2^ *J*=8 Hz, 48 H, CH_3_). ^1^H NMR (200 MHz, CD_2_Cl_2_): *δ*=7.12 (d, ^2^ *J*=10 Hz, 2 H, H_naph_), 6.54 (d, ^2^ *J*=8 Hz, 2 H, H_naph_), 2.64 ppm (s, 48 H, CH_3_). ^13^C NMR (600 MHz, CD_2_Cl_2_): *δ*=122.60 (s, CH), 116.32 (s, CH), 39.90 (s, CH_3_), 39.49 ppm (s, CH_3_). MS (ESI^+^): *m*/*z* (%)=581.4 (100) [*M*+H]^+^, 536.4 (18) [*M*−NMe_2_]^+^, 491.3 (3) [*M*−(NMe_2_)_2_]^+^. UV/Vis (CH_3_CN): *λ*_max_ (*ϵ* in L mol^−1^ cm^−1^)=257 (2.49×10^4^), 348 nm (0.62×10^4^). IR (KBr): *ṽ*=2924w, 2804w, 1617vs, 1600vs, 1560s, 1371m, 1307w, 1325w, 1136m, 990w, 898w, 613m, 480w cm^−1^.

**1,2,5,6‐Tetrakis(dimethylethyleneguanidino)‐naphthalene (5)**: 1,2,5,6‐Tetraamino‐naphthalene tetrahydrochloride (150 mg, 0.45 mmol) was suspended in degassed acetonitrile (15 mL) at 0 °C. Then 2‐chloro‐1,3‐dimethylimidazolinium chloride (317.5 mg, 1.89 mmol) in acetonitrile (5 mL) was added dropwise to the resulting grey suspension, and the reaction mixture was stirred for 15 min at room temperature. Triethylamine (1.00 mL, 7.22 mmol) was added, resulting in a colour change to red. Subsequently, the solution was stirred for 1 h at 0 °C and for an additional 2 h at room temperature. After removing the solvent from the red solution, the residue was redissolved in 25 % NaOH solution and extracted with dichloromethane (3×20 mL). The combined organic phases were dried over potassium carbonate, and the solvent was removed under vacuum. The crude product was recrystallised from acetonitrile to yield 135 mg (52 %, 0.24 mmol) of a colourless solid. Crystals suitable for an X‐ray analysis were grown from a saturated acetonitrile solution. C, H, N analysis (%) for C_30_H_44_N_12_: calcd C 62.91, H 7.74, N 29.35; found C 62.18, H 7.12, N 29.89. ^1^H NMR (400 MHz, CD_2_Cl_2_): *δ*=7.30 (d, ^2^ *J*=8 Hz, 2 H, H_naph_), 6.81 (d, ^2^ *J*=12 Hz, 2 H, H_naph_), 3.20 (s, 16 H, CH_2_), 2.58 ppm (d, 24 H, CH_3_). ^13^C NMR (600 MHz, CD_2_Cl_2_): *δ*=122.60 (s, CH), 116.56 (s, CH), 49.18 (s, CH_2_), 49.07 (s, CH_2_), 35.23 (s, CH_3_), 34.59 ppm (s, CH_3_). MS (ESI^+^): *m*/*z* (%)=573.4 (100) [*M*+H]^+^. UV/Vis (CH_2_Cl_2_): *λ*_max_ (*ϵ* in L mol^−1^ cm^−1^)=276 (2.19×10^4^), 340 nm (0.56×10^4^). IR (KBr): *ṽ*=2932w, 2842w, 1660vs, 1628vs, 1561s, 1485m, 1394m, 1275m, 1037m, 965w, 910s, 827m, 763w, 712w cm^−1^.

**Compound 5(BF_4_)_2_**: Compound **5** (20 mg, 0.03 mmol) was dissolved in THF (5 mL). Then NO(BF_4_) (7 mg, 0.06 mmol) was added in one portion. The solution was stirred for 2 h at room temperature. During this time, a green solid precipitated. The crude product was filtered off and washed with THF (3×3 mL) and Et_2_O (3×3 mL). The obtained solid was dried under vacuum to yield 22 mg (84 %, 0.03 mmol) of the product as a green solid. C, H, N analysis (%) for C_30_H_44_N_12_B_2_F_8_: calcd C 48.28, H 5.94, N 22.25; found C 48.30, H 6.27, N 22.10. ^1^H NMR (200 MHz, CD_2_Cl_2_): *δ*=7.77 (d, ^2^ *J*=10 Hz, 2 H, H_naph_), 6.00 (d, ^2^ *J*=10 Hz, 2 H, H_naph_), 3.79 (s, 8 H, CH_2_), 3.59 (s, 8 H, CH_2_), 2.80 (s, 12 H, CH_3_), 2.76 ppm (s, 12 H, CH_3_). ^13^C NMR (600 MHz, CD_2_Cl_2_): *δ*=48.73 (s, CH_2_), 48.24 (s, CH_2_), 33.45 (s, CH_2_), 33.30 ppm (s, CH_2_). UV/Vis (CH_2_Cl_2_): *λ*_max_ (*ϵ* in L mol^−1^ cm^−1^)=230 (3.29×10^4^), 277 (3.22×10^4^), 778 nm (0.23×10^4^).

**Compound 5(SbF_6_)_2_**: Compound **5** (20 mg, 0.03 mmol) was dissolved in dichloromethane (5 mL). Then Ag(SbF_6_) (17 mg, 0.06 mmol) was added in one portion. The solution was stirred for 2 h at room temperature. The dark‐green solution was filtered from the precipitated silver, and the solvent was removed under vacuum. The crude product was washed with THF (3×3 mL) and Et_2_O (3×3 mL). The obtained solid was dried under vacuum to yield 32 mg (88 %, 0.03 mmol) of the product as a green solid. Crystals suitable for structural characterisation by single‐crystal X‐ray diffraction were grown by diffusion of pentane into a saturated dichloromethane solution. C, H, N analysis (%) for C_30_H_44_N_12_Sb_2_F_12_: calcd C 34.51, H 4.25, N 16.10; found C 34.89, H 4.52, N 16.13. ^1^H NMR (200 MHz, CD_2_Cl_2_): *δ*=7.77 (d, ^2^ *J*=10 Hz, 2 H, H_naph_), 6.00 (d, ^2^ *J*=10 Hz, 2 H, H_naph_), 3.79 (s, 8 H, CH_2_), 3.59 (s, 8 H, CH_2_), 2.80 (s, 12 H, CH_3_), 2.76 ppm (s, 12 H, CH_3_). ^13^C NMR (600 MHz, CD_2_Cl_2_): *δ*=48.73 (s, CH_2_), 48.24 (s, CH_2_), 33.45 (s, CH_2_), 33.30 ppm (s, CH_2_).

**Compound (5+2 H)(PF_6_)_2_**: Compound **5** (20 mg, 0.03 mmol) was dissolved in tetrahydrofuran (5 mL). Ammonium hexafluorophosphate (13 mg, 0.06 mmol) was added in one portion to the light‐yellow solution. Then the reaction mixture was stirred at room temperature for 1 h. During this time, a white solid precipitated. The crude product was filtered and washed with tetrahydrofuran until the solution turned colourless. Remaining tetrahydrofuran was removed under vacuum to give the product as a white solid in 87 % yield (26 mg, 0.03 mmol). Crystals of (**5**+2 H)(BF_4_)_2_ suitable for structural characterisation by single‐crystal X‐ray diffraction were grown by diffusion of diethyl ether into a saturated solution of **5**(BF_4_)_2_ in dichloromethane. C, H, N analysis (%) for C_30_H_46_N_12_F_12_P_2_ **⋅**THF: calcd C 43.59, H 5.81, N 17.94; found C 43.22, H 6.31, N 17.25. ^1^H NMR (600 MHz, CD_3_CN): *δ*=7.65 (d, ^2^ *J*=12 Hz, 2 H, H_naph_), 7.18 (d, ^2^ *J*=6 Hz, 2 H, H_naph_), 3.54 (s, 8 H, CH_2_), 3.45 (s, 8 H, CH_2_), 2.69 (s, 12 H, CH_3_), 2.58 ppm (s, 12 H, CH_3_). ^13^C NMR (600 MHz, CD_3_CN): 49.44 (s, CH_2_), 49.34 (s, CH_2_), 34.63 (s, CH_3_), 34.32 ppm (s, CH_3_). UV/Vis (CH_3_CN): *λ*_max_ (*ϵ* in L mol^−1^ cm^−1^)=211 (3.05×10^4^), 257 (2.51×10^4^), 301 nm (2.10×10^4^). IR (KBr): *ṽ*=3357m, 2947m, 2882m, 2848m, 1629s, 1604s, 1574m, 1489w, 1443w, 1423w, 1405m, 1390m, 1323w, 1299m, 1287s, 1242m, 1202w, 1142w, 1123w, 1040m, 1021m, 965w, 914m, 879w, 833vs, 787m, 766m, 742w, 712w, 677w cm^−1^.

**Compound (5+4 H)Cl_4_**: Compound **5** (20 mg, 0.03 mmol) was dissolved in tetrahydrofuran (5 mL). HCl (2 m in diethyl ether, 0.1 mL) was added to the light‐yellow solution. After 1 h of stirring at room temperature, a white solid precipitated. The crude product was filtered and washed with tetrahydrofuran (3×5 mL) and diethyl ether (3×5 mL). Then the solid was dried under vacuum to give the product as a colourless solid in 95 % yield (24 mg, 0.03 mmol). The compound is extremely hygroscopic and quickly picks up H_2_O. C, H, N analysis (%) for C_30_H_48_N_12_Cl_4_ **⋅**H_2_O: calcd C 48.92, H 6.84, N 22.28; found C 48.80, H 7.18, N 22.26. ^1^H NMR (200 MHz, CD_3_CN): *δ*=11.95 (s, 2 H, NH), 11.80 (s, 2 H, NH), 8.05 (d, ^2^ *J*=10 Hz, 2 H, H_naph_), 7.65 (d, ^2^ *J*=10 Hz, 2 H, H_naph_), 3.75 (d, ^2^ *J*=12 Hz, 16 H, CH_2_), 2.96 (s, 12 H, CH_3_), 2.78 ppm (s, 12 H, CH_3_). ^13^C NMR spectroscopic shifts are not given because of low solubility. UV/Vis (CH_3_CN): *λ*_max_ (*ϵ* in L mol^−1^ cm^−1^)=268 (2.25×10^4^), 304 nm (0.47×10^4^). IR (KBr): *ṽ*=2930m, 2877m, 2801m, 2660m, 1591vs, 1479m, 1401m, 1373s, 1336w, 1296s, 1127w, 1080w, 1037m, 970m, 913m, 834w, 785w, 686m cm^−1^.

**1,2,5,6‐Tetrakis(*N***,***N′*** **‐dimethylethyleneguanidino)‐3,7‐dibromo‐4,8‐disuccinimido‐naphthalene (6)**: Compound **5** (20 mg, 0.03 mmol) was dissolved in dichloromethane (5 mL). Then *N*‐bromosuccinimide (37 mg, 0.21 mmol) was added in one portion. The colour of the solution immediately changed to deep yellow. After 1 h of stirring at room temperature, the colour changed to green. The solvent was removed under vacuum, and the remaining solid was washed with tetrahydrofuran (2×5 mL). The oxidised product was dissolved in dichloromethane (8 mL), and hydrazine hydrate (80 % in H_2_O, 0.1 mL) was added. Gas development and a colour change to light yellow were observed. Subsequently aqueous NaOH solution (15 %, 4 mL) was added, and the mixture was stirred for an additional 20 min. The organic phase was separated and dried over MgSO_4_. The product was obtained, after solvent removal, as a yellow solid in 69 % yield (22 mg, 0.02 mmol). Crystals suitable for an X‐ray analysis were grown by diffusion of diethyl ether in a saturated dichloromethane solution. C, H, N analysis (%) for C_38_H_48_N_14_Br_2_O_4_: calcd C 49.36, H 5.23, N 21.21; found C 50.01, H 5.72, N 20.81. ^1^H NMR (200 MHz, CD_2_Cl_2_): *δ*=3.15 (m, 16 H, CH_2gua_), 2.76 (s, 8 H, CH_2succ_), 2.59 (s, 12 H, CH_3_), 2.45 ppm (s, 12 H, CH_3_). ^13^C NMR (600 MHz, CD_2_Cl_2_): *δ*=176.73 (s, CO), 49.02 (s, CH_2_), 48.69 (s, CH_2_), 34.75 (s, CH_3_), 33.98 (s, CH_3_), 29.35 ppm (s, CH_2succ_). MS (ESI^+^): *m*/*z* (%)=925 (100) [*M*+H]^+^, 846 (20) [*M*−Br]^+^. UV/Vis (CH_2_Cl_2_): *λ*_max_ (*ϵ* in L mol^−1^ cm^−1^)=230 (3.69×10^4^), 293 (4.81×10^4^), 355 (0.88×10^4^), 420 nm (1.07×10^4^). IR (KBr): *ṽ*=2935m, 2846m, 1715vs, 1648vs, 1633vs, 1536w, 1489m, 1441w, 1420s, 1387s, 1338m, 1280s, 1243s, 1190s, 1114w, 1071w, 1045m, 1028m, 998w, 965w, 933s, 828m, 797m, 765m, 721m, 710m, 705m, 697m cm^−1^.

**1,2,5,6‐Tetrakis(*N***,***N′*** **‐dimethylethyleneguanidino)‐3,7‐diiodo‐4,8‐disuccinimido‐naphthalene (7)**: Compound **5** (20 mg, 0.03 mmol) was dissolved in dichloromethane (5 mL). Then *N*‐iodosuccinimide (47 mg, 0.21 mmol) was added in one portion. The colour of the solution immediately changed to deep yellow. After 2 h of stirring at room temperature, the colour turned green. The solvent was removed under vacuum, and the remaining solid was washed with tetrahydrofuran (2×5 mL). The oxidised product was dissolved in dichloromethane (8 mL), and hydrazine hydrate (80 % in H_2_O, 0.1 mL) was added. Gas development and a colour change to light yellow were detected. Subsequently aqueous NaOH solution (15 %, 4 mL) was added, and the mixture was stirred for an additional 20 min. The organic phase was separated and dried over MgSO_4_. The product was obtained, after solvent removal, as a yellow solid in 65 % yield (24 mg, 0.02 mmol). Crystals suitable for an X‐ray analysis were grown by diffusion of diethyl ether in a saturated dichloromethane solution. C, H, N analysis (%) for C_38_H_48_N_14_I_2_O_4_: calcd C 44.80, H 4.75, N 19.25; found C 44.94, H 5.66, N 19.03. ^1^H NMR (200 MHz, CD_2_Cl_2_) *δ*=3.15 (m, 16 H, CH_2gua_), 2.76 (s, 8 H, CH_2succ_), 2.60 (s, 12 H, CH_3_), 2.44 ppm (s, 12 H, CH_3_). ^13^C NMR (600 MHz, CD_2_Cl_2_): *δ*=176.67 (s, CO), 49.01 (s, CH_2_), 48.68 (s, CH_2_), 34.76 (s, CH_3_), 33.99 (s, CH_3_), 29.53 ppm (s, CH_2succ_). MS (ESI^+^): *m*/*z* (%)=1019 (45) [*M*+H]^+^, 893 (100) [*M*−I]^+^, 767 (25) [*M*−2I]^+^. UV/Vis (CH_2_Cl_2_): *λ*_max_ (*ϵ* in L mol^−1^ cm^−1^)=231 (2.98×10^4^), 295 (3.47×10^4^), 359 (0.62×10^4^), 416 nm (0.69×10^4^). IR (KBr): *ṽ*=3002w, 2927m, 2873m, 2798w, 1721m, 1570s, 1497m, 1495s, 1424m, 1370vs, 1299s, 1234m, 1179s, 1132s, 1064m, 1026m, 1004m, 946w, 918w, 906w, 895w, 850w cm^−1^.

**1,2,5,6‐Tetrakis(*N***,***N′*** **‐dimethylethyleneguanidino)‐3,7‐dibromo‐4,8‐diphthalimide‐naphthalene (8)**: Compound **5** (20 mg, 0.03 mmol) was dissolved in dichloromethane (5 mL). Then *N*‐bromophthalimide (47 mg, 0.21 mmol) was added in one portion. The colour of the solution immediately changed to green. After 2 h of stirring at room temperature, the solvent was removed under vacuum, and the resulting solid was washed with tetrahydrofuran (2×5 mL). The oxidised product was dissolved in dichloromethane (8 mL), and hydrazine hydrate (80 % in H_2_O, 0.1 mL) was added. Gas development and a colour change to red were detected. Then aqueous NaOH solution (15 %, 4 mL) was added to the mixture and stirring continued for 20 min. The organic phase was separated and dried over MgSO_4_. The solvent was removed, giving the product as an orange‐red solid in 82 % yield (29 mg, 0.03 mmol). Crystals suitable for an X‐ray analysis were grown by diffusion of diethyl ether in a saturated dichloromethane solution. C, H, N analysis (%) for C_46_H_48_N_14_Br_2_O_4_: calcd C 54.13, H 4.75, N 19.21; found C 53.52, H 5.03, N 19.28. ^1^H NMR (200 MHz, CD_2_Cl_2_): *δ*=7.89 (m, 4 H, CH_phthalimide_), 7.74 (m, 4 H, CH_phthalimide_), 3.07 (m, 16 H, CH_2_), 2.57 (s, 12 H, CH_3_), 2.16 ppm (s, 12 H, CH_3_). ^13^C NMR (600 MHz, CD_2_Cl_2_): *δ*=167.60 (s, CO), 134.07 (s, C_phthalimide_), 133.87 (s, C_phthalimide_), 123.38 (s, C_phthalimide_), 48.70 (s, CH_2_), 48.11 (s, CH_2_), 34.09 (s, CH_3_), 33.46 ppm (s, CH_3_). UV/Vis (CH_2_Cl_2_): *λ*_max_ (*ϵ* in L mol^−1^ cm^−1^)=234 (4.56×10^4^), 293 (1.77×10^4^), 363 (0.78×10^4^), 410 nm (0.93×10^4^). IR (KBr): *ṽ*=2961w, 2921m, 2842m, 1713s, 1617vs, 1488m, 1440w, 1415m, 1388m, 1347w, 1317w, 1281m, 1253s, 1087m, 1039m, 1021s, 948m, 921w, 883m, 794s, 712vs cm^−1^.

**Compound [5(ZnCl_2_)_2_]**: Compound **5** (20 mg, 0.03 mmol) was dissolved in acetonitrile (5 mL). Then ZnCl_2_ (11 mg, 0.08 mmol) was added. The solution was stirred for 2 h. The precipitated white solid was filtered and washed with Et_2_O (3×6 mL) to remove any remaining ZnCl_2_. The product was dried under vacuum and was obtained as a white solid in 87 % yield (26 mg, 0.03 mmol). C, H, N analysis (%) for C_30_H_44_N_12_Cl_4_Zn_2_: calcd C 42.36, H 5.25, N 19.88; found C 42.40, H 5.57, N 19.88. MS (ESI^+^): *m*/*z* (%)=845 (7) [*M*+H]^+^, 808 (7) [*M*−Cl]^+^, 709 (20) [*M*−ZnCl_2_]^+,^ 573 (100) [*M*−(ZnCl_2_)_2_]^+^. UV/Vis (CH_3_CN): *λ*_max_ (*ϵ* in L mol^−1^ cm^−1^)=234 (0.59×10^4^), 288 (0.95×10^4^), 346 (0.39×10^4^), 398 nm (0.09×10^4^). NMR spectra were not measured because of low solubility.

**Compound [5{Pd(OAc)_2_}_2_]**: Compound **5** (20 mg, 0.03 mmol) was dissolved in acetonitrile (5 mL). Then Pd(OAc)_2_ (17 mg, 0.08 mmol) was added. The solution was stirred, and the colour of the reaction mixture changed to deep red. After 2 h of continuous stirring, the solvent was removed under vacuum, and the black residue was washed with tetrahydrofuran (3×5 mL). The product was dried under vacuum and obtained as a red‐black solid in 77 % yield (27 mg, 0.03 mmol). Crystals suitable for an X‐ray analysis were obtained through diffusion of diethyl ether in a saturated dichloromethane solution. C, H, N analysis (%) for C_38_H_56_N_12_O_8_Pd_2_: calcd C 44.67, H 5.52, N 16.45; found C 43.85, H 5.77, N 17.24. ^1^H NMR (200 MHz, CD_2_Cl_2_): *δ*=6.62 (d, 2 H, CH_naph_), 6.44 (d, 2 H, CH_naph_), 3.50 (s, 16 H, CH_2_), 2.95 (s, 12 H, CH_3_), 2.76 ppm (s, 12 H, CH_3_). ^13^C NMR (600 MHz, CD_2_Cl_2_): *δ*=177.95 (s, CO), 48.43 (s, CH_2_), 47.94 (s, CH_2_), 35.56 (s, CH_3_), 34.46 (s, CH_3_), 23.25 (s, CH_3OAc_), 23.04 ppm (s, CH_3OAc_). UV/Vis (CH_2_Cl_2_): *λ*_max_ (*ϵ* in L mol^−1^ cm^−1^)=231 (3.82×10^4^), 290 (2.44×10^4^), 351 (0.88×10^4^), 404 nm (0.43×10^4^). IR (KBr): *ṽ*=3383m, 2932m, 1660vs, 1536vs, 1467m, 1371vs, 1284vs, 1047w, 1008m, 967m, 935m, 853w, 821m, 792m, 679m cm^−1^.

### Details of the quantum chemical calculations

DFT calculations were carried out with the TURBOMOLE program package.^36^ The B3LYP functional^37^ was used in combination with the def2‐TZVP basis set.^38^ All structures are stationary points on the energy potential surface as confirmed by frequency computations.^39^

### Details of the structural characterisations

Suitable crystals for single‐crystal structure determination were taken directly from the mother liquor, immersed in perfluorinated polyether oil and fixed on a cryo loop. For compound **4**, a full shell of intensity data was collected at low temperature with an Agilent Technologies Supernova‐E CCD diffractometer (Mo_Kα_ radiation, microfocus X‐ray tube, multilayer mirror optics). Detector frames (typically w‐scans, occasionally j‐scans, scan width 0.4°) were integrated by profile fitting.^40^, ^41^ Data were corrected for air and detector absorption and for Lorentz and polarisation effects^41^, ^42^ and scaled essentially by application of appropriate spherical harmonic functions.^41^, ^43^ Absorption by the crystal was treated with a semiempirical multiscan method (as part of the scaling process) and augmented by a spherical correction.^41^, ^44^ An illumination correction was performed as part of the numerical absorption correction.^42^ The structure was solved by ab initio dual space methods involving difference Fourier syntheses (VLD procedure)^44^ and refined by full‐matrix least‐squares methods based on *F* ^2^ against all unique reflections.^45^ All non‐hydrogen atoms were given anisotropic displacement parameters. Hydrogen atoms were input at calculated positions and refined with a riding model.^46^

Full shells of intensity data were collected at low temperature with a Nonius Kappa CCD diffractometer (Mo_Kα_ radiation, sealed X‐ray tube, graphite monochromator, compounds: *N*,*N′*‐(1,5‐dinitronaphthalene‐2,6‐diyl)bis(1,1‐diphenylmethanimine), **5**, **6**, **8** and **9**) and a Bruker D8 Venture, dual source instrument (Mo_Kα_ or Cu_Kα_ radiation, microfocus X‐ray tube, Photon III detector, compounds: **5**(SbF_6_)_2_, (**5**+2 H)(BF_4_), [**5**{Pd(OAc)_2_}_2_] and the intermediate of **7**). Data were processed with the standard Nonius and Bruker (SAINT, APEX3) software packages.^47^ Multiscan absorption correction was applied using the SADABS program.^48^ The structures were solved by intrinsic phasing and refined using the SHELX software package.^45^, ^49^ Graphical handling of the structural data during solution and refinement were performed with OLEX2.^50^ All non‐hydrogen atoms were given anisotropic displacement parameters. Hydrogen atoms bound to carbon were input at calculated positions and refined with a riding model. Hydrogen atoms bound to nitrogen were located in difference Fourier syntheses and refined, either fully or with appropriate distance and/or symmetry. CCDC 1968384, 1968385, 1968386, 1968387, 1968388, 1968389, 1968390, 1968391, 1968392, and 1968393 contain the supplementary crystallographic data for this paper. These data are provided free of charge by The Cambridge Crystallographic Data Centre.

## Conflict of interest

The authors declare no conflict of interest.

## Supporting information

As a service to our authors and readers, this journal provides supporting information supplied by the authors. Such materials are peer reviewed and may be re‐organized for online delivery, but are not copy‐edited or typeset. Technical support issues arising from supporting information (other than missing files) should be addressed to the authors.

Supplementary
