# Supplementary material for: 1,2,5,6‐Tetrakis(guanidino)‐Naphthalenes: Electron Donors, Fluorescent Probes and Redox‐Active Ligands
Source: Chemistry. 2020 Apr 21;26(26):5834–45. doi: 10.1002/chem.201905471 (PMC7318682; doi:10.1002/chem.201905471)
Supplement: Supplementary file 1 — Supplementary [file CHEM-26-5834-s001.pdf]

Chemistry–A European Journal

Supporting Information

1,2,5,6-Tetrakis(guanidino)-Naphthalenes: Electron Donors, Fluorescent Probes and Redox-Active Ligands

Lukas Lohmeyer, Elisabeth Kaifer, Hubert Wadepohl, and Hans-Jörg Himmel*[a]

Content

No.	Titel	Page
1	Analytical data for the precursor compounds	2
2	Analytical data for the Ligands **4** and **5**	5
3	Analytical data for the oxidized compounds **5**$(BF_4)_2$ and **5**$(SbF_6)_2$	13
4	Analytical data for the protonated species (**5**+2H)$(PF_6)_2$ and (**5**+4H)Cl_4	17
5	Analytical data for the complexes [**5**$(ZnCl_2)_2$] and [**5**$\{Pd(OAc)_2\}_2$]	22
6	Analytical data for the compounds **6-8**	28
7	Details of the crystal structure determinations	39

1) Analytical data for the precursor compounds

N,*N*'-(1,5-dinitronaphthalene-2,6-diyl)bis(1,1-diphenylmethanimine)

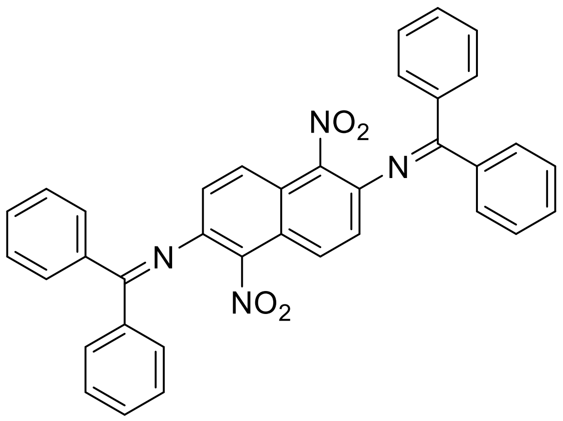

^{1}H NMR spectrum (400 MHz, CD_2Cl_2) of *N*,*N*'-(1,5-dinitronaphthalene-2,6-diyl)bis(1,1-diphenylmethanimine)

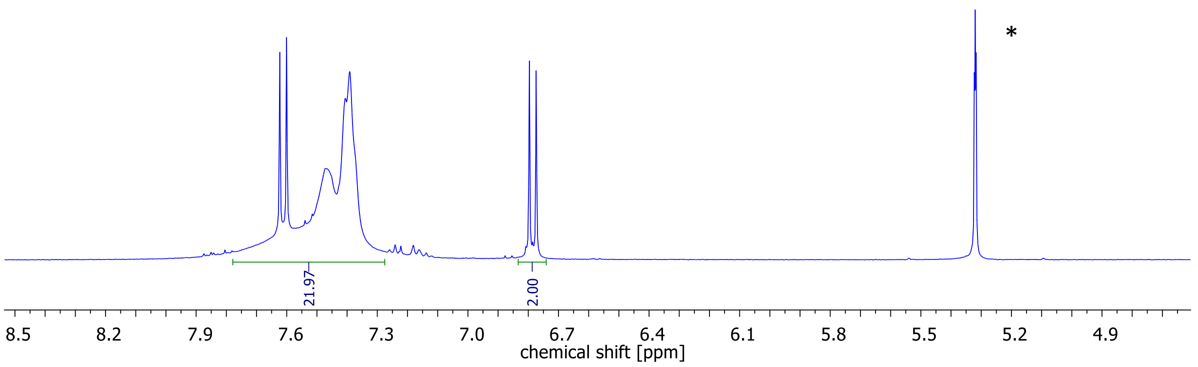

The peak highlighted by an asterisk is due to the solvent.

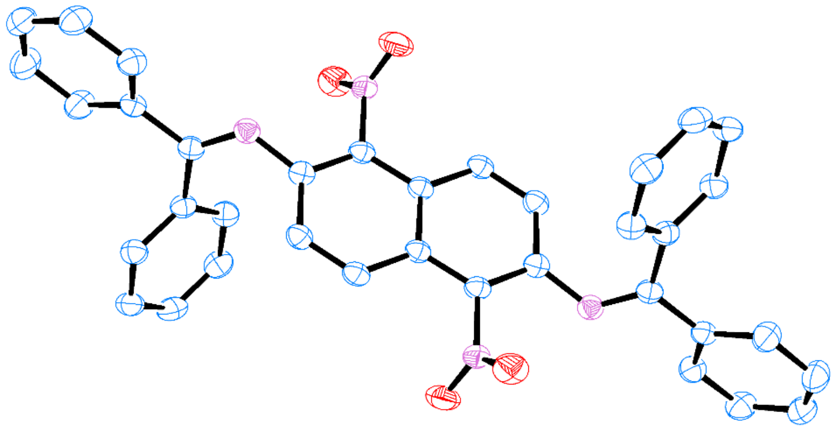

Illustration of the molecular structure of *N*,*N*'-(1,5-dinitronaphthalene-2,6-diyl)bis(1,1-diphenylmethanimine). Hydrogen atoms omitted. Displacement ellipsoids drawn at the 50% probability level.

1,2,5,6-tetraamino-naphthalene-tetrahydrochloride

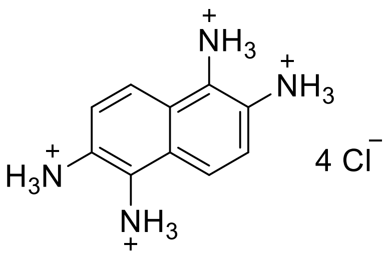

^{1}H NMR-spectrum (400 MHz, DMSO-d6) of 1,2,5,6-tetraamino-naphthalene-tetrahydrochloride

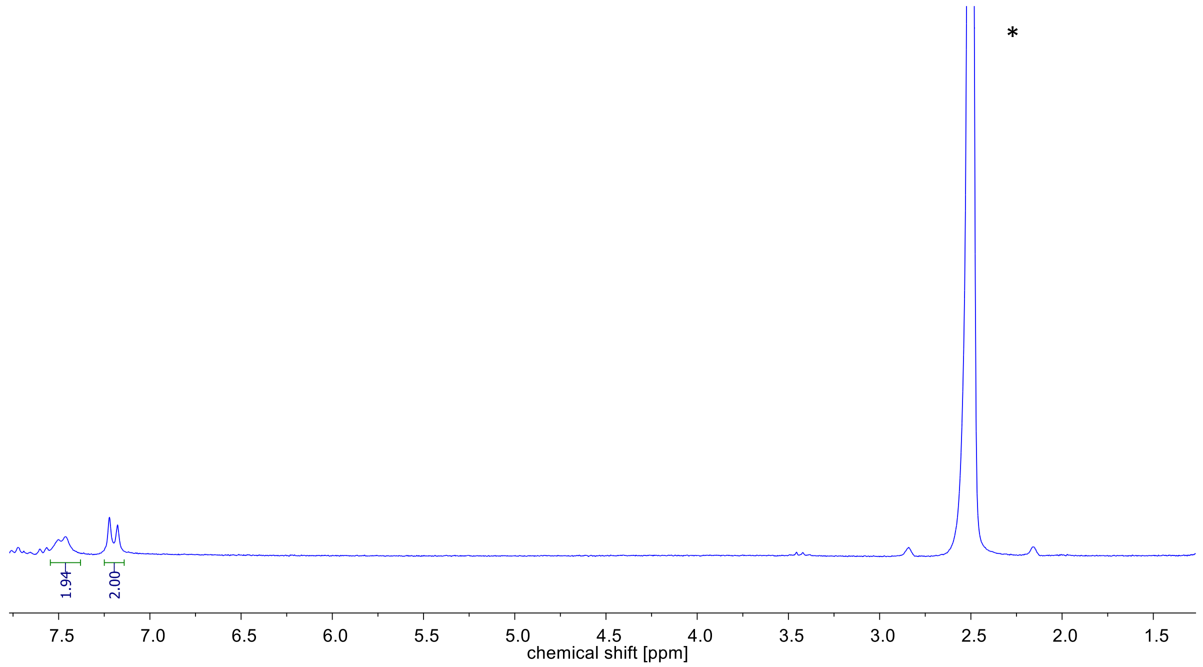

The peak highlighted by an asterisk is due to the solvent.

2) Analytical data for the Ligands 4 and 5

1,2,5,6-tetrakis(tetramethylguanidino)naphthalene (4)

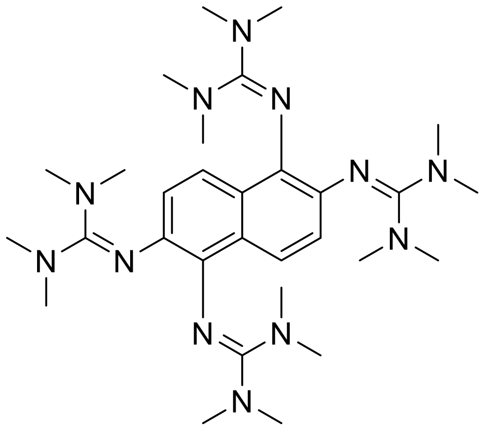

^{1}H NMR spectrum (400 MHz, $CDCl_3$) of compound **4**

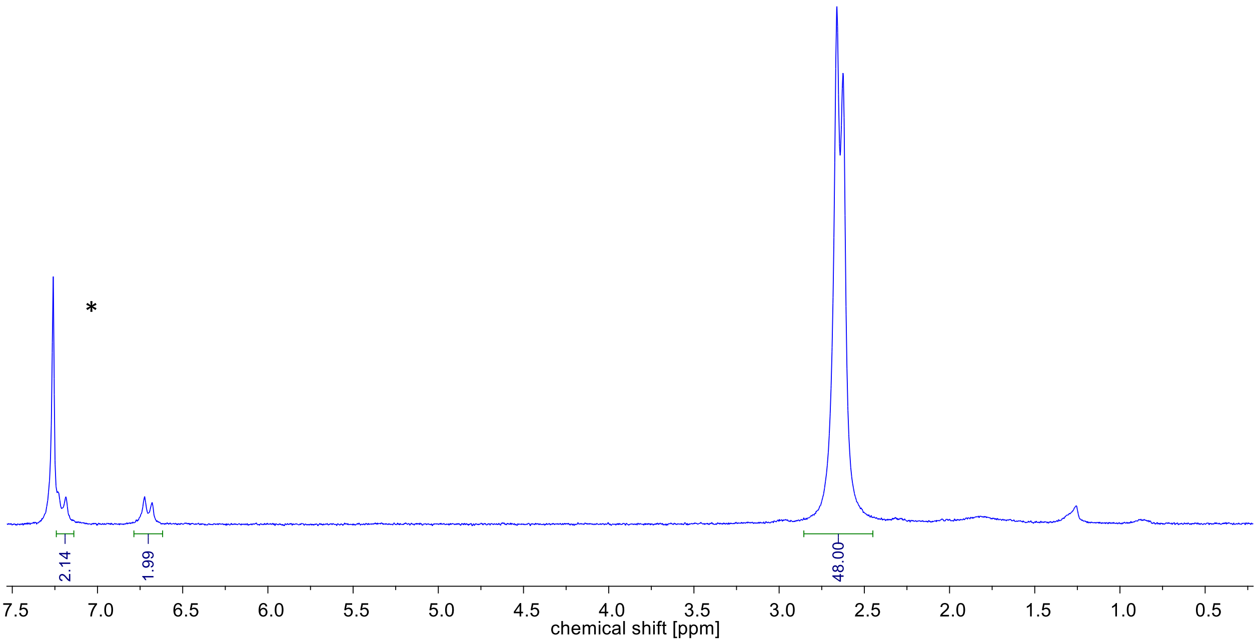

The peak highlighted by an asterisk is due to the solvent.

^{1}H NMR spectrum (400 MHz, CD_3CN) of compound **4**

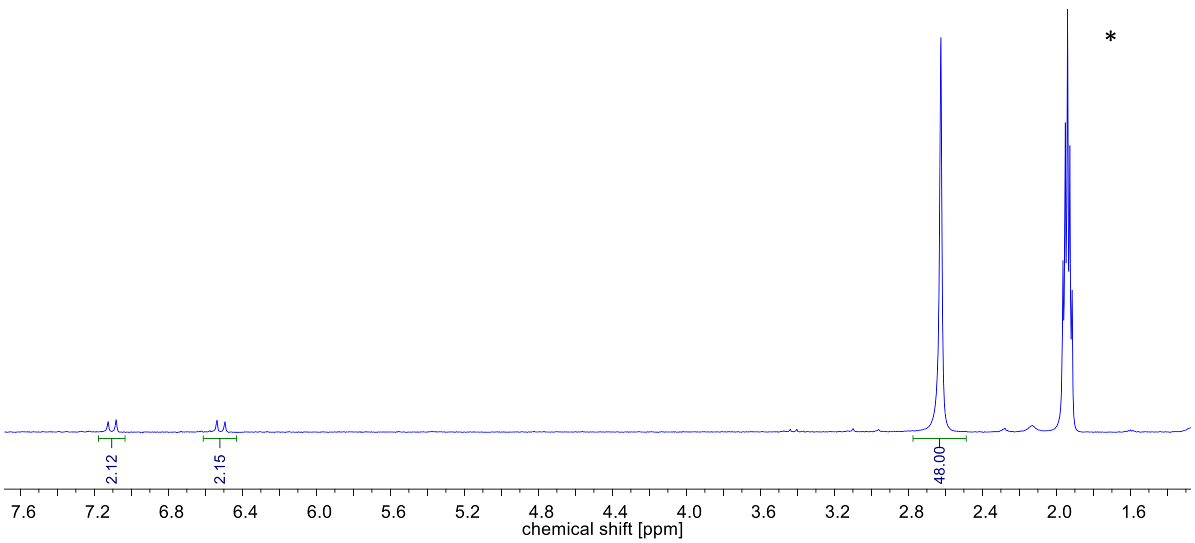

The peak highlighted by an asterisk is due to the solvent.

^{13}C NMR spectrum (600 MHz, CD_2Cl_2) of compound **4**

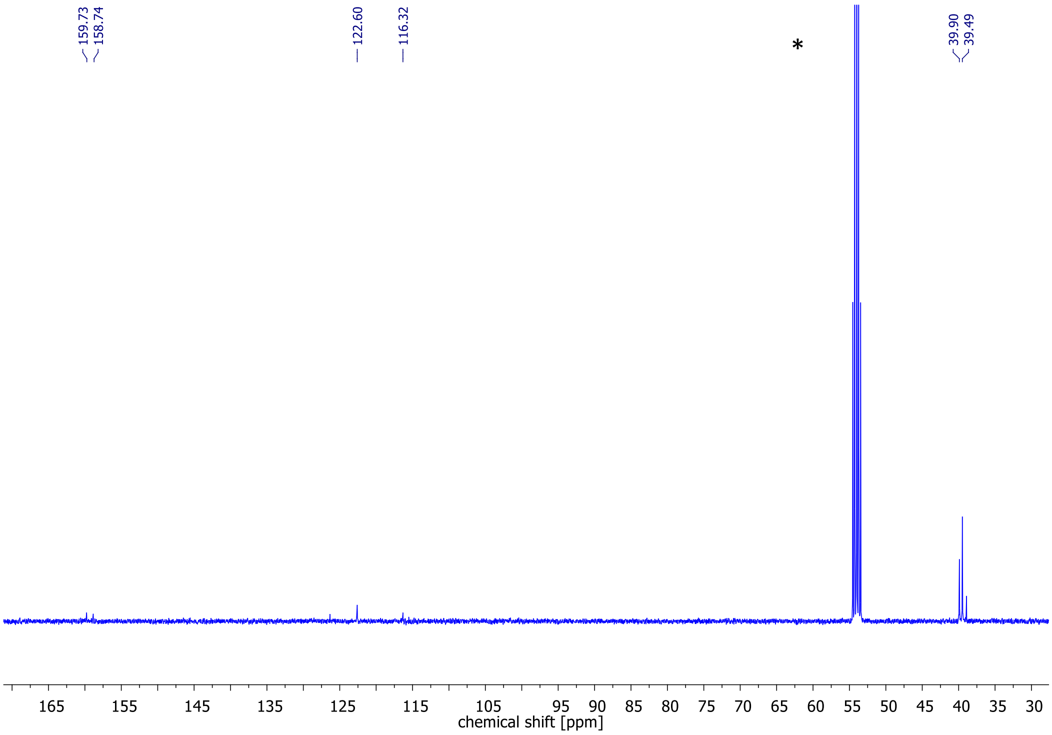

The peak highlighted by an asterisk is due to the solvent.

UV-Vis spectrum (CH_3CN) of compound **4.**

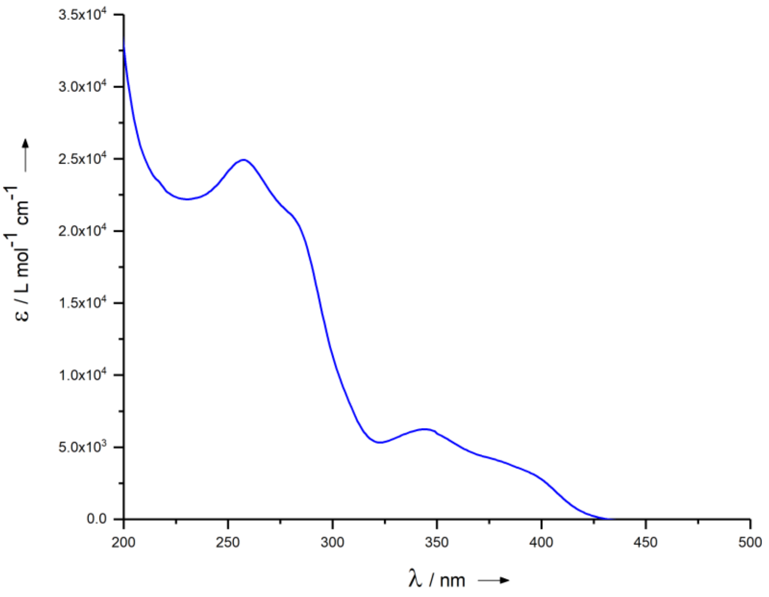

Molecular structure of **4** (hydrogen atoms omitted for clarity, displacement ellipsoids drawn at the 50% probability level).

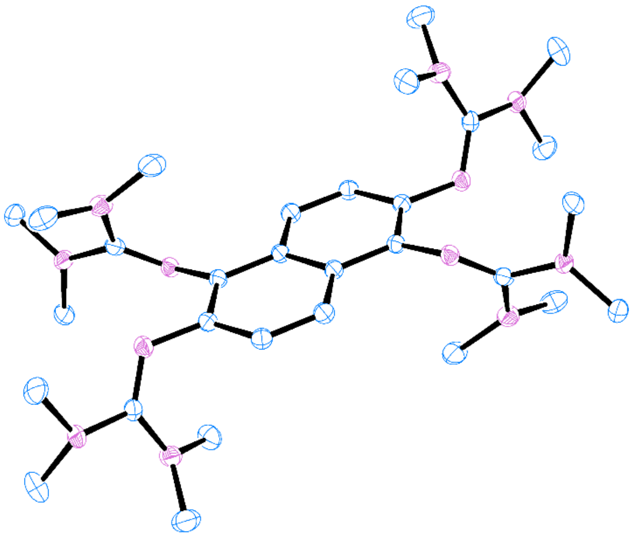

CV curves of compound **4** in CH_3CN (Ag/AgCl reference electrode, 0.1 M N(*n*Bu)$_4$(PF_6) as supporting electrolyte, scan rate 100 mV s^{-1}). Potentials given vs. the Fc^+/Fc redox couple.

First two-electron redox process at $E_{1/2}$ = -0.46 V

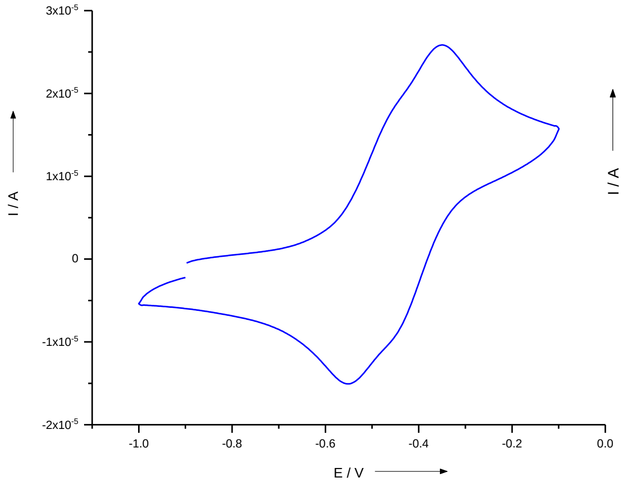

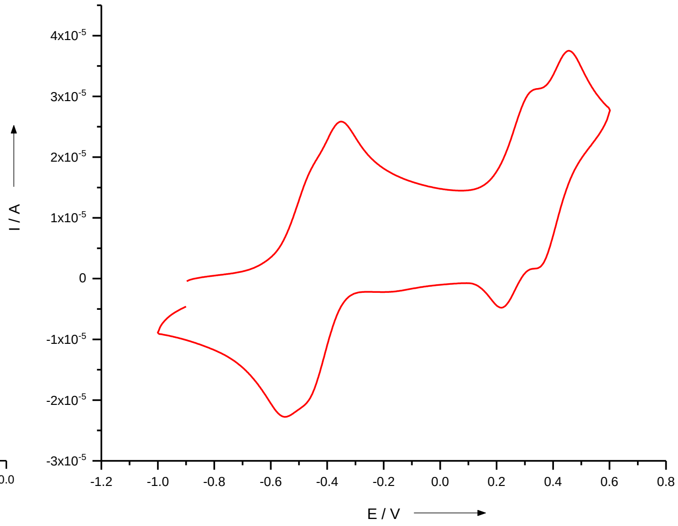

1,2,5,6-tetrakis(dimethylethylene-guanidino)naphthalene (5)

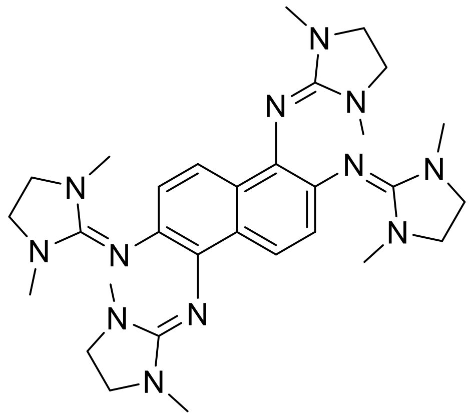

^{1}H NMR spectrum (400 MHz, CD_2Cl_2) of compound **5**

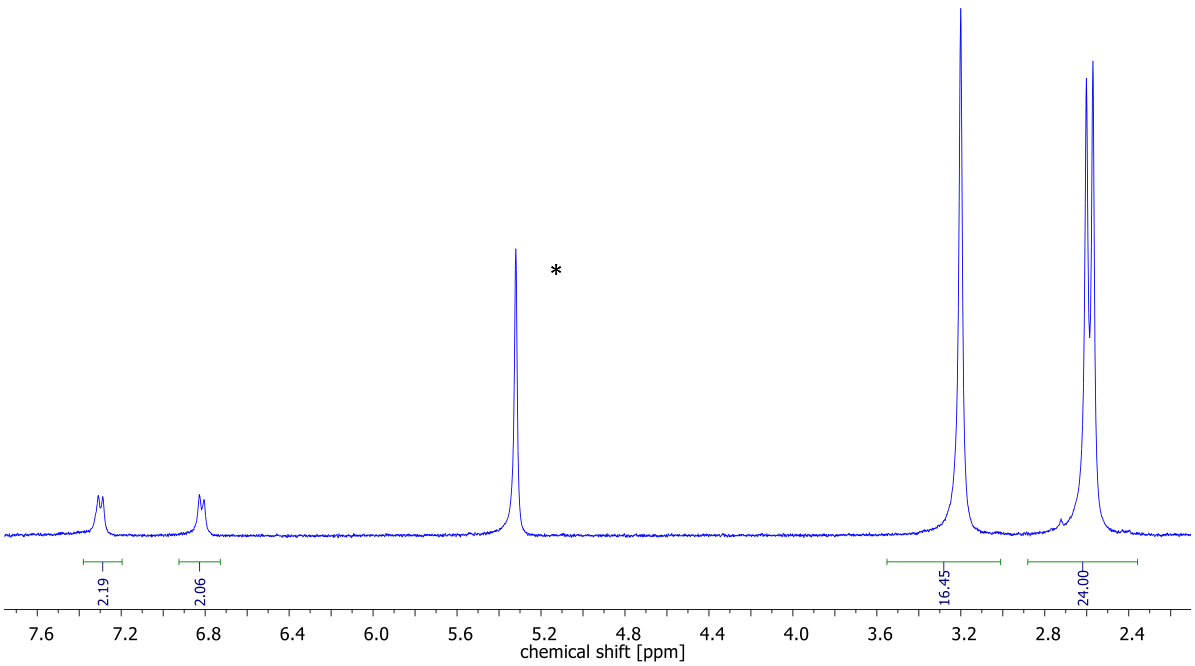

The peak highlighted by an asterisk is due to the solvent.

^{13}C NMR spectrum (600 MHz, CD_2Cl_2) of compound **5**

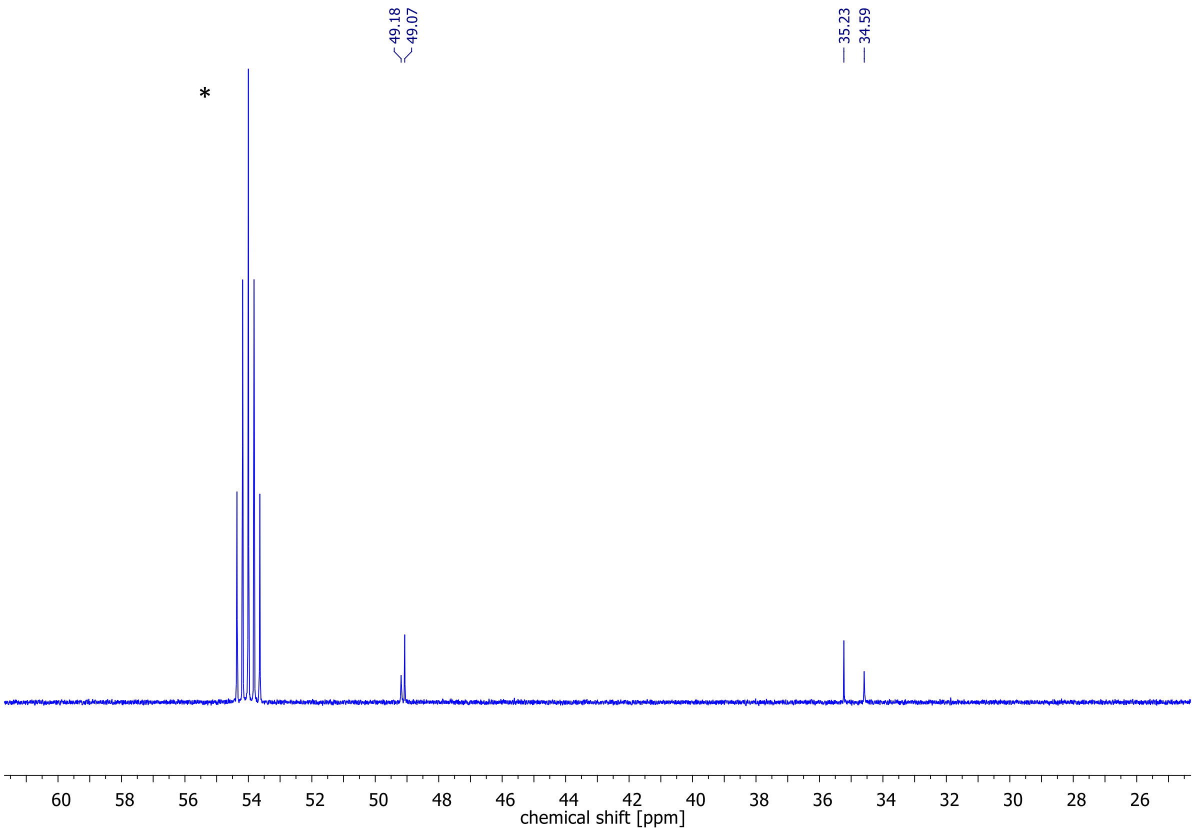

The peak highlighted by an asterisk is due to the solvent.

UV-Vis spectrum (CH_2Cl_2) of compound **5**.

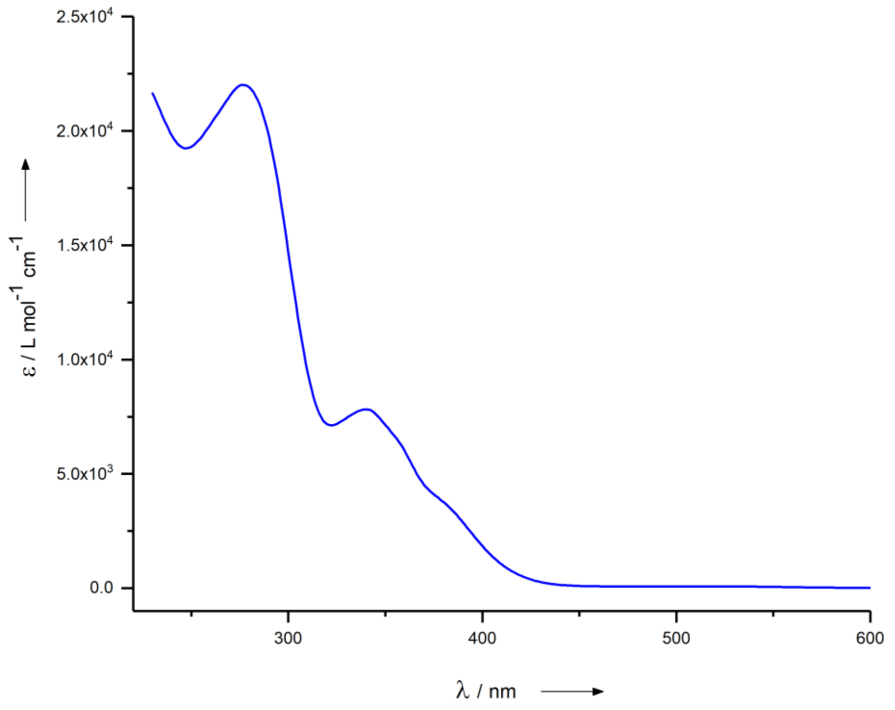

Illustration of the molecular structure of **5** (hydrogen atoms and co-crystallized water omitted for clarity, displacement ellipsoids drawn at the 50% probability level).

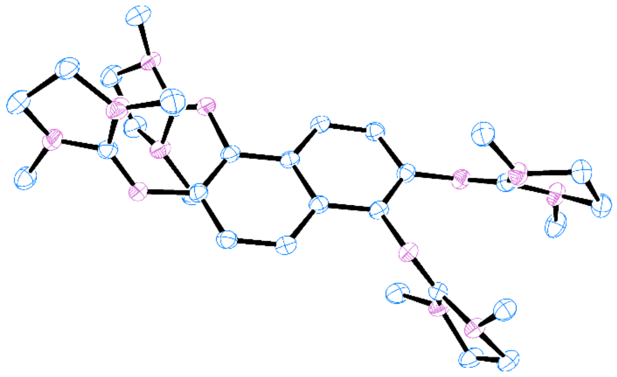

CV curves of compound **5 in** CH_2Cl_2(Ag/AgCl reference electrode, 0.1 M N(*n*Bu)$_4$(PF_6) as supporting electrolyte, scan rate 100 mV s^{-1}). Potentials given vs. the Fc^+/Fc redox couple.

First two-electron redox process at $E_{1/2}$ = -0.47 V

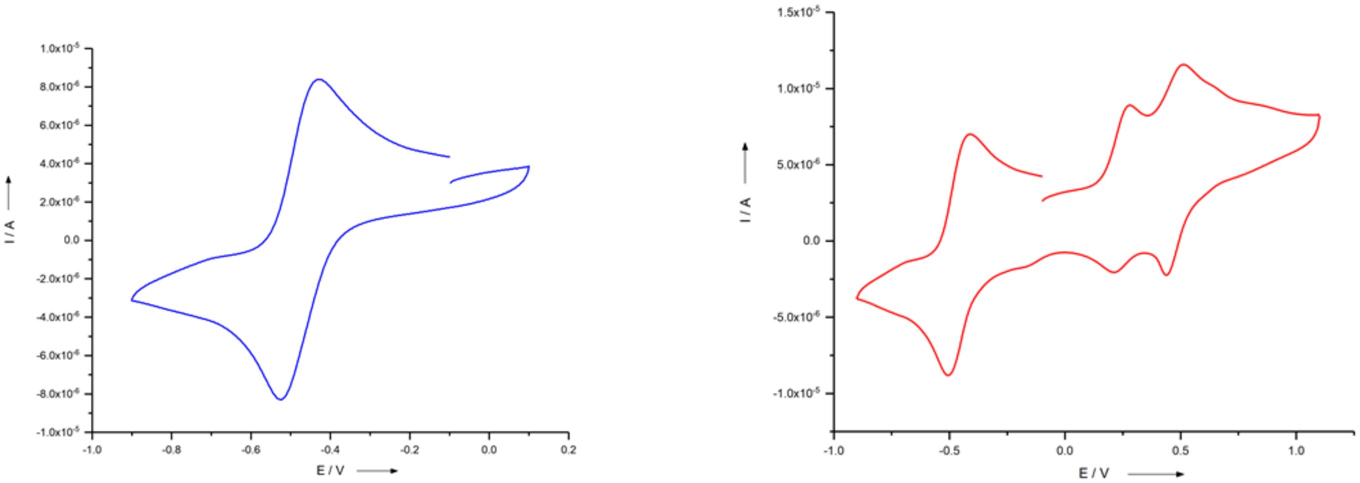

UV-VIS coupled CV measurement of **5** in CH_2Cl_2 (Ag/AgCl reference electrode, 0.5 M N(*n*Bu)$_4$(PF_6) as supporting electrolyte, scan speed 10 mV s^{-1}). 3D-Plot (left) and heatmap (right) of four redox cycles.

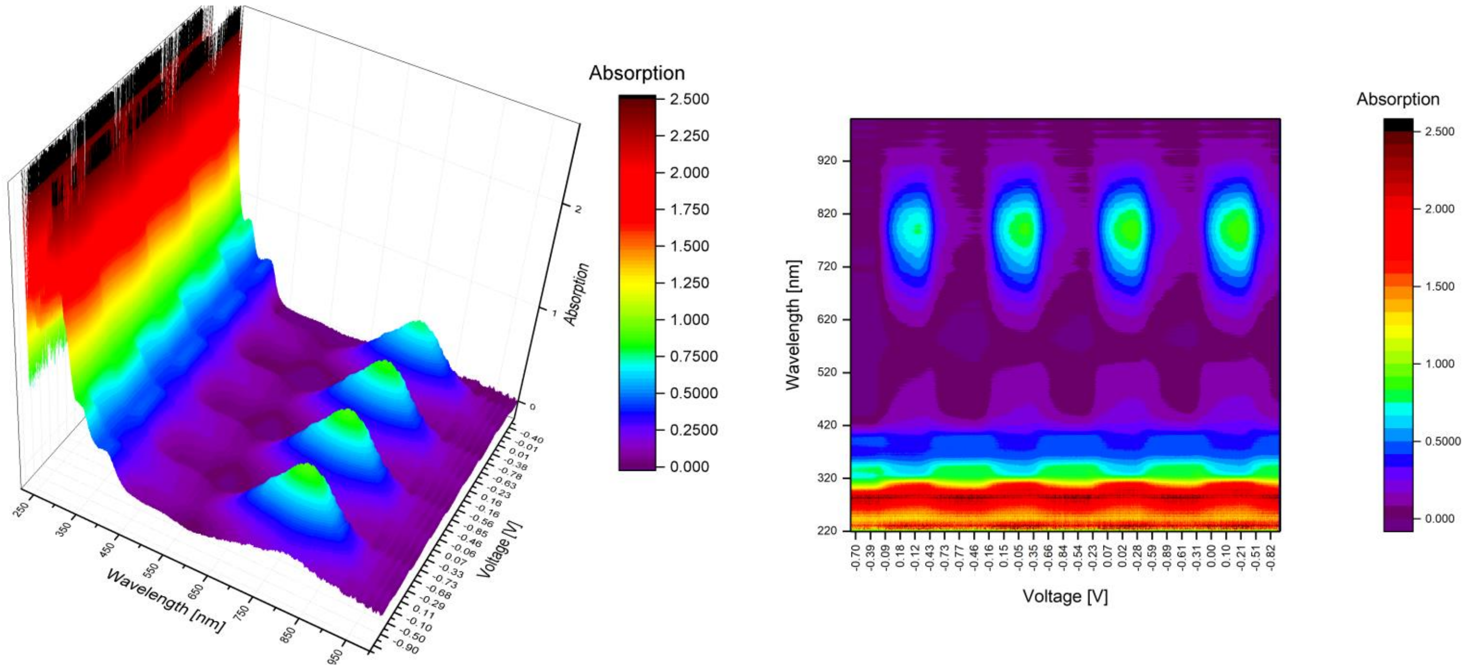

3) Analytical data for the oxidized compounds 5(BF_4)$_2$ and 5(SbF_6)$_2$

5(BF_4)$_2$

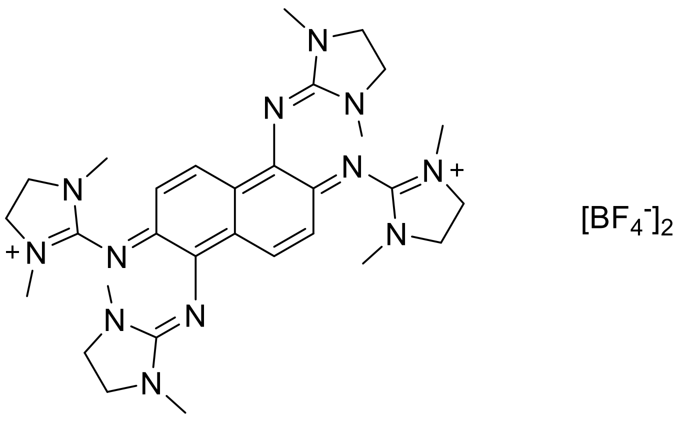

^{1}H NMR spectrum (400 MHz, CD_2Cl_2) of **5**(BF_4)$_2$

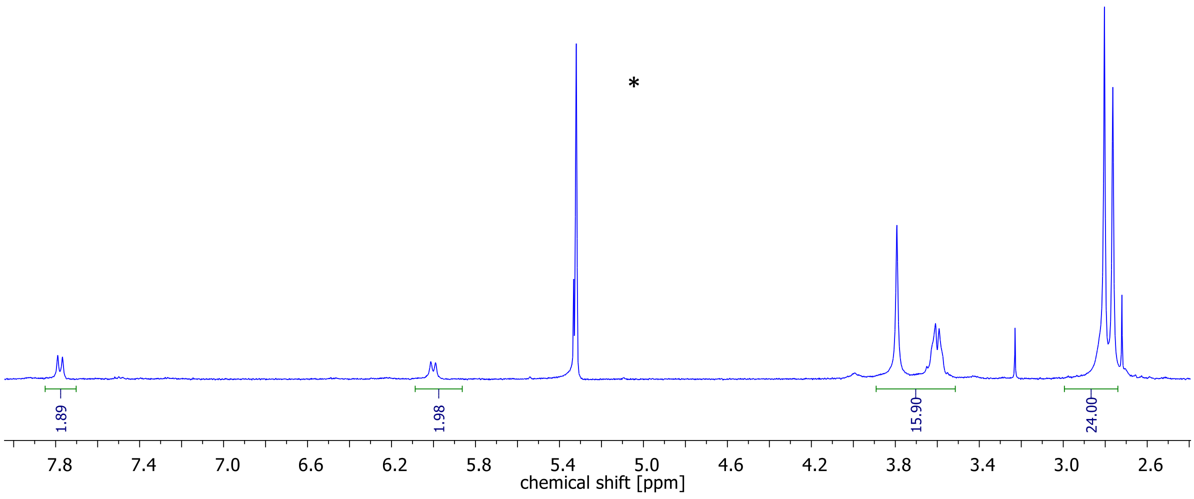

The peak highlighted by an asterisk is due to the solvent.

^{13}C NMR spectrum (600 MHz, CD_2Cl_2) of **5**$(BF_4)_2$

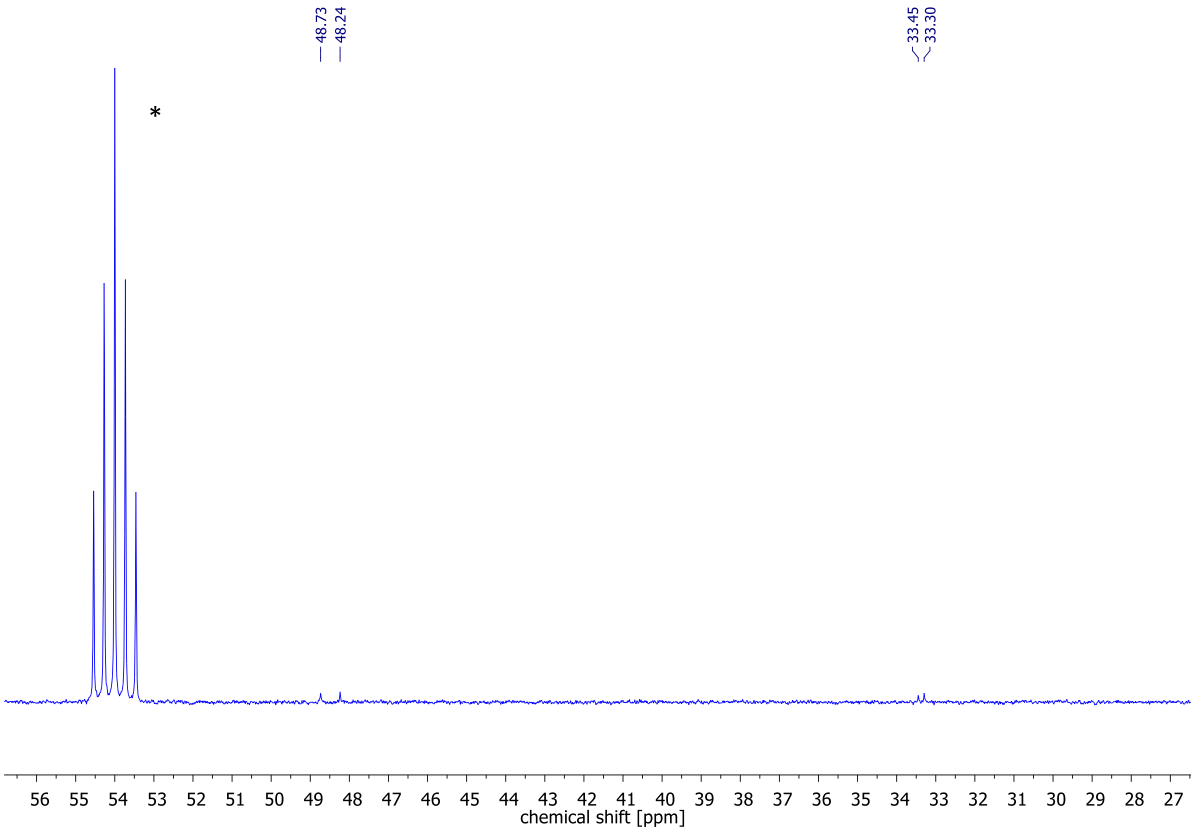

The peak highlighted by an asterisk is due to the solvent.

5$(SbF_6)_2$

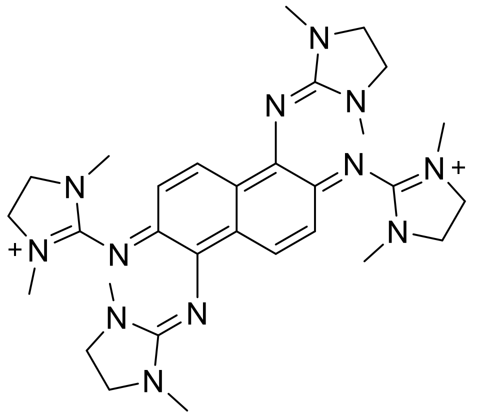

$[SbF_6]_2$

^{1}H NMR spectrum (200 MHz, CD_2Cl_2) of **5**$(SbF_6)_2$

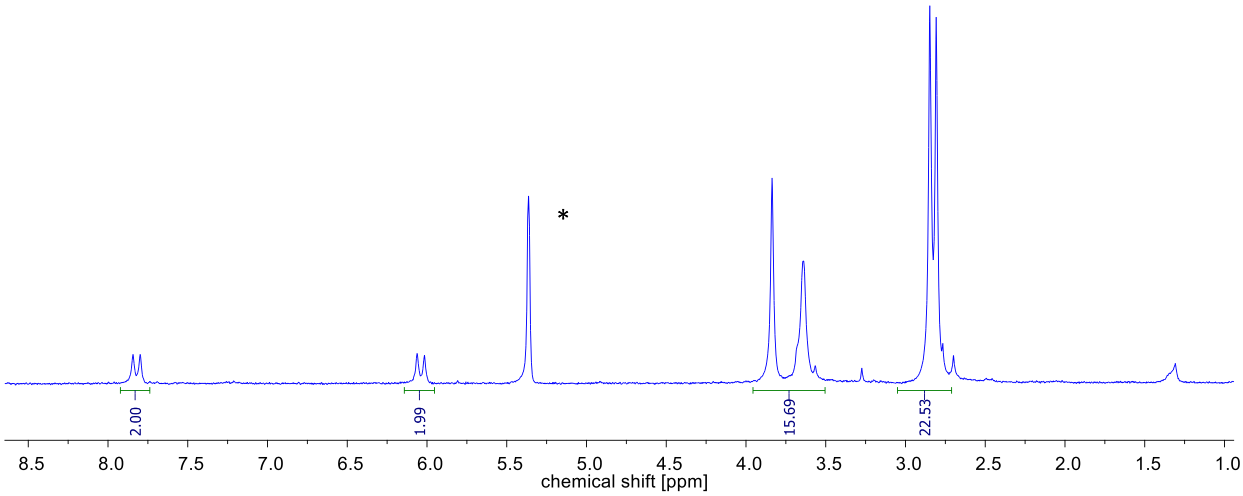

The peak highlighted by an asterisk is due to the solvent.

^{13}C NMR spectrum (600 MHz, CD_2Cl_2) of **5**$(SbF_6)_2$

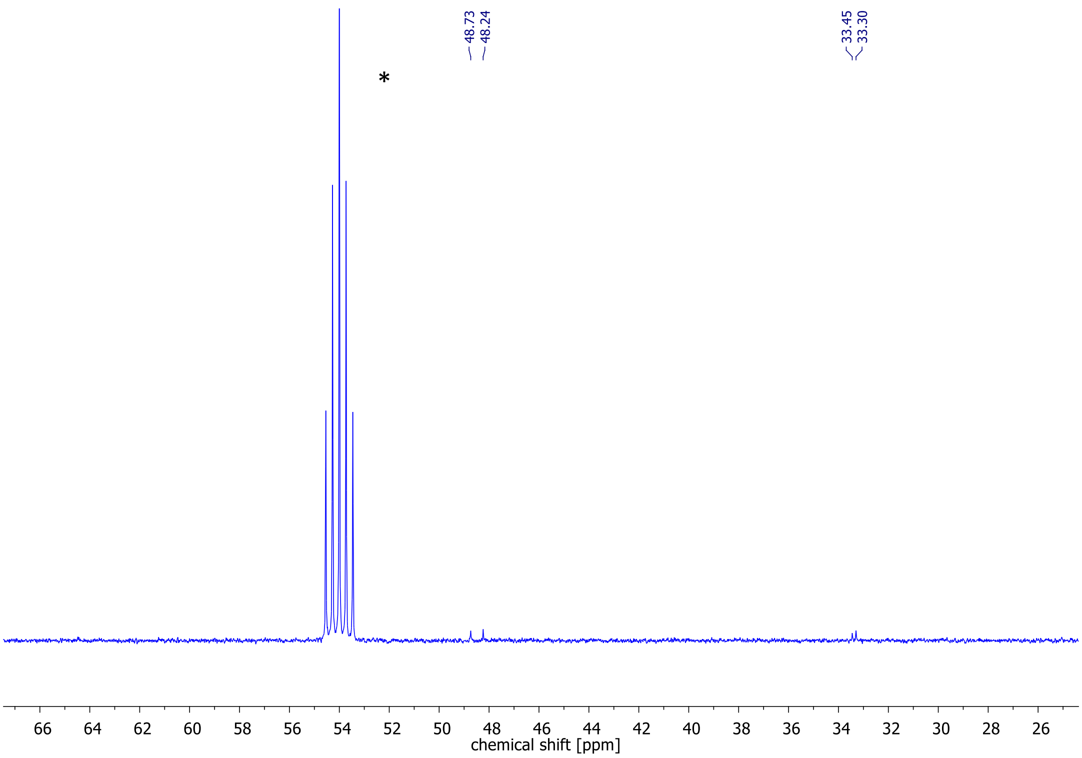

The peak highlighted by an asterisk is due to the solvent.

Molecular structure of **5**$[SbF_6]_2$ (hydrogen atoms omitted for clarity, displacement ellipsoids drawn at the 50% probability level).

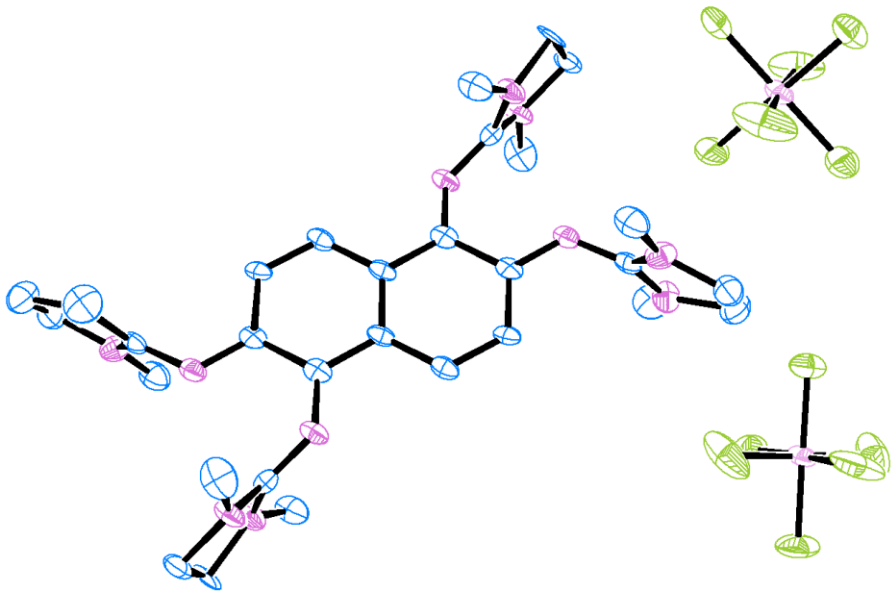

4) Analytical data for the protonated species (5+2H)(PF_6)$_2$ and (5+4H)Cl_4

(**5**+2H)(PF_6)$_2$

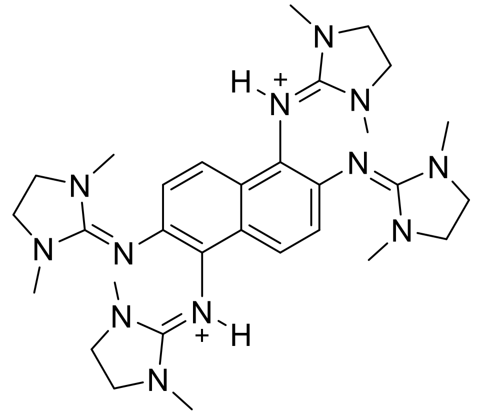

$[PF_6^-]_2$

^{1}H NMR spectrum (600 MHz, CD_3CN) of (**5**+2H)(PF_6)$_2$.

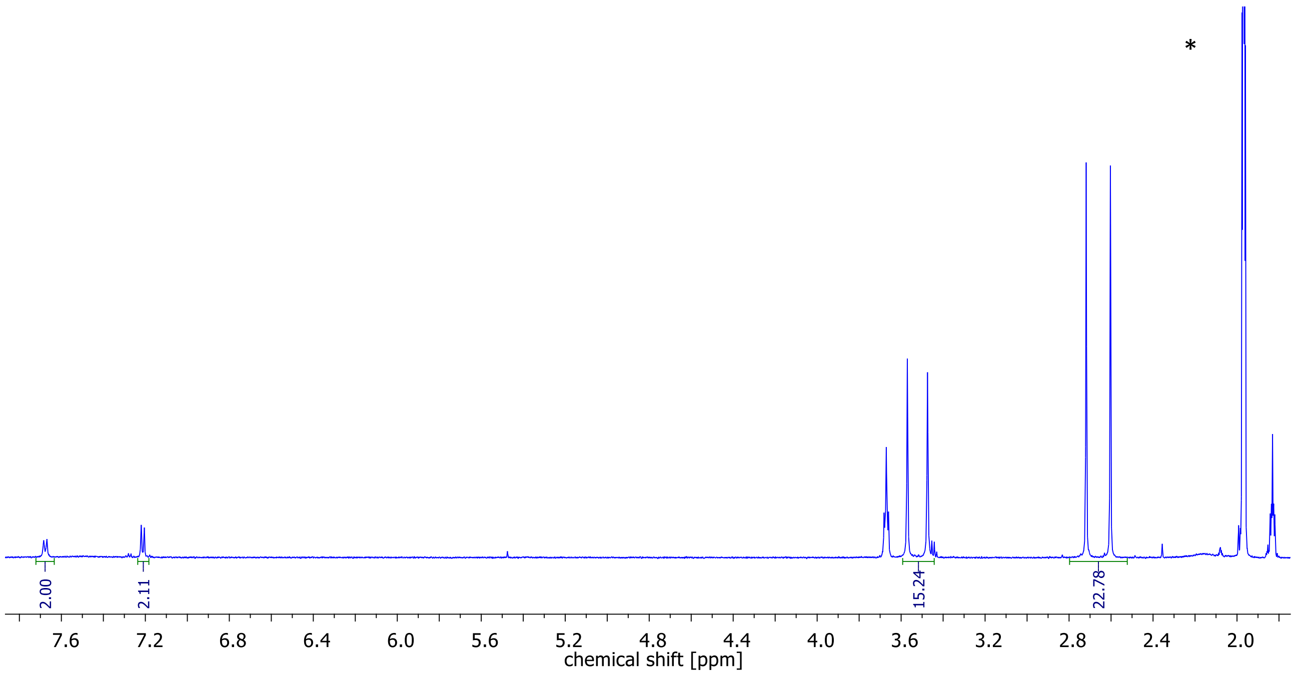

The peak highlighted by an asterisk is due to the solvent.

^{13}C NMR spectrum (600 MHz, CD_3CN) of (**5**+2H)$(PF_6)_2$.

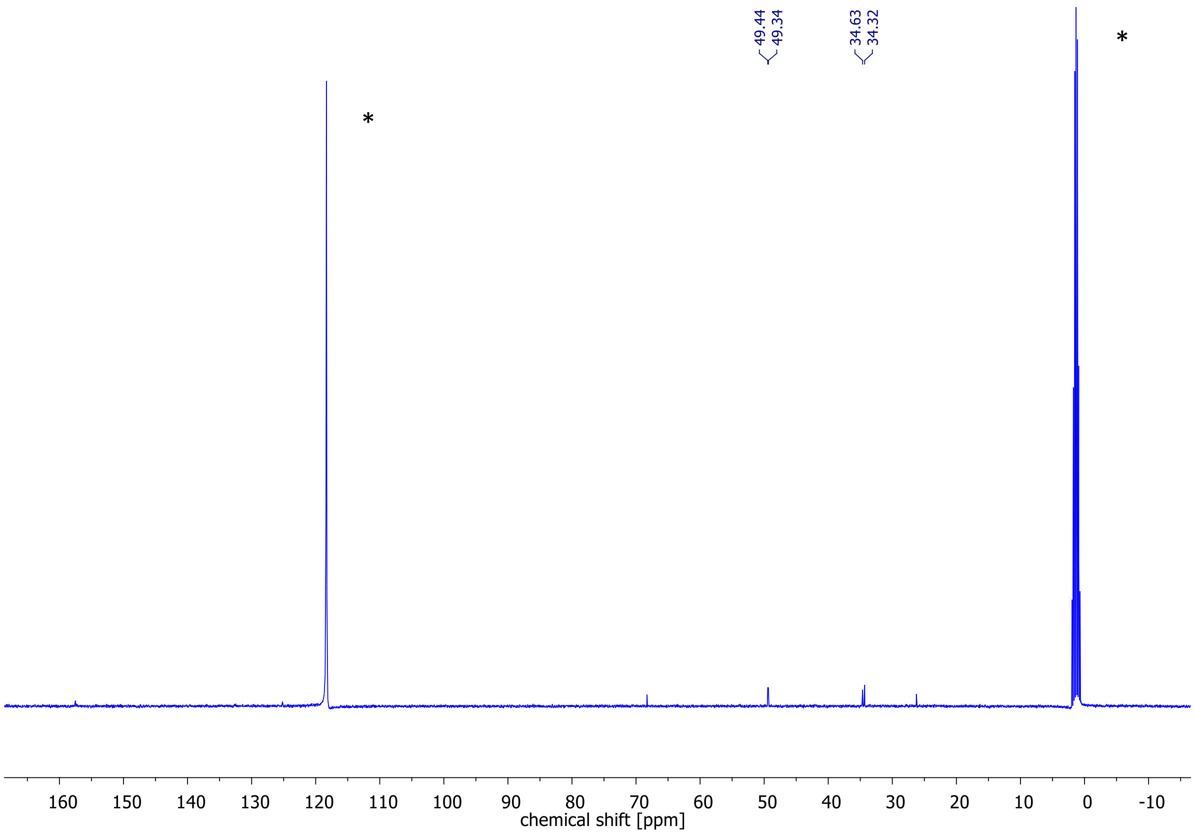

The peaks highlighted by an asterisk are due to the solvent. Peaks at 26.27 ppm and 68.33 ppm are due to THF (see ^{1}H NMR spectrum of (**5**+2H)$(PF_6)_2$).

Molecular structure of (**5**+2H)$(BF_4)_2$. Hydrogen atoms bound to carbon omitted. Displacement ellipsoids drawn at the 50% probability level.

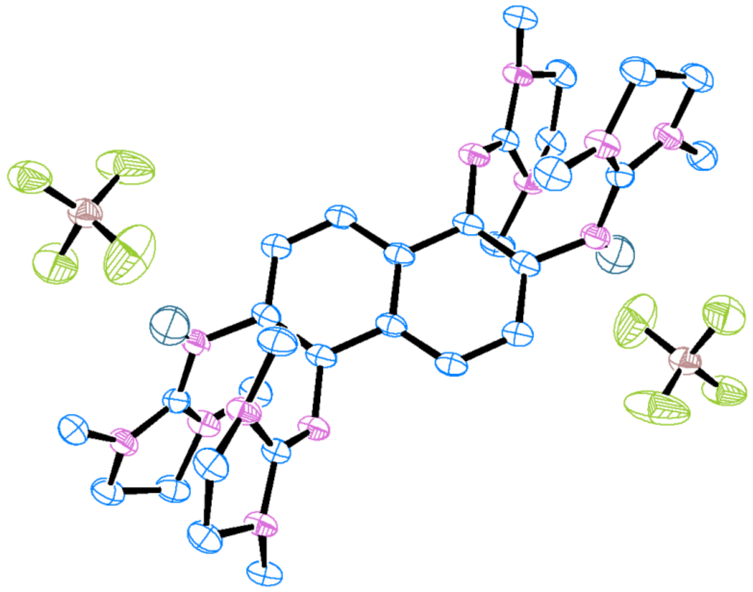

($\mathbf{5}$+4H)Cl_4

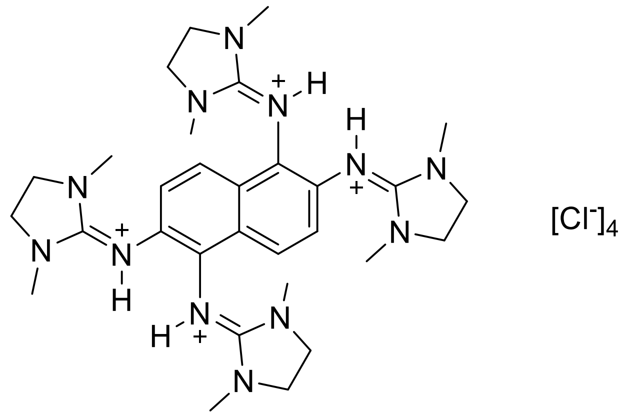

^{1}H NMR spectrum (200 MHz, CD_3CN) of ($\mathbf{5}$+4H)Cl_4

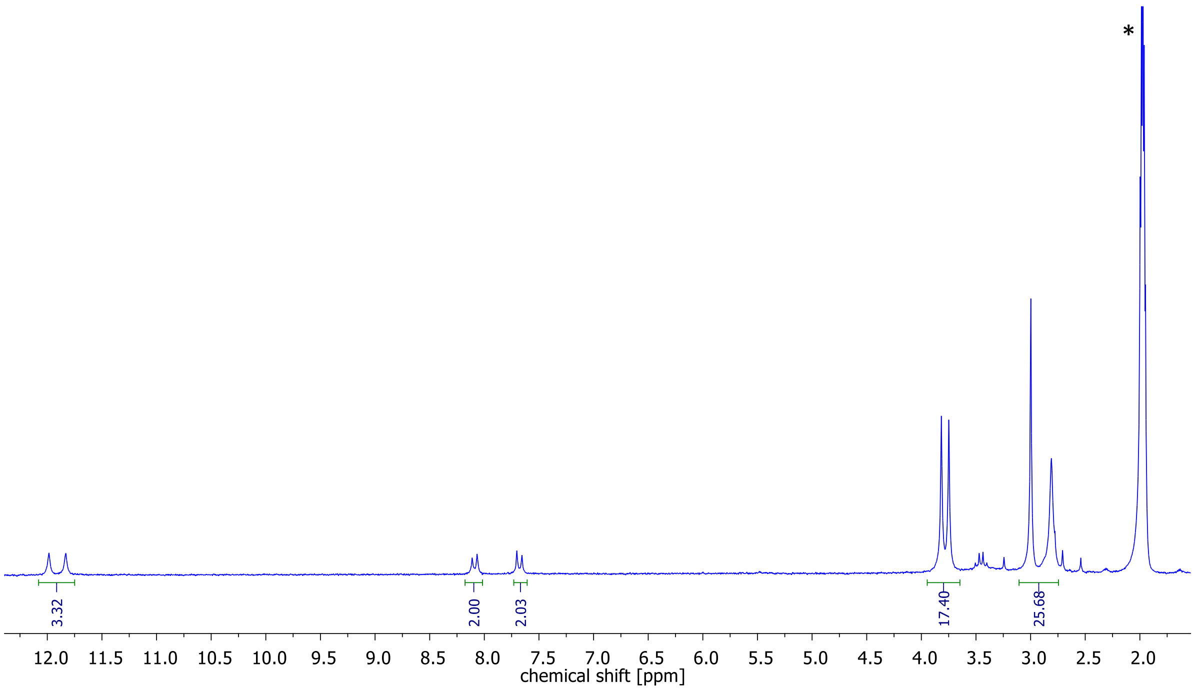

The peak highlighted by an asterisk is due to the solvent.

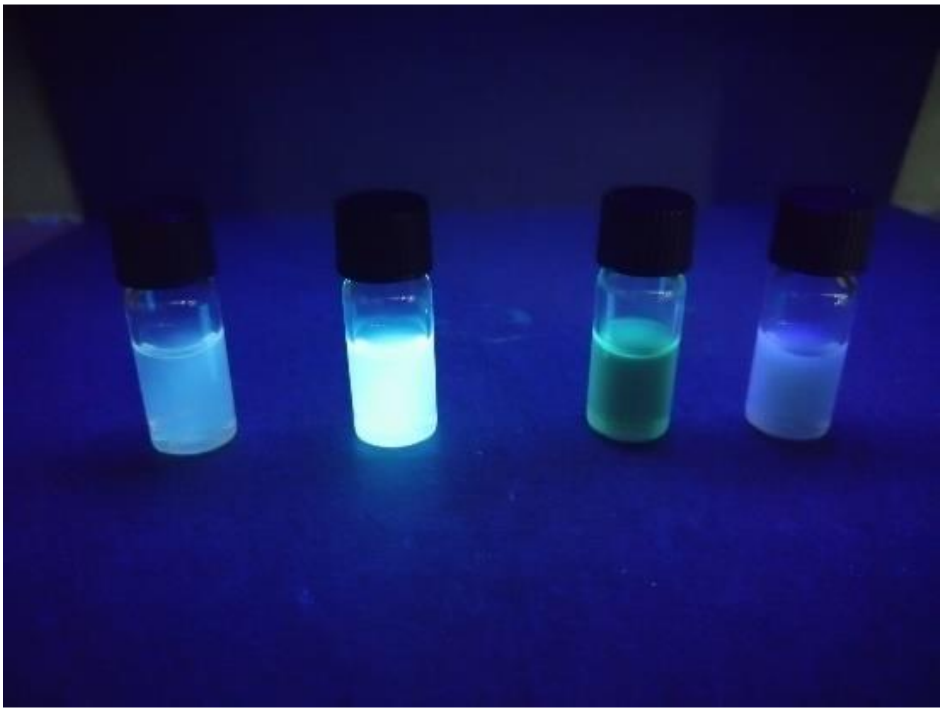

From left to right: Fluorescence of the compounds (**5**+2H)$(PF_6)_2$, (**5**+4H)Cl_4 (**4**+2H)$(PF_6)_2$, (**4**+4H)Cl_4. λ_{ex} = 254 nm.

5) Analytical data for the complexes [5(ZnCl$_2$)$_2$] and [5{Pd(OAc)$_2$}$_2$]

[**5**(ZnCl$_2$)$_2$]

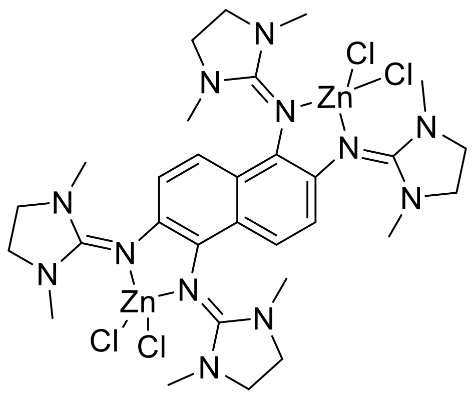

UV-Vis spectrum (CH$_2$Cl$_2$) of compound [**5**(ZnCl$_2$)$_2$].

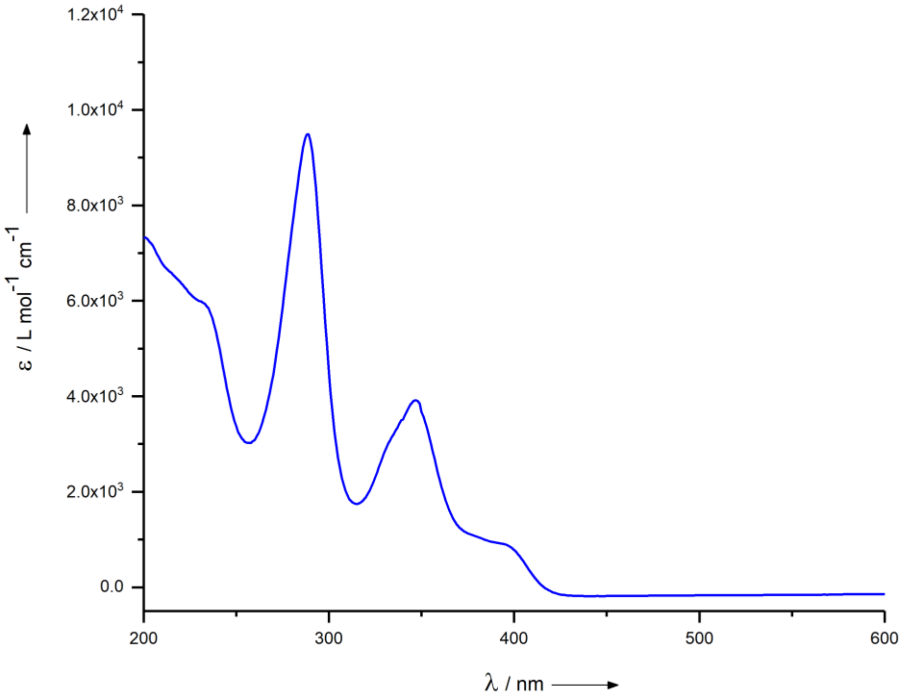

CV curves of compound [**5**($ZnCl_2$)$_2$] in CH_2Cl_2 (Ag/AgCl reference electrode, 0.1 M N(*n*Bu)$_4$(PF_6) as supporting electrolyte, scan rate 100 mV s^{-1}). Potentials given vs. the Fc^+/Fc redox couple.

First two-electron redox process at E_{ox} = -0.09 V

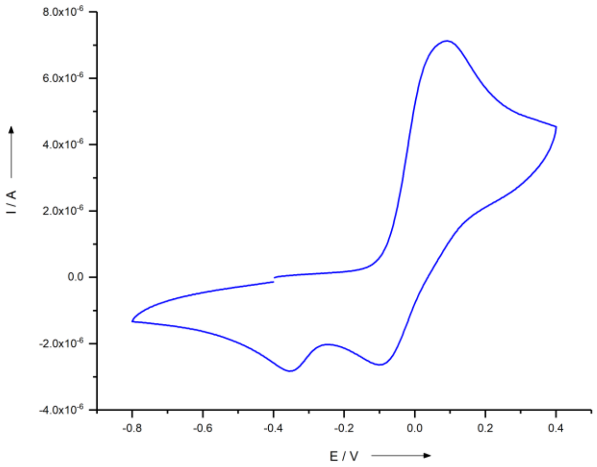

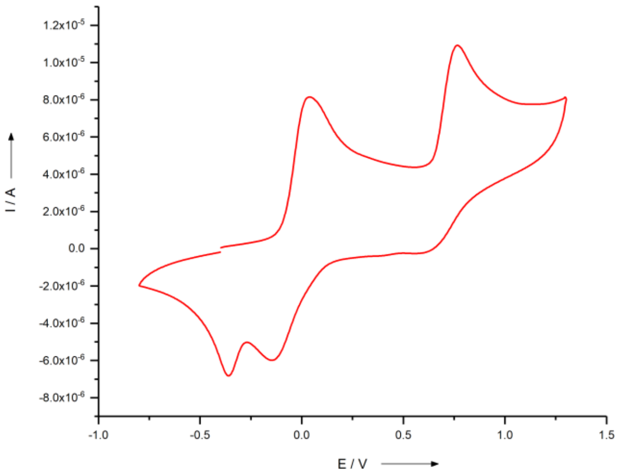

[**5**{Pd(OAc)$_2$}$_2$]

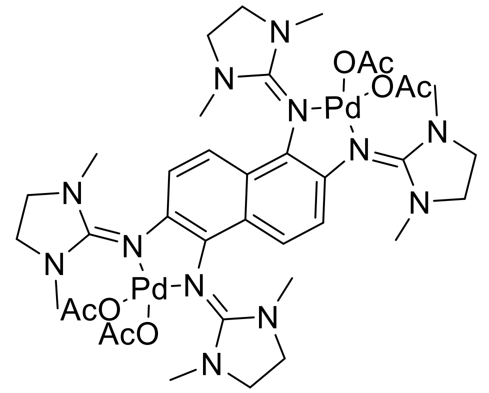

^{1}H NMR spectrum (200 MHz, CD_2Cl_2) of [**5**{Pd(OAc)$_2$}$_2$].

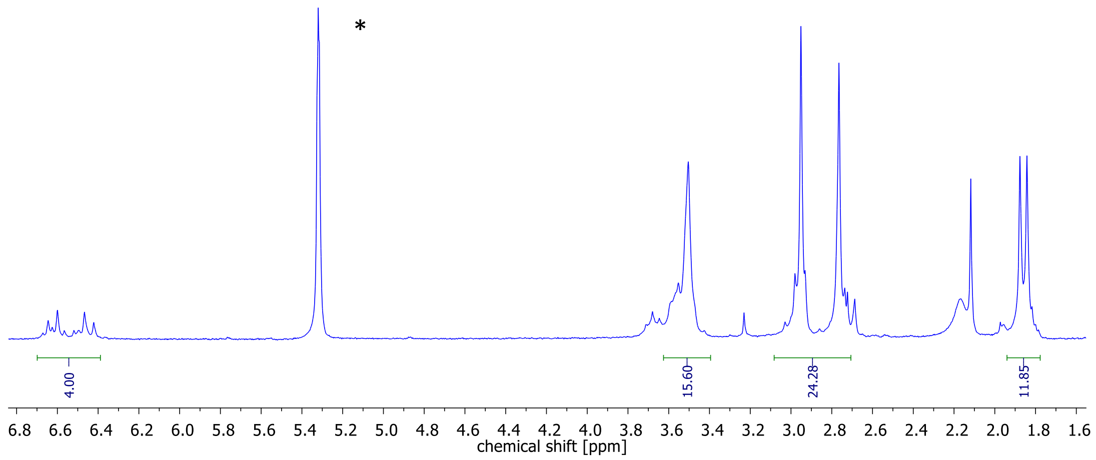

The peak highlighted by an asterisk is due to the solvent.

^{13}C NMR spectrum (600 MHz, CD_2Cl_2) of [**5**{Pd(OAc)$_2$}$_2$].

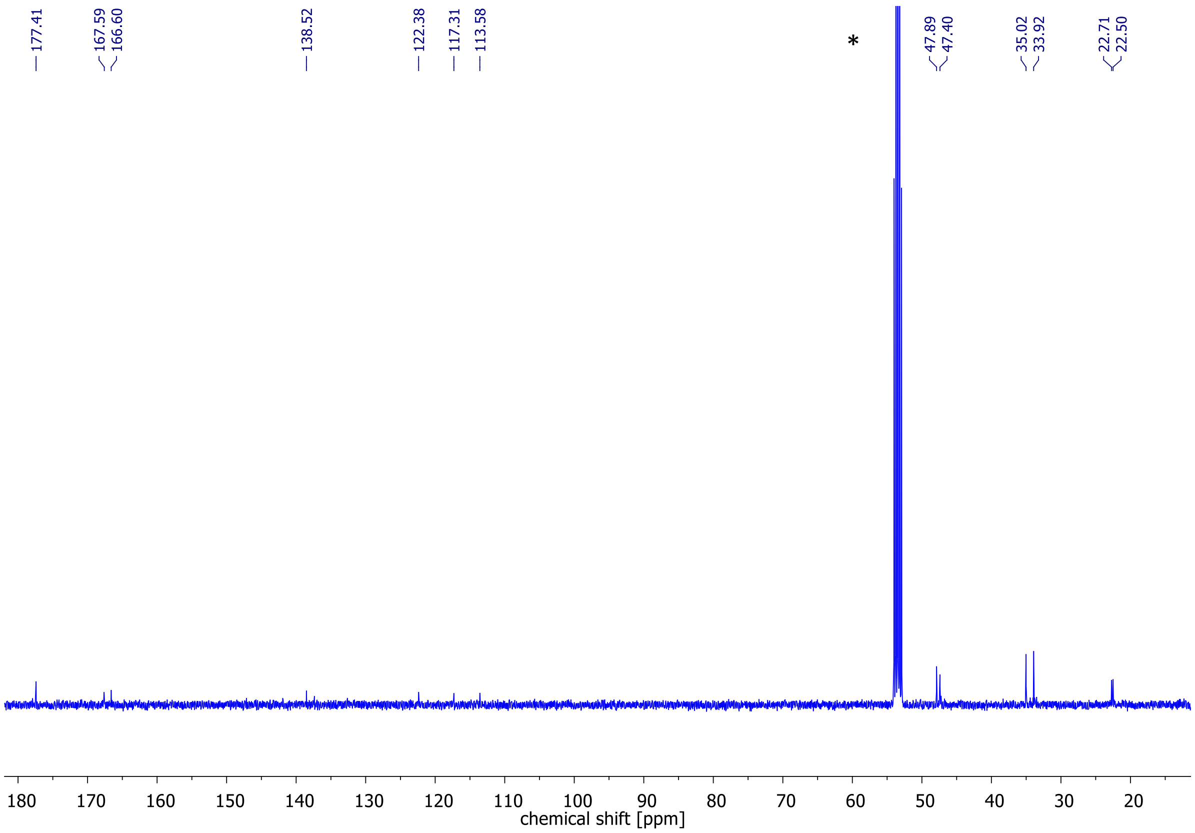

The peak highlighted by an asterisk is due to the solvent.

UV-Vis spectrum (CH_2Cl_2) of compound [**5**{Pd(OAc)$_2$}$_2$]

.

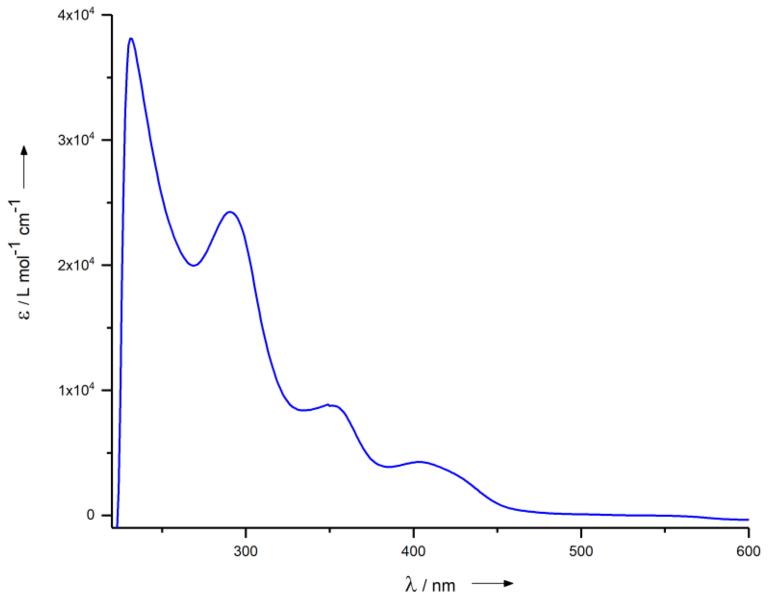

Illustration of the molecular structure of [**5**{Pd(OAc)$_2$}$_2$] (hydrogen atoms omitted for clarity, displacement ellipsoids drawn at the 50% probability level).

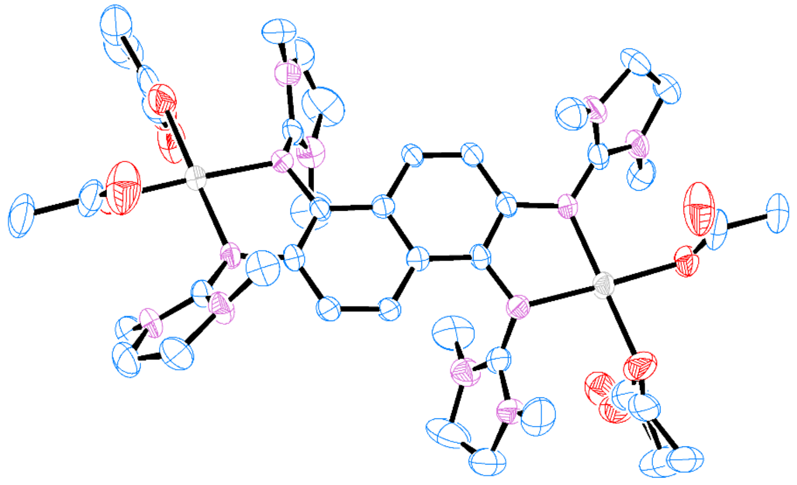

CV curves of **5**{Pd(OAc)$_2$}$_2$ in CH_2Cl_2 (Ag/AgCl reference electrode, 0.1 M N(*n*Bu)$_4$(PF_6) as supporting electrolyte, scan rate 100 mV s^{-1}). Potentials given vs. the Fc^+/Fc redox couple.

First two-electron redox process at $E_{1/2}$ = -0.31 V

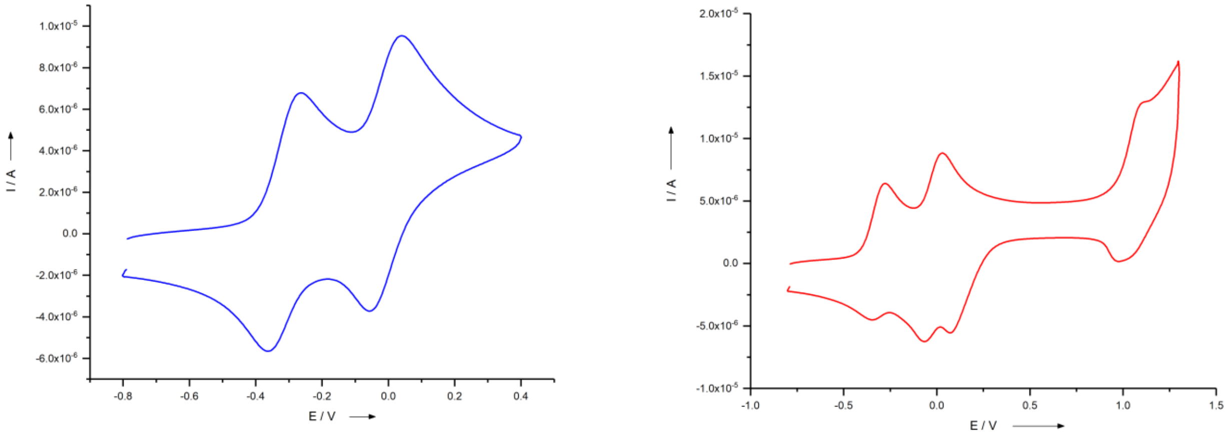

UV-VIS coupled CV measurement of [**5**{Pd(OAc)$_2$}$_2$] in CH_2Cl_2 (Ag/AgCl reference electrode, 0.5 M N(*n*Bu)$_4$(PF_6) as supporting electrolyte, scan speed 10 mV s^{-1}). 3D-Plot (left) and heatmap (right) of three redox cycles.

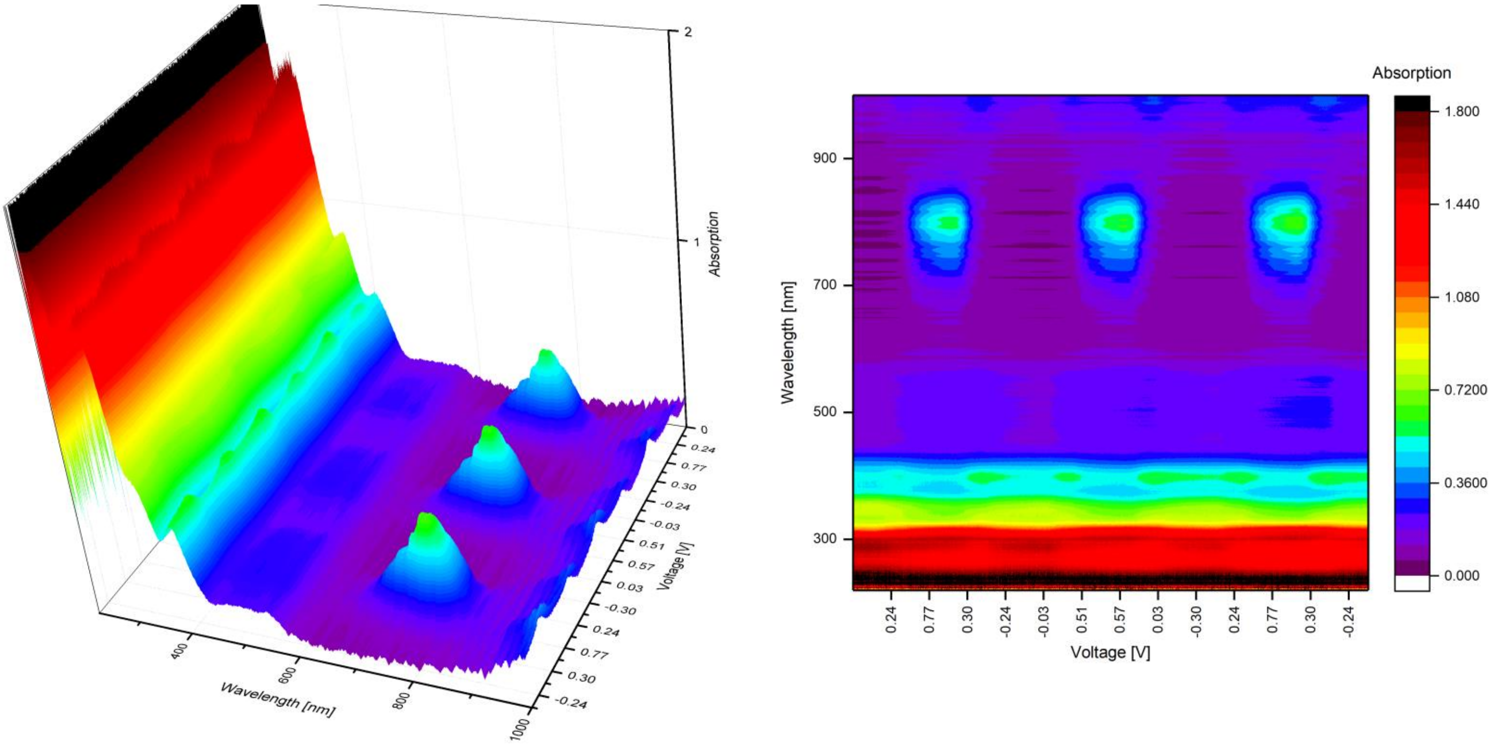

6) Analytical data for the compounds 6-8

1,2,5,6-Tetrakis(dimethylethylene-guanidino)(3,7-dibromo)(4,8-disuccinimido)naphthalene (6)

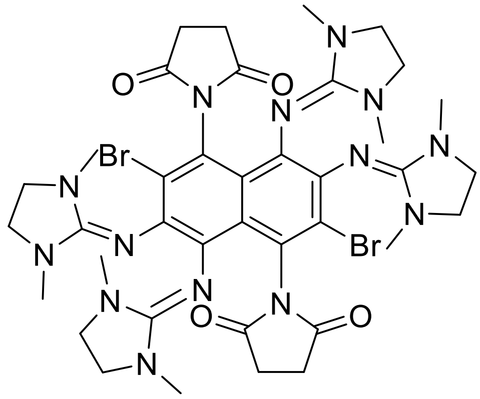

^{1}H NMR spectrum (200 MHz, CD_2Cl_2) of compound **6**.

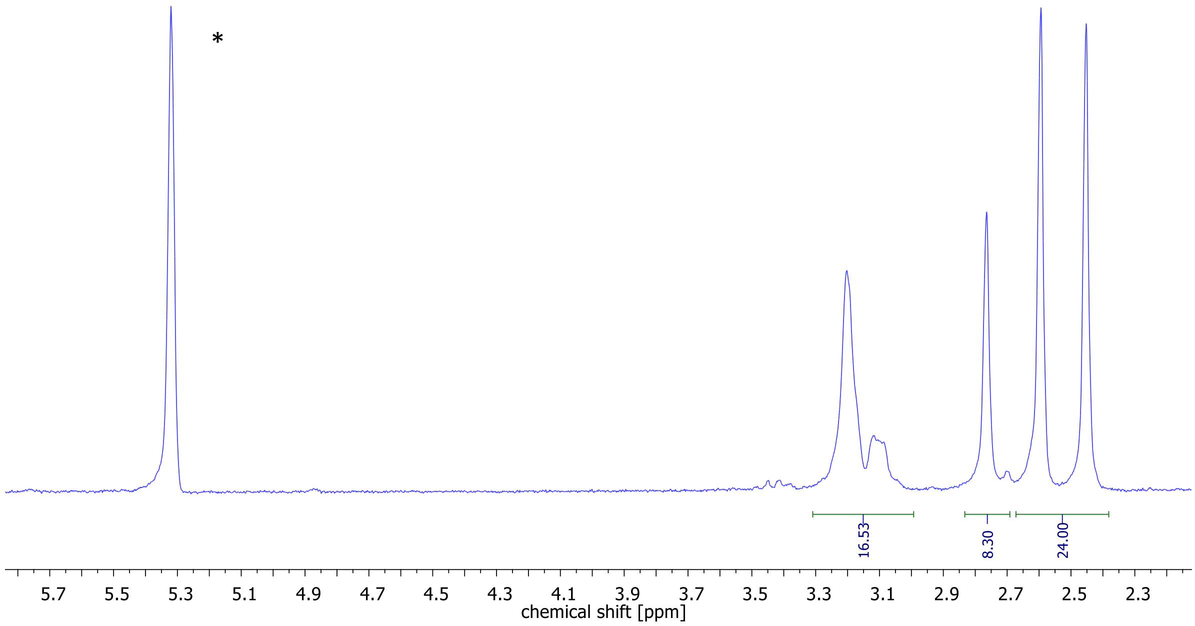

The peak highlighted by an asterisk is due to the solvent.

^{13}C NMR spectrum (600 MHz, CD_2Cl_2) of compound **6**.

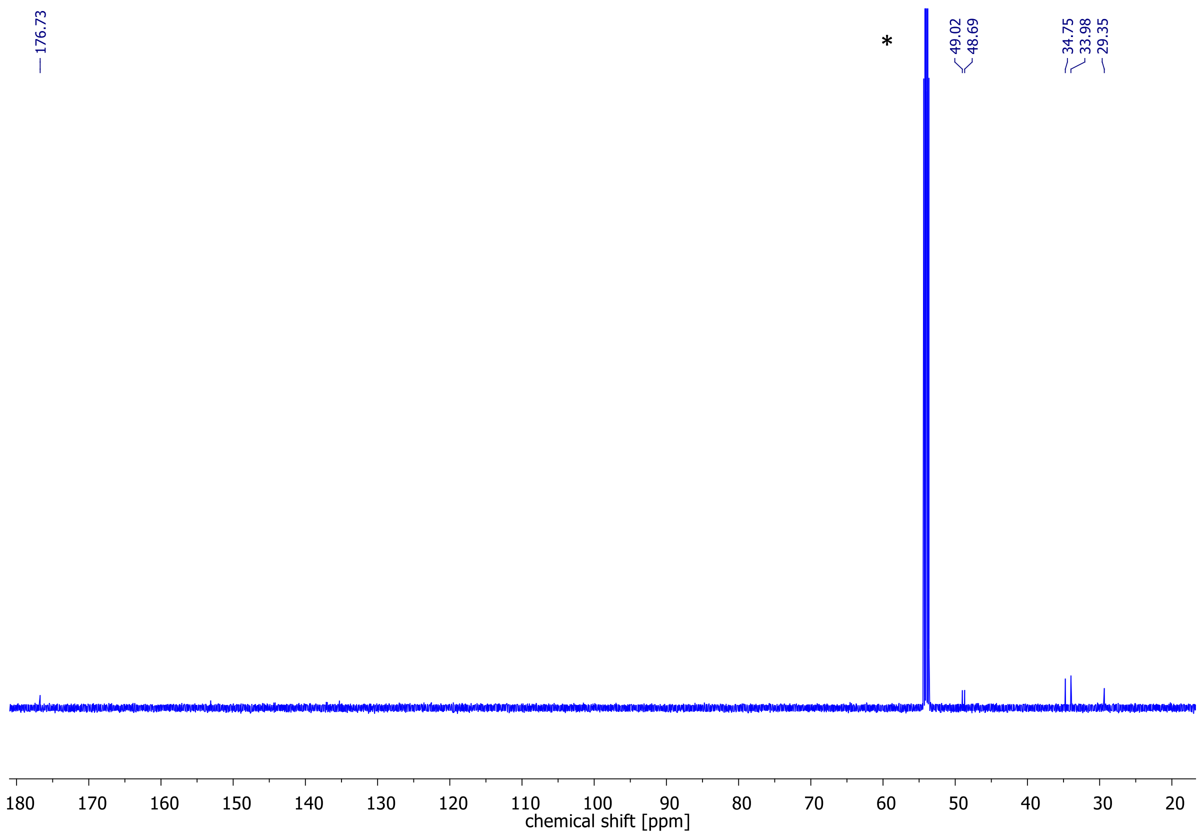

The peak highlighted by an asterisk is due to the solvent.

UV-Vis spectrum (CH_2Cl_2) of compound **6**.

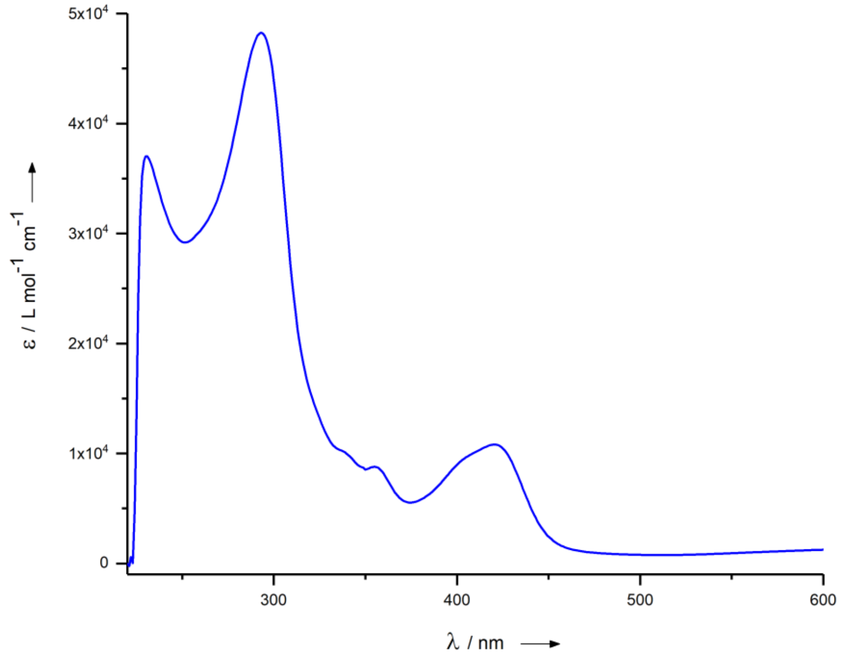

Illustration of the molecular structure of **6** (hydrogen atoms omitted for clarity, displacement ellipsoids drawn at the 50% probability level).

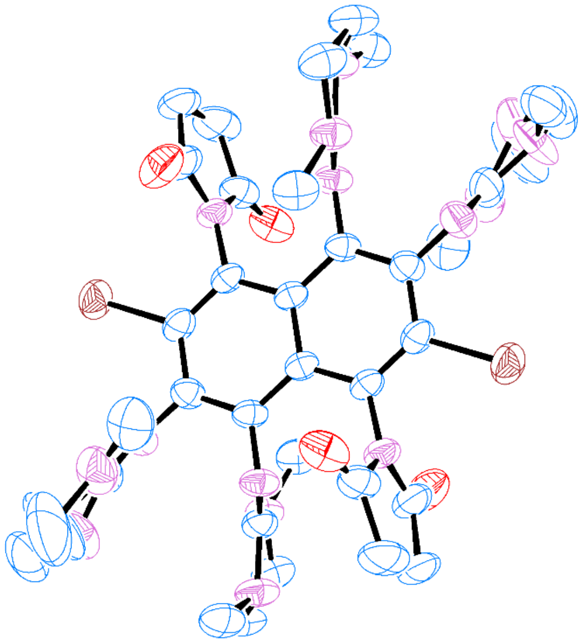

CV curves of **6** in CH_2Cl_2 (Ag/AgCl reference electrode, 0.1 M N(*n*Bu)$_4$(PF_6) as supporting electrolyte, scan rate 100 mV s^{-1}). Potentials given vs. the Fc^+/Fc redox couple.

First two-electron redox process at $E_{1/2}$ = -0.30 V

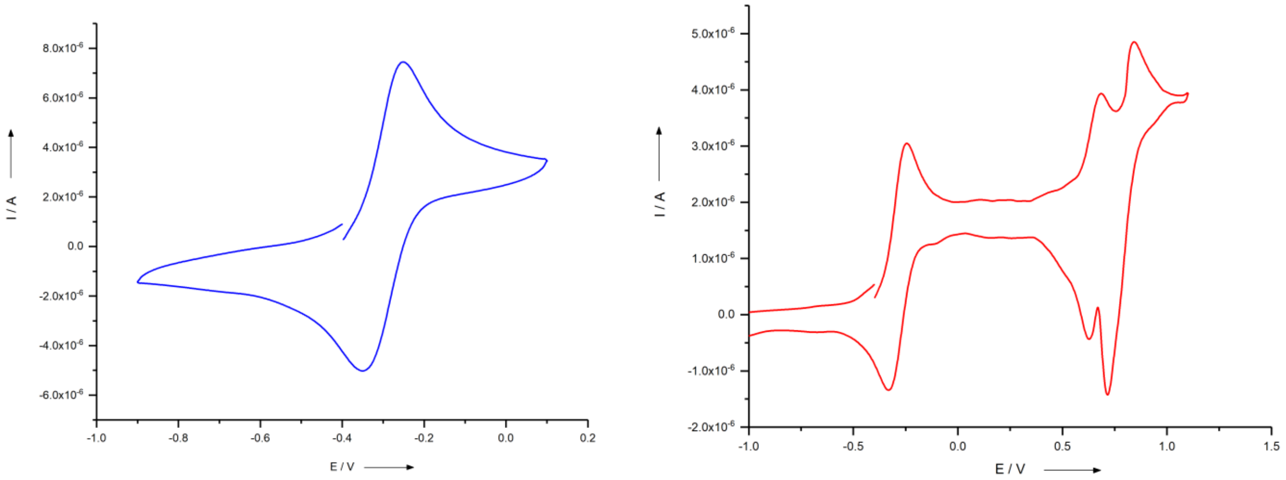

1,2,5,6-Tetrakis(dimethylethylene-guanidino)(3,7-diiodo)(4,8-disuccinimido)-naphthalene (7)

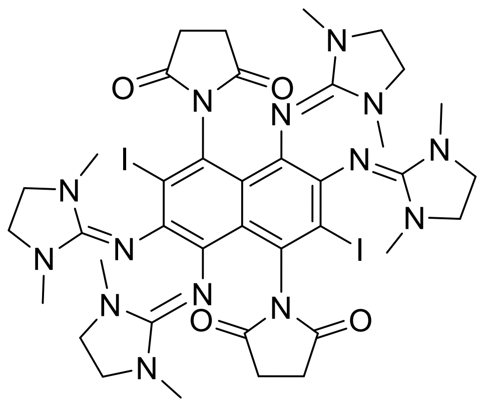

^{1}H-NMR spectrum (200 MHz, CD_2Cl_2) of compound **7**.

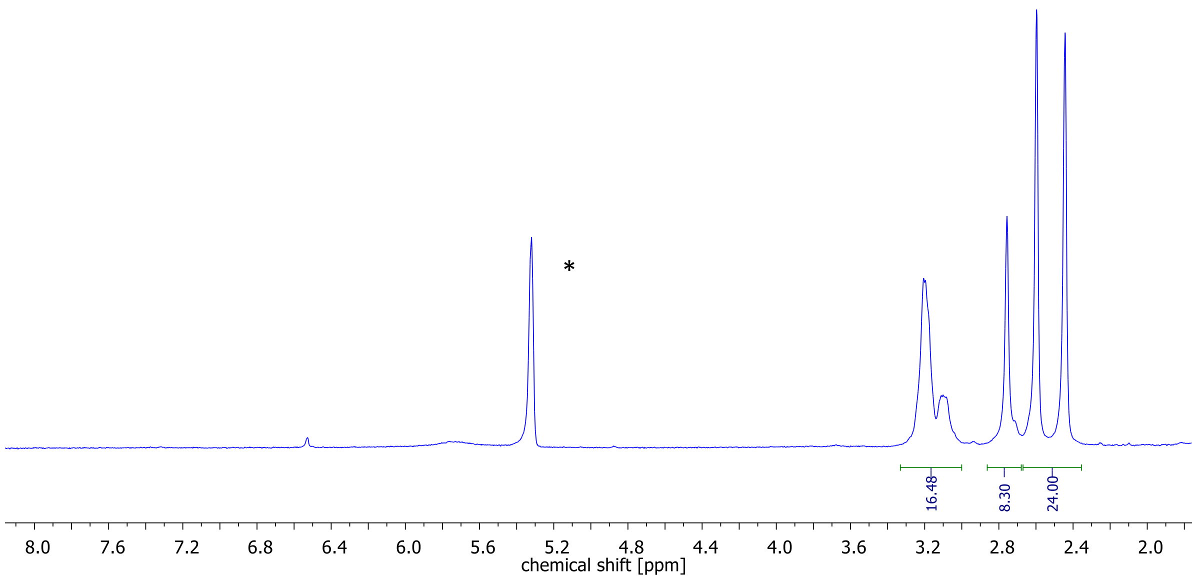

The peak highlighted by an asterisk is due to the solvent.

^{13}C-NMR spectrum (200 MHz, CD_2Cl_2) of compound **7**.

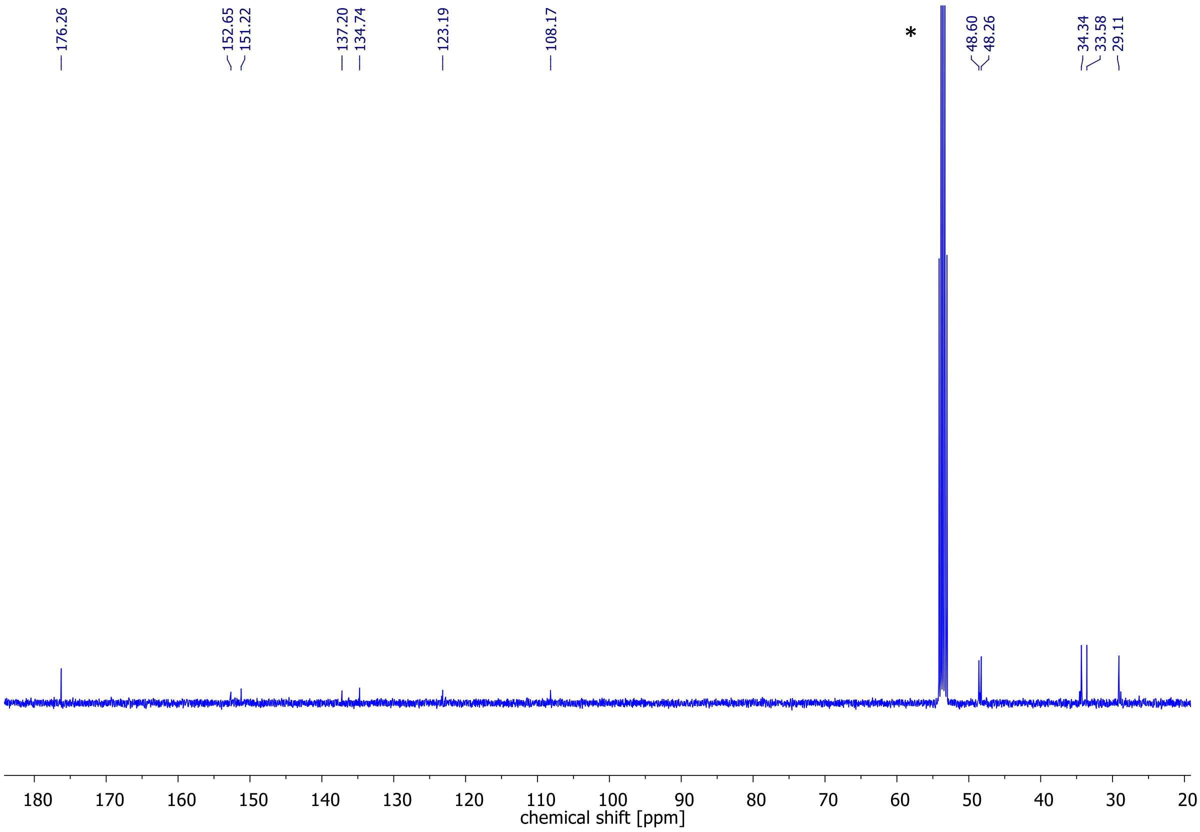

The peak highlighted by an asterisk is due to the solvent.

UV-Vis spectrum (CH_2Cl_2) of compound **7**.

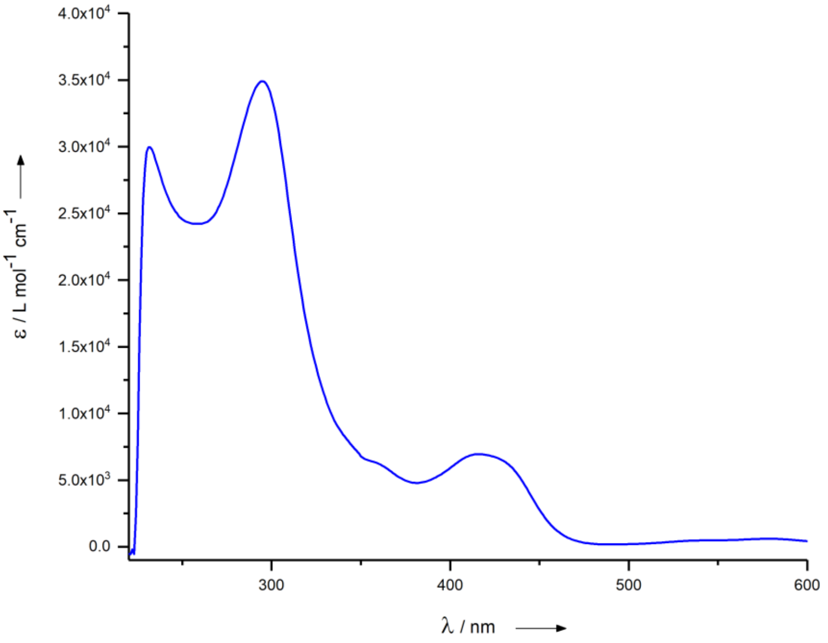

Illustration of the molecular structure of **7**. Hydrogen atoms omitted. Displacement ellipsoids drawn at the 50% probability level.

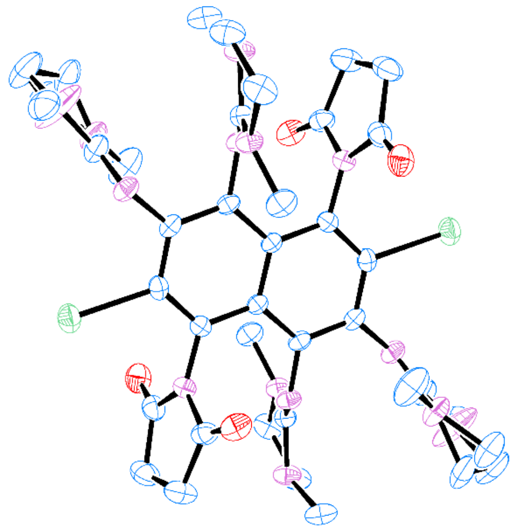

CV curves of **7** in CH_2Cl_2 (Ag/AgCl reference electrode, 0.1 M N(*n*Bu)$_4$(PF_6) as supporting electrolyte, scan rate 100 mV s^{-1}). Potentials given vs. the Fc^+/Fc redox couple.

First two-electron redox process at E_{ox} = -0.18 V

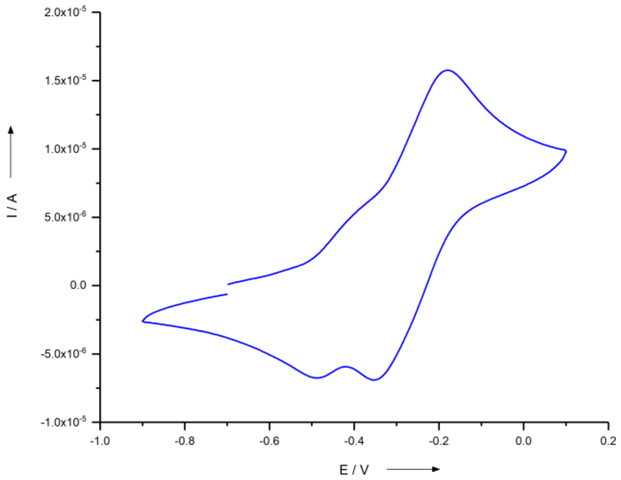

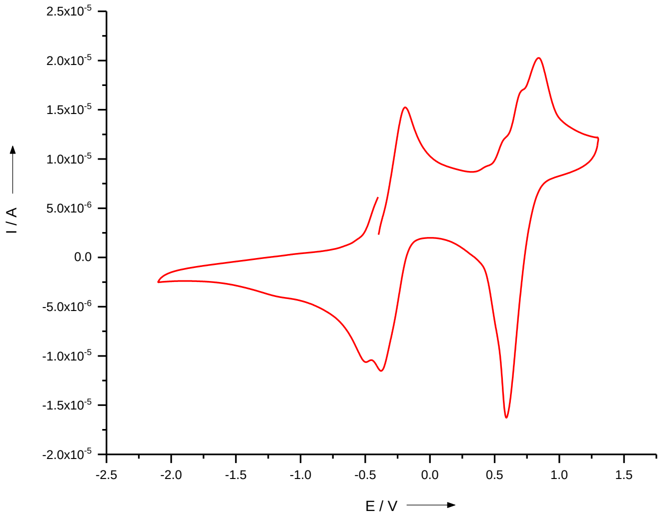

1,2,5,6-Tetrakis(dimethylethylene-guanidino)(3,7-dibromo)(4,8-diphthalimido)-naphthalene (8)

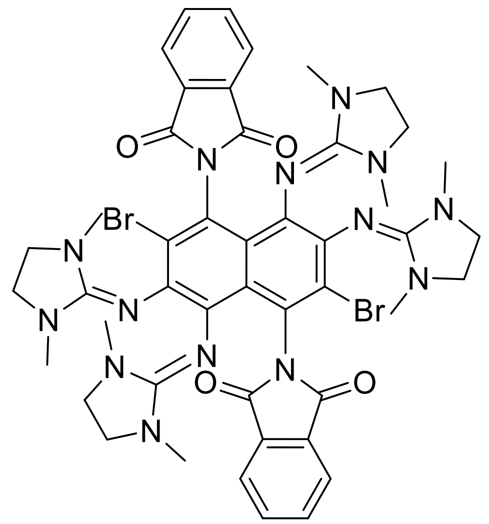

^{1}H NMR spectrum (200 MHz, CD_2Cl_2) of compound **8**.

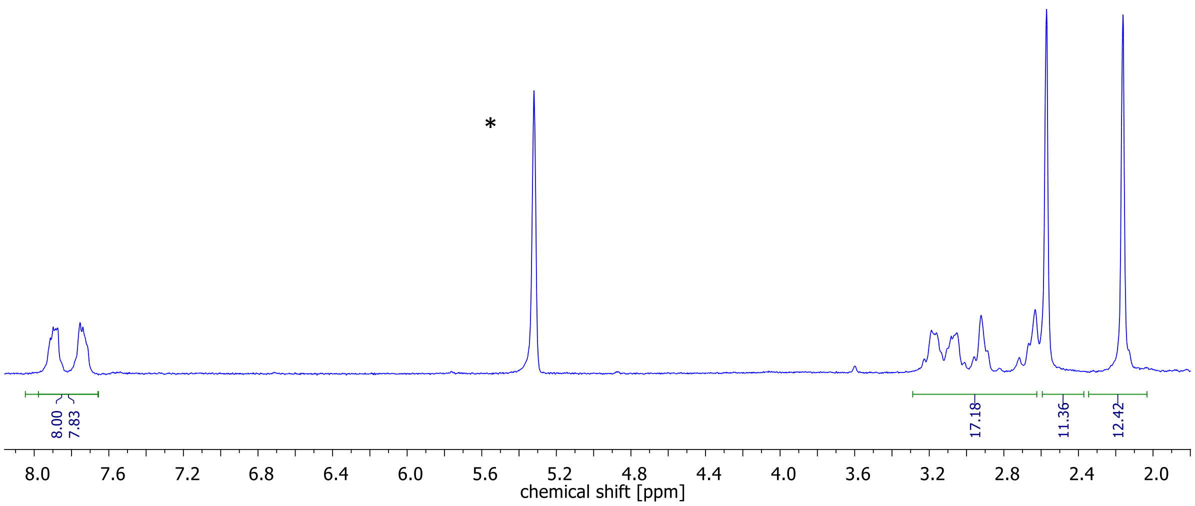

The peak highlighted by an asterisk is due to the solvent.

^{13}C NMR spectrum (600 MHz, CD_2Cl_2) of compound **8**.

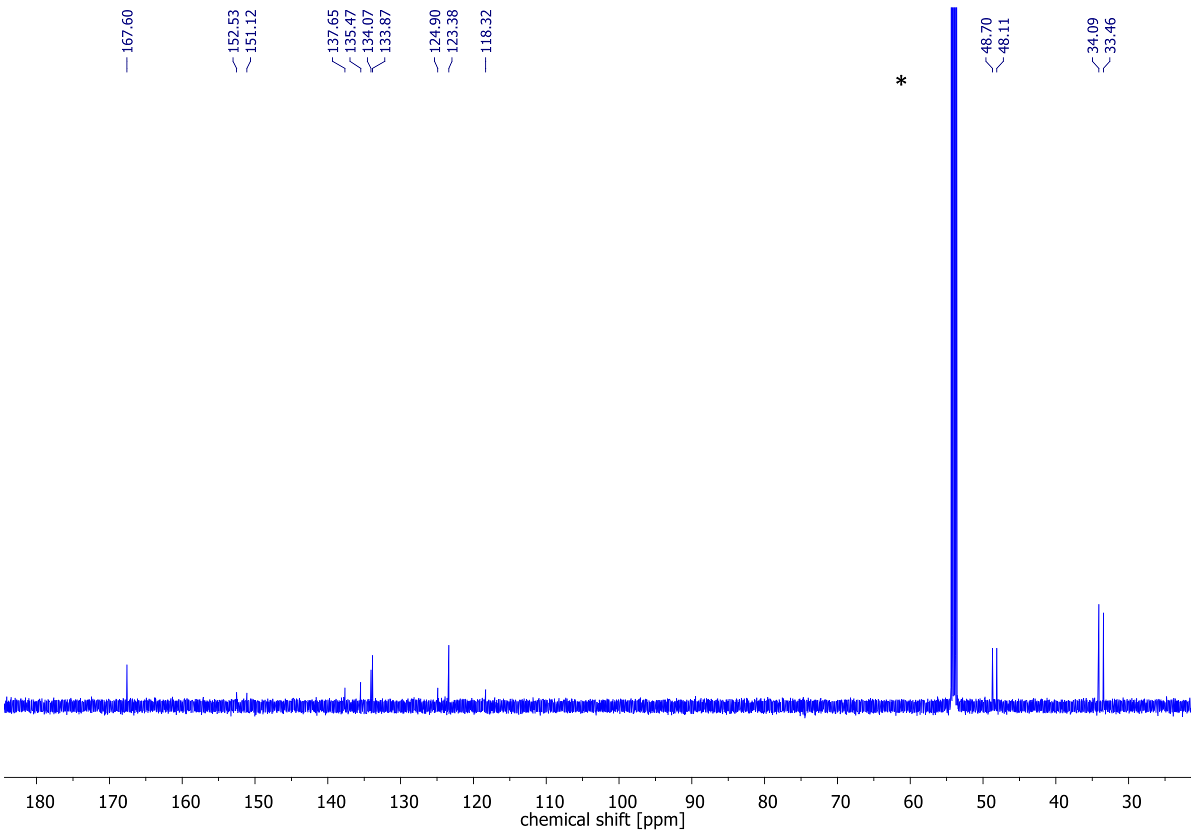

The peak highlighted by an asterisk is due to the solvent.

UV-Vis spectrum (CH_2Cl_2) of compound **8**.

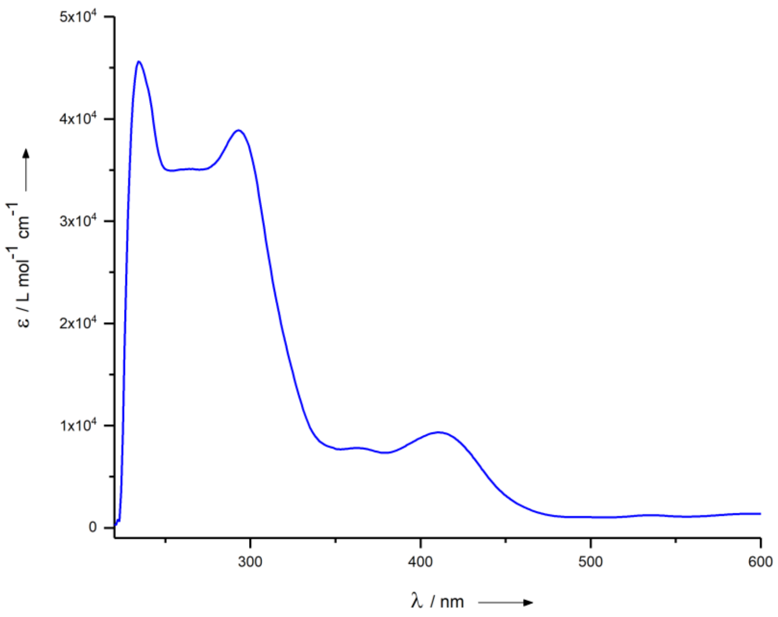

Illustration of the molecular structure of **8**. Hydrogen atoms omitted. Displacement ellipsoids drawn at the 50% probability level.

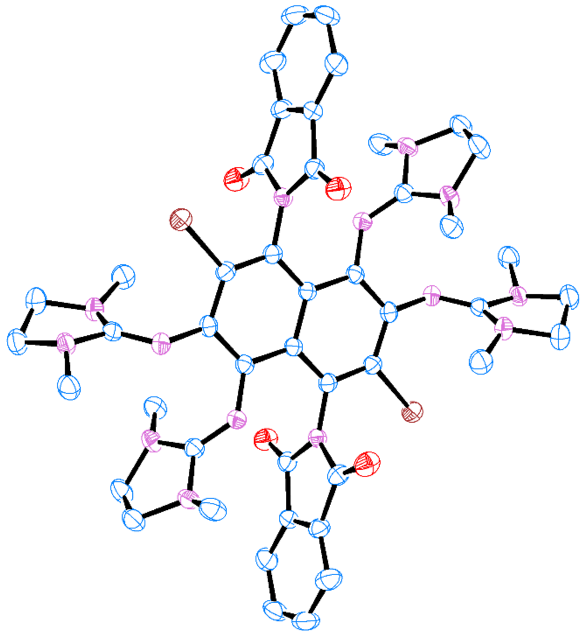

CV curves of **8** in CH_2Cl_2 (Ag/AgCl reference electrode, 0.1 M N(*n*Bu)$_4$(PF_6) as supporting electrolyte, scan rate 100 mV s^{-1}). Potentials given vs. the Fc^+/Fc redox couple.

First two-electron redox process at $E_{1/2}$ = -0.25 V

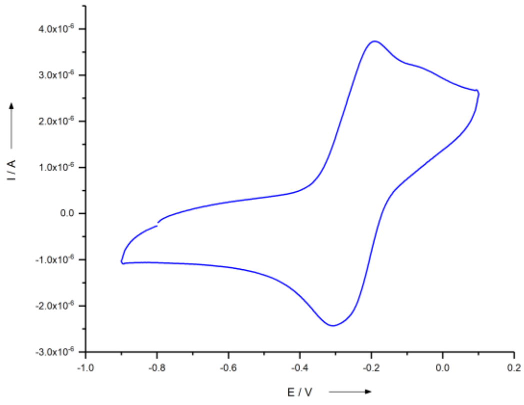

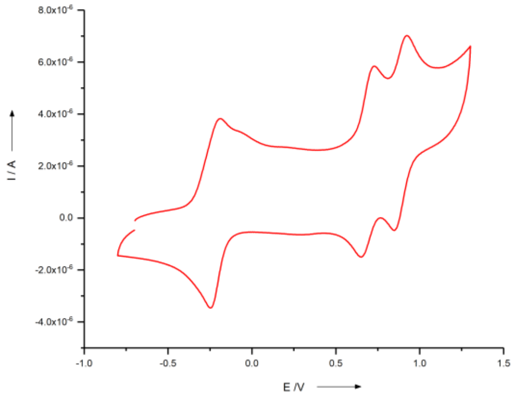

7) Details of the crystal structure determinations

	N,*N*'-(1,5-dinitronaphthalene-2,6-diyl)bis(1,1-diphenylmethanimine)	**4**	**5**	**5**$(SbF_6)_2$
CCDC-No.	1968385	1968384	1968386	1968393
formula	$C_{36}H_{24}N_4O_4$	$C_{30}H_{52}N_{12}$	$C_{30}H_{44}N_{12}$	$C_{30}H_{44}F_{12}N_{12}Sb_2$
Crystal system	monoclinic	monoclinic	monoclinic	monoclinic
Space group	$P\,2_1/n$	$P\,2_1/n$	$P\,2_1/c$	$P\,2_1/n$
a /Å	8.4230(17)	11.1213(4)	8.3460(17)	7.6425(17)
b /Å	18.986(4)	11.1328(3)	26.422(5)	19.015(4)
c /Å	9.4380(19)	14.1098(5)	14.835(3)	14.478(3)
α /°	90	90	90	90
β /°	103.26(3)	110.198(4)	104.22(3)	104.417(7)
γ /°	90	90	90	90
V /Å^3	1469.1(5)	1639.54(10)	3171.2(12)	2037.7(8)
Z	2	2	4	2
M_r	576.59	580.83	608.80	1044.27
F_{000}	600	632	1312	1036
d_c /Mg·m^{-3}	1.303	1.177	1.275	1.702
μ /mm^{-1}	0.087	0.075	0.085	1.420
max., min. transmission factors	0.966, 0.974	1.0000, 0.9604	1.000, 0.6316	0.7461, 0.5623
X-radiation, λ /Å	Mo-K_a, 0.71073	Mo-K_a, 0.71073	Mo-K_a 0.71073	Mo-K_a 0.71073
data collect. temperat./K	120	120(1)	120	100
θ range /°	2.145 to 30.032	2.390 to 32.413	2.093 to 28.499	2.142 to 25.999
index ranges *h,k,l*	-11 … 11, -26 … 26, -13 … 13	-16 ... 16, -16 ... 16, -20 ... 21	-11 ... 11, -35 ... 35, -19 ... 19	-9 ... 8, -23 ... 23, -16 ... 17
reflections measured	8372	60357	59537	16860
Unique [R_{int}]	4282 [0.0314]	5728 [0.0850]	8033 [0.0718]	3988 [0.0994]
observed [$I \geq 2\sigma(I)$]	3331	3967	5265	2751
data / restraints /parameters	4282/ 0 / 199	5728 / 0 / 212	8033/ 0 / 411	3988/ 9 / 257
GooF on F^2	1.025	1.023	1.027	1.087
R indices[$F>4\sigma(F)$] $R(F)$, $wR(F^2)$	0.0441, 0.1123	0.0560, 0.1223	0.0535, 0.1141	0.0858, 0.1826
R indices (all data) $R(F)$, $wR(F^2)$	0.0604, 0.1237	0.0910, 0.1385	0.0980, 01356	0.1212, 0.1991
largest residual peaks /e·Å^{-3}	0.270, -0.216	0.430, -0.248	0.444, -0.488	2.632, -1.988

	(**5**+2H)(BF_4)	[**5**{Pd(OAc)$_2$}$_2$]	**6**	**7**
CCDC-No.	1968391	1968390	1968387	1968388
formula	$C_{30}H_{46}B_2F_8N_{12}$	$C_{38}H_{56}N_{12}O_8Pd_2$	$C_{38}H_{48}Br_2N_{14}O_4$	$C_{38}H_{48}I_2N_{14}O_4$
Crystal system	monoclinic	orthorhombic	monoclinic	monoclinic
Space group	$P\ 2_1/n$	*P*ccn	$P\ 2_1/c$	$P\ 2_1/c$
a /Å	8.7572(5)	16.9160(12)	9.2180(18)	9.2770(19)
b /Å	18.4122(11)	22.1807(14)	10.650(2)	11.089(2)
c /Å	11.1688(7)	12.6227(10)	21.005(4)	20.862(4)
α /°	90	90	90	90
β /°	106.755(2)	90	98.33(3)	98.85(3)
γ /°	90	90	90	90
V /Å^3	1724.40(18)	4736.1(6)	2040.3(7)	2120.6(8)
Z	2	4	2	2
M_r	748.41	1021.74	924.27	1018.70
F_{000}	784	2096	952	1024
d_c /Mg·m^{-3}	1.441	1.433	1.505	1.595
μ /mm^{-1}	0.120	0.818	2.046	1.540
max., min. transmission factors	0.7457, 0.6535	0.7456, 0.6106	1.00, 0.3343	1.00, 0.6256
X-radiation, λ /Å	Mo-K_a 0.71073	Mo-K_a 0.71073	Mo-K_a 0.71073	Mo-K_a 0.71073
data collect. temperat./K	120	120	120	120
θ range /°	2.619 to 28.878	4.43 to 52.00	3.92 to 60.33	3.95 to 60.23
index ranges *h,k,l*	-11 ... 11, -23 ... 24, -14 ... 14	-20 ... 20, -27 ... 27, -15 ... 15	-12 ... 13, -15 ... 14, -29 ... 29	-12 ... 13, -11 ... 15, -29 ... 29
reflections measured	26411	90046	39952	26876
Unique [R_{int}]	4411 [0.0490]	4659 [0.1620]	5965 [0.0875]	6165 [0.0497]
observed [$I \geq 2\sigma(I)$]	2970	3160	3122	4649
data / restraints /parameters	4411/ 0 / 243	4659 / 84 / 306	5965 / 3 / 286	6165 / 0 / 275
GooF on F^2	1.079	1.050	1.034	1.080
R indices[$F>4\sigma(F)$] $R(F)$, $wR(F^2)$	0.0582, 0.1616	0.0595, 0.1298	0.0707, 0.1179	0.0455, 0.1099
R indices (all data) $R(F)$, $wR(F^2)$	0.0920, 0.1804	0.0956, 0.1467	0.1433, 0.2167	0.0740, 0.1217
largest residual peaks /e·Å^{-3}	0.536, -0.459	1.06, -0.78	2.32, -0.96	0.97, -0.93

	**8**	Intermediate of **7**
CCDC-No.	1968389	1968392
formula	$C_{46}H_{48}Br_2N_{14}O_4$	$C_{19}H_{25}I_3N_7O_2$
Crystal system	orthorhombic	triclinic
Space group	*P*bca	$P\bar{1}$
a /Å	13.945(3)	8.4283(7)
b /Å	14.930(3)	12.4724(10)
c /Å	21.644(4)	12.9895(10)
α /°	90	87.328(3)
β /°	90	82.727(3)
γ /°	90	78.961(3)
V /$Å^3$	4506.3(16)	1328.70(19)
Z	4	2
M_r	1020.80	764.16
F_{000}	2096	726
d_c /Mg·m^{-3}	1.505	1.910
μ /mm^{-1}	1.861	3.556
max., min. transmission factors	1.00, 0.4096	0.7455, 0.6012
X-radiation, λ /Å	Mo-K_a 0.71073	Mo-K_a 0.71073
data collect. temperat./K	120	100
θ range /°	4.76 to 57.00	4.54 to 55.00
index ranges *h,k,l*	-18 ... 18, -18 ... 20, -29 ... 29	-10 ... 10, -16 ... 16, -16 ... 16
reflections measured	62531	28649
Unique [R_{int}]	5700 [0.1141]	6081 [0.0838]
observed [$I \geq 2\sigma(I)$]	3944	3742
data / restraints /parameters	5700 / 0 / 302	6081 / 0 / 284
GooF on F^2	1.093	1.058
R indices[$F>4\sigma(F)$] $R(F)$, $wR(F^2)$	0.0539, 0.1184	0.0535, 0.1308
R indices (all data) $R(F)$, $wR(F^2)$	0.0881, 0.1363	0.0925, 0.1518
largest residual peaks /e·$Å^{-3}$	0.91, -0.51	2.84, -1.72
